# New insights into the competition between antioxidant activities and pro-oxidant risks of rosmarinic acid†

**DOI:** 10.1039/d1ra07599c

**Published:** 2022-01-10

**Authors:** Dinh Hieu Truong, Thi Chinh Ngo, Nguyen Thi Ai Nhung, Duong Tuan Quang, Thi Le Anh Nguyen, Dorra Khiri, Sonia Taamalli, Florent Louis, Abderrahman El Bakali, Duy Quang Dao

**Affiliations:** Institute of Research and Development, Duy Tan University Da Nang 550000 Vietnam daoduyquang@duytan.edu.vn; Faculty of Natural Sciences, Duy Tan University Da Nang 550000 Vietnam; Department of Chemistry, University of Sciences, Hue University Hue 530000 Vietnam; Department of Chemistry, University of Education, Hue University Hue 530000 Vietnam; Université de Lille, CNRS, UMR 8522 – PC2A – PhysicoChimie des Processus de Combustion et de l'Atmosphère 59000 Lille France

## Abstract

Direct and indirect antioxidant activities of rosmarinic acid (RA) based on HOO˙/CH_3_OO˙ radical scavenging and Fe(iii)/Fe(ii) ion chelation were theoretically studied using density functional theory at the M05-2X/6-311++G(2df,2p) level of theory. First, four antioxidant mechanisms including hydrogen atom transfer (HAT), radical adduct formation (RAF), proton loss (PL) and single electron transfer (SET) were investigated in water and pentyl ethanoate (PEA) phases. Regarding the free radical scavenging mechanism, HAT plays a decisive role with overall rate coefficients of 1.84 × 10^3^ M^−1^ s^−1^ (HOO˙) and 4.49 × 10^3^ M^−1^ s^−1^ (CH_3_OO˙) in water. In contrast to PL, RAF and especially SET processes, the HAT reaction in PEA is slightly more favorable than that in water. Second, the [Fe(iii)(H_2_O)_6_]^3+^ and [Fe(ii)(H_2_O)_6_]^2+^ ion chelating processes in an aqueous phase are both favorable and spontaneous especially at the O5, site-1, and site-2 positions with large negative Δ_r_*G*^0^ values and great formation constant *K*_f_. Finally, the pro-oxidant risk of RA^−^ was also considered *via* the Fe(iii)-to-Fe(ii) complex reduction process, which may initiate Fenton-like reactions forming reactive HO˙ radicals. As a result, RA^−^ does not enhance the reduction process when ascorbate anions are present as reducing agents, whereas the pro-oxidant risk becomes remarkable when superoxide anions are found. The results encourage further attempts to verify the speculation using more powerful research implementations of the antioxidant activities of rosmarinic acid in relationship with its possible pro-oxidant risks.

## Abstract 2

Direct and indirect antioxidant activities of rosmarinic acid (RA) based on HOO˙/CH_3_OO˙ radical scavenging and Fe(iii)/Fe(ii) ion chelation were theoretically studied using density functional theory at the M05-2X/6-311++G(2df,2p) level of theory.

## Graphical abstract

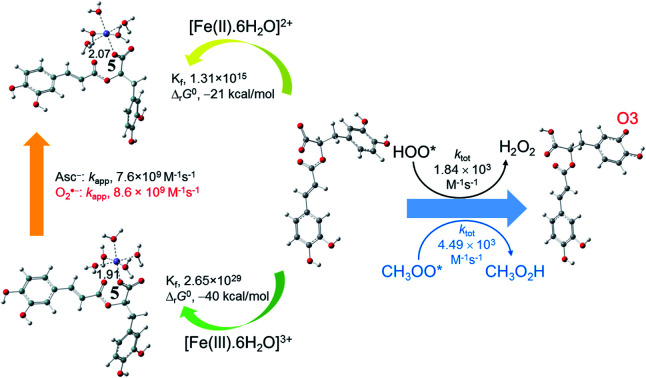

## Introduction

Oxidative stress (OS) resulting from free radical action is one of the reasons for the serious decline in human health.^1,2^ Free radicals damage biological compounds that make up human cells (*i.e.* lipid and protein) or carry genetic information (*i.e.* DNA and RNA).^1,3,4^ This causes several diseases such as cancers, heart diseases, and Alzheimer's disease.^5–11^ Many methods have been used to protect human health from OS. Among them, the use of antioxidant compound supplementary provided from natural products or diets is one of the most effective ways.^1,12^ Phenolic compounds that are ubiquitously distributed phytochemicals found in most fruits and vegetables have widely been investigated as potent antioxidants towards different free radicals, mostly peroxyl radicals including HOO˙, CH_3_OO˙, and C_2_H_5_OO˙. For example, fraxetin can scavenge HOO˙/CH_3_OO˙ with overall rate constants (*k*_overall_) of 3.99 × 10^8^ M^−1^ s^−1^/2.76 × 10^9^ M^−1^ s^−1^ and 2.43 × 10^4^ M^−1^ s^−1^/2.81 × 10^3^ M^−1^ s^−1^ in aqueous and pentyl ethanoate (PEA), respectively.^13^ Ellagic acid is also reported to be able to react with HOO˙ radicals *via* a hydrogen transfer (HT) mechanism with *k*_overall_ values of 1.57 × 10^5^ M^−1^ s^−1^ and 4.29 × 10^2^ M^−1^ s^−1^ in water and PEA media, respectively.^14^ Similarly, different phenolic compounds have also been analyzed for evaluating their scavenging activities towards peroxyl radicals: propyl gallate,^15^ esculetin,^16^*trans*-resveratrol,^17^ capsaicin,^18^ sinapinic acid,^19^ piceatannol,^20^ genistein, daidzein, glycitein, equol, 6-hydroxydaidzein, 8-hydroxiglycitein,^21^ and dihydroxybenzoic acids.^22^

The hydroxycinnamic acids, a class of polyphenol compounds, have already demonstrated their benefits to human health, including antioxidant,^9,12,23^ anticancer,^7,24^ anti-inflammatory,^25,26^ and antiviral^27^ activities. The antioxidant activity of hydroxycinnamic acids and their derivatives has attracted the attention of many researchers.^5–12,23,28^ Owing to the presence of phenolic hydroxyl groups and the participation of large conjugated systems, many hydroxycinnamic acids are expected to have high free radical scavenging activities based on the H-atom transfer from the –OH group.^5,6^ In addition, the secondary antioxidant activity *via* transition metal ion chelation of hydroxycinnamic acids has also been well reported.^9,10^ Normally, these acids contain –COOH and –OH groups and sometimes have the ester or ether groups that allow the complexation with metal ions.^9,29–31^

**Fig. 1:**
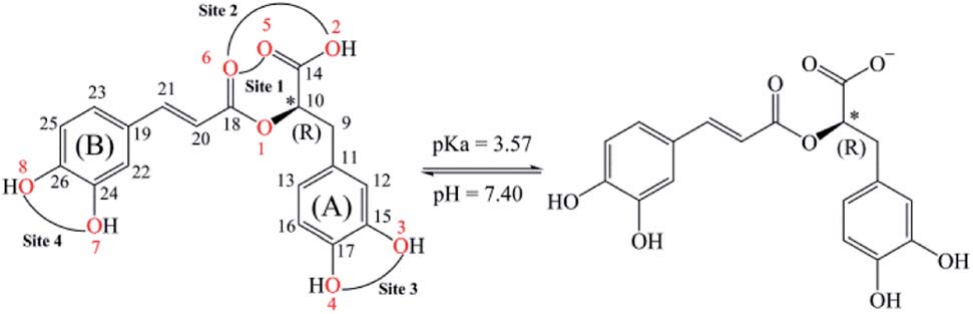
2D structures of (R)-rosmarinic acid (RA) and its mono-anion form under physiological conditions (pH = 7.40) with the numbered atoms. Four possible chelating sites on the neutral RA are also mentioned.

Rosmarinic acid (RA, Fig. 1) is one of the hydroxycinnamic acids, and was initially isolated and purified from the extract of rosemary, a member of mint family (Lamiaceae), in 1958 by Scarpati and Oriente.^32,33^ With an acid dissociation constant *K*_a_ of 10^−3^,^57^ mono-anionic RA^−^ is the main form of RA existing in the physiological environment (pH ranging from 7.35 to 7.45).^34^ Rosmarinic acid also shows a large number of biological and pharmacological activities including anti-myotoxic,^35^ antioxidant,^36–40^ anti-inflammatory,^26^ antimicrobial,^41^ anti-mutagenic,^42^ anti-cancer,^43^ antibacterial,^44^ and antiviral^27^ functions. Moreover, RA is able to interact with several high-molecular weight compounds such as proteins and lipids.^40,45–47^ In a research study by Xin Peng *et al.*, the interaction of RA with bovine serum albumin (BSA) was investigated by combining experiments and molecular docking.^46^ The experimental results indicate that BSA has a high affinity towards RA with a binding constant of 4.18 × 10^4^ mol L^−1^. Meanwhile, the docking results show that RA is bound to the site-1 (subdomain IIA) of BSA, at Leu-209, Val-239, Leu-196, Trp-212, Ala-289 and Leu-236 amino acids.

Among the biological activities of RA, its antioxidant activity has been investigated *via* both experimental and computational approaches.^36–40^ For example, Fadel *et al.* experimentally studied the antioxidant activity of RA in preventing lipid peroxidation.^36^ The authors measured the peroxidation of liposomes of 1,2-dilinoleoyl-*sn*-glycero-3-phosphocholine (DLPC) at 37 °C with a hydrophilic radical generator, namely, 2,2′-azo-bis(2-amidinopropane)dihydrochloride (AAPH). The results indicate that DLPC is fully peroxidized at a RA concentration of 0.25 μM, whereas the peroxidation level is lower than 20% for a RA concentration greater than 2 μM after 60 min. Popov *et al.* conducted a comparative study on the radical scavenging properties of RA in a system of 2,2′-azo-bis(2-methylpropionamidin)dihydrochloride/luminol and hemoglobin/hydrogen peroxide/luminol to determine its protective potential in preventing peroxidation of linoleic acid.^40^ The antioxidant activity of RA in this studied system is remarkably higher than that of trolox, ascorbic acid and taxifolin. Additionally, Cao *et al.* evaluated the antioxidant activity of RA *via* a DFT approach at the B3LYP/6-311G(d) level of theory.^37^ Their results indicated the BDE values of O7–H, O8–H, and O4–H bonds (see Fig. 1) are 325.7, 314.9, and 316.5 kJ mol^−1^, respectively. Furthermore, the radical resulting from the H-abstraction of O7–H is more stable than that of the remaining O–H bonds. Besides, Świsłocka *et al.* combined DFT calculations at the B3LYP/6-311+G(d,p) level of theory and experiments to investigate the structures and antioxidant properties of rosmarinic acid and its alkali metal salts.^48^ In this work, the antioxidant activities of RA and its lithium, sodium, and potassium salts were determined by their ability to scavenge 1,1-diphenyl-2-picrylhydrazyl (DPPH˙) radicals and to reduce the ferric complex in the ferric reducing antioxidant power (FRAP) assay. The results indicate that all of these salts have better antioxidant properties than that of initial RA. Moreover, the IC_50_ values in the DPPH˙ assay are very similar for all the studied salts, whereas sodium rosmarinate shows the highest antioxidant activity in the FRAP assay. There has been scare study on the pro-oxidant activities of rosmarinic acid in the literature. Muñoz-Muñoz *et al.* experimentally investigated the intrinsic pro-oxidant activity of RA *via* generation of H_2_O_2_ and free radicals by the action of peroxidase in competition with its antioxidant properties based on consumption of H_2_O_2_ and free radicals.^49^ In balancing between the pro-oxidant and antioxidant activities, the authors concluded that RA can be considered as a net antioxidant.

Although there are some experimental and computational works dedicated for the antioxidant and pro-oxidant activities of rosmarinic acid, systematic study of its chemical kinetics in free radical scavenging mechanisms is still lacking, and hence, further detailed studies are needed. Furthermore, the secondary antioxidant properties based on the transition metal chelating ability, and the pro-oxidant activity based on redox processes that may initiate Fenton-like reactions yielding reactive HO˙ radicals, have not been reported in the literature yet.

Thus, the main goal of this study is to evaluate the free radical scavenging activities of rosmarinic acid (RA) towards the ROO˙ radical family including HOO˙ and CH_3_OO˙ in aqueous and pentyl ethanoate (PEA) phases. Density functional theory (DFT) was used to optimize the structures and to calculate vibrational frequencies for different forms of RA including neutral, mono-anion, radicals, and metal complexes. The intrinsic thermochemical parameters including bond dissociation energy (BDE), proton affinity (PA) and ionization potential (IP) were first calculated. The standard enthalpies (Δ_r_*H*^0^) and Gibbs free energies (Δ_r_*G*^0^) of reactions between RA and HOO˙/CH_3_OO˙ at potential positions were estimated and the kinetics of these reactions were also computed using the conventional transition state theory (TST) and quantum mechanics-based tests for the overall free radical scavenging activity (QM-ORSA) method. In addition, the secondary antioxidant properties of RA based on the chelation towards Fe(iii) and Fe(ii) ions to prevent the Harber–Weiss reaction^50–52^ forming harmful radicals were investigated. Moreover, the reactions between the Fe(iii) complexes and the reducing agents, *i.e.*, ascorbate anion and superoxide anion, were studied to evaluate the pro-oxidant activity of RA. The reaction enthalpies (Δ_r_*H*^0^) and Gibbs free energies (Δ_r_*G*^0^) of these reactions were finally calculated. Hopefully, the obtained results can explain whether there is a competition between the antioxidant and pro-oxidant properties of rosmarinic acid in the studied conditions.

## Computational method

All geometry optimizations and vibrational frequency calculations were performed using Gaussian 16 Rev. A.03 package^53^ in water and pentyl ethanoate (PEA) phases at the M05-2X/6-311++G(2df,2p) level of theory. The hybrid meta exchange–correlation GGA M05-2X functional was recommended for the thermodynamic and kinetic calculation by their developers^54^ and has widely been used in the kinetics of free radical scavenging reactions.^4,55^ The structures of [Fe(H_2_O)_6_]^2+^ and [Fe(H_2_O)_6_]^3+^ were respectively employed as ferrous and ferric ion models in the aqueous phase, as recommended by several previous research studies.^9,31,56–58^ In these models, the Fe ion interacted with six water molecules *via* the Fe–O bonds in an octahedral fashion with quintet spin for Fe(ii) complexes and sextet one for Fe(iii) complexes. Four main direct antioxidant mechanisms of RA including formal hydrogen transfer (FHT), proton loss (PL), single electron transfer (SET) and radical adduct formation (RAF) were evaluated.

First, the intrinsic thermochemical parameters including bond dissociation energies (BDE), proton affinities, and adiabatic ionization potentials (IP) characterizing respectively for FHT, PL, and SET mechanisms were calculated using the following equations:

1 BDE (R–H) = *H*(R˙) + *H*(H˙) − *H*(R–H);

2 PA = *H*(R^−^) + *H*(H^+^) − *H*(RH˙^+^);

3 IP = *H*(RH˙^+^) + *H*(e^−^) − *H*(R–H);

where *H* is the enthalpy of each species at 298.15 K and 1 M. The experimentally enthalpy values of proton (H^+^) and electron (e^−^) in the gas phase were 1.4811 and 0.7519 kcal mol^−1^, respectively.^59^ In water, *H*(H^+^) and *H*(e^−^) were calculated based on the binding of a proton and an electron to water molecules (H_2_O) to form H_3_O^+^ and H_2_O^−^, respectively; this method was recommended by several previous works.^60–62^ In this framework, *H*(H^+^) and *H*(e^−^) at the M05-2X/6-311++G(2df,2p) level of theory in the aqueous phase were defined as −251.4 and −15.4 kcal mol^−1^, respectively, whereas the corresponding values in PEA are −240.5 and −8.3 kcal mol^−1^.

The peroxyl radicals including HOO˙ and CH_3_OO˙ were chosen in order to evaluate the influence of free radicals' nature on the primary antioxidant activities of RA. A large number of works reported in the literature have recommended the use of the peroxyl radicals (ROO˙) as major reaction partners for evaluating the relative scavenging activity of different compounds.^4,63–65^ These radicals have not too short half-lives, which is required for the efficient interception by phenolic compounds.^67^ The HOO˙ is the simplest of the ROO˙ radicals that is among the free radicals of biological relevance. An excess amounts of HOO˙ in a physiological environment need to be removed to retard oxidative stress.^66^ HOO˙ and CH_3_OO˙ were therefore chosen to evaluate the influence of free radicals' nature on the primary antioxidant activities of RA.

The standard Gibbs free energies of reaction (Δ_r_*G*^0^) with free radicals, HOO˙ as an example, were calculated using equations (eqn (5), (7), (9) and (11)) for four mechanisms including FHT, PL, RAF and SET as follows:

4 - FHT: R–H + HOO˙ → R˙ + HOOH;

5 Δ_r_*G*^0^_FHT_ = [*G*(R˙) + *G*(HOOH)] − [*G*(R–H) + *G*(HOO˙)];

6 - PL: R–H + HOO˙ → R^−^ + HOOH^+^˙;

7 Δ_r_*G*^0^_PL_ = [*G*(R^−^) + *G*(HOOH^+^˙)] − [*G*(R–H) + *G*(HOO˙)];

8 - RAF: R–H + HOO˙ → [RH–OOH]˙;

9 Δ_r_*G*^0^_RAF_ = *G*([RH–OOH]˙) − [*G*(R–H) + *G*(HOO˙)];

10 - SET: R–H + HOO˙ → R–H^+^˙ + HOO^−^;

11 Δ_r_*G*^0^_SET_ = [*G*(R–H^+^˙) + *G*(HOO^−^)] − [*G*(R–H) + *G*(HOO˙)];

The energy values for the CH_3_OO˙ radical scavenging reactions were similarly determined.

The rate constants (*k*) of three reactions FHT, RAF and SET, which may be in concurrence were calculated *via* the conventional transition state theory (TST) approach^68,69^ as follows:^4^12
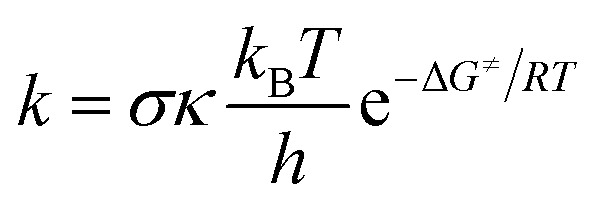
where *σ* is the reaction symmetry number or the reaction path degeneracy; *κ* is the transmission coefficient attributing for quantum tunneling effects by employing the Eckart barrier;^70^*k*_B_, *h* and *R* are the Boltzmann, Planck and molar gas constant, respectively; *T* is the temperature of the system; and Δ*G*^≠^ is the Gibbs free energy of activation. For FHT and RAF processes, Δ*G*^≠^ was calculated as the Gibbs energy difference between transition states and reactants.^4^

For the single electron transfer (SET) process, Δ*G*^≠^ was determined using the Marcus theory.^71,72^ The Δ*G*^≠^ quantity in this approach was calculated using equation (eqn (13)):13
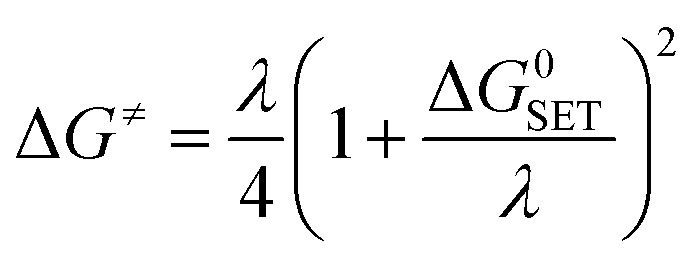
where *λ* is the nuclear reorganization energy and Δ*G*^0^_SET_ is the free energy of reaction. The value of *λ* was simply calculated by the difference in energy between Δ*G*^0^_SET_ and Δ*E*_SET_, which is the non-adiabatic energy between the reactions and products.

14 *λ* = Δ*E*_SET_ − Δ*G*^0^_SET_

In the Collins–Kimball theory,^73^ the apparent rate constant (*k*_app_) should include the diffusion limit, which is the close to or higher than the diffusion limit of the solution. The *k*_app_ value was calculated as follows:15
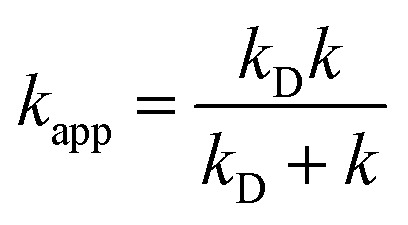
where *k* is the thermal rate constant and *k*_D_ is the steady-state Smoluchowski^74^ rate constant for an irreversible bimolecular diffusion-controlled reaction:

16 *k*_D_ = 4π*R*_AB_*D*_AB_*N*_A_

where *R*_AB_ denotes the reaction distance, *N*_A_ is the Avogadro number, and *D*_AB_ (the mutual diffusion coefficient of reactants) is estimated from *D*_A_ and *D*_B_ according to Truhlar.^75^ The values of *D*_A_ and *D*_B_ were estimated using the Stokes–Einstein approach:^76,77^17
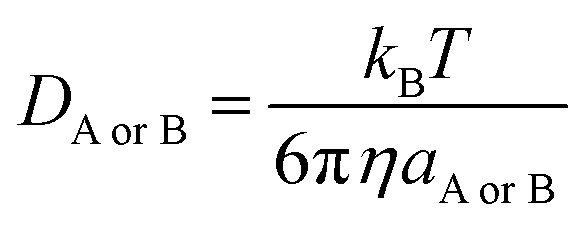
where *k*_B_ is the Boltzmann constant, *T* is the temperature, *η* denotes the viscosity of the solvent (*i.e.* the viscosity of water^15^ is 8.91 × 10^−4^ Pa s), and *a* is the radius of the solute.

When all rate constants of possible free radical scavenging reactions have been estimated, the total or overall rate coefficient (*k*_tot_)^4,78^ which characterizes the reaction rate of each antioxidant compound was calculated using equation (eqn (18)):

18 *k*_tot_ = ∑*k*^FHT^ + ∑*k*^RAF^ + ∑*k*^SET^

where *k*^FHT^, *k*^RAF^, and *k*^SET^ are the total apparent rates of the FHT, RAF and SET reactions, respectively.

Furthermore, the indirect antioxidant activity of RA was evaluated based on its chelating ability towards Fe(ii) and Fe(iii) ions. The complexation reactions of RA with [Fe(H_2_O)]^2+^ and [Fe(H_2_O)]^3+^ were estimated using reaction enthalpies (Δ_r_*H*^0^), standard Gibbs free energies (Δ_r_*G*^0^), and formation constants (*K*_f_) (eqn (19)):

19 L^*x*^ + [Fe(H_2_O)_6_]^*y*^ → [FeL(H_2_O)_6−*n*_]^*x*+*y*^ + *n*H_2_O;

where *n* = 1 or 2 corresponds to the formation of mono- or bidentate complexes. L is the RA ligand in the neutral form or mono-anionic one. *x* and *y* are the charges of RA (*x* = 0 or −1) and iron ion (*y* = +2 or +3), respectively. Based on the reaction eqn (19), the Δ_r_*H*^0^ and Δ_r_*G*^0^ values were calculated using equations (eqn (20) and (21)):

20 Δ_r_*H*^0^ = *H*([FeL(H_2_O)_6−*n*_]^*x*+*y*^) + *nH*(H_2_O) − *H*(L^*x*^) − *H*([Fe(H_2_O)_6_]^*y*^);

21 Δ_r_*G*^0^ = *G*([FeL(H_2_O)_6−*n*_]^*x*+*y*^) + *nG*(H_2_O) − *G*(L^*x*^) − *G*([Fe(H_2_O)_6_]^*y*^);

Stability constant (*K*) is the important parameter for the investigation of equilibrium in solutions. For the complexation reactions of metal ions with different ligands, this parameter commonly called the formation constant (*K*_f_) is widely studied to evaluate the concentration of each existing form of the complexes in the solution.^79–81^ The *K*_f_ values^82^ were calculated using equation (eqn (22)):22
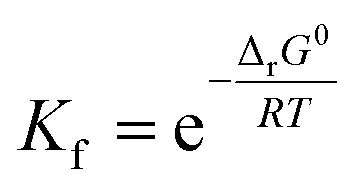


The pro-oxidant activity of RA was estimated through the reduction reactions of Fe(iii) to Fe(ii) complexes, which are involved in Fenton-like reactions producing reactive hydroxyl (HO˙) radicals. Following previous research,^9,83^ a superoxide anion radical (O_2_˙^−^) and an ascorbate anion (Asc^−^) were chosen as reductive agents. The reactions between the complexes of Fe(iii) and two reducing agents occur as follows (reactions (23) and (24)):

23 [Fe(iii)L(H_2_O)_6−*n*_]^*x*+3^ + (O_2_˙^−^) → [Fe(ii)L(H_2_O)_6−*n*_]^*x*+2^ + O_2_;

24 [Fe(iii)L(H_2_O)_6−*n*_]^*x*+3^ + (Asc^−^) → [Fe(ii)L(H_2_O)_6−*n*_]^*x*+2^ + Asc˙;

The corresponding redox reactions of aqueous complexes occur as follows (reactions (25) and (26)):

25 [Fe(iii)(H_2_O)_6_]^3+^ + (O_2_˙^−^) → [Fe(ii)(H_2_O)_6_]^2+^ + O_2_;

26 [Fe(iii)(H_2_O)_6_]^3+^ + (Asc^−^) → [Fe(ii)(H_2_O)_6_]^2+^ + Asc˙;

The standard reaction enthalpies (Δ_r_*H*^0^) and Gibbs free energies (Δ_r_*G*^0^) of the reactions (23) and (24) were also determined by the difference in the total enthalpies (*H*) and Gibbs free energy (*G*), respectively, between the products and the reactants as follows (eqn (27)–(30)):

For superoxide anion radical (O_2_˙^−^):

27 Δ_r_*H*^0^ = *H*([Fe(ii)L(H_2_O)_6−*n*_]^*x*+2^) + *H*(O_2_) − *H*([Fe(iii)L(H_2_O)_6−*n*_]^*x*+3^) − *H*(O_2_˙^−^);

28 Δ_r_*G*^0^ = *G*([Fe(ii)L(H_2_O)_6−*n*_]^*x*+2^) + *G*(O_2_) − *G*([Fe(iii)L(H_2_O)_6−*n*_]^*x*+3^) − *G*(O_2_˙^−^);

For ascorbate anion (Asc^−^):

29 Δ_r_*H*^0^ = *H*([Fe(ii)L(H_2_O)_6−*n*_]^*x*+2^) + *H*(Asc˙) − *H*([Fe(iii)L(H_2_O)_6−*n*_]^*x*+3^) − *H*(Asc^−^);

30 Δ_r_*G*^0^ = *G*([Fe(ii)L(H_2_O)_6−*n*_]^*x*+2^) + *G*(Asc˙) − *G*([Fe(iii)L(H_2_O)_6−*n*_]^*x*+3^) − *G*(Asc^−^);

The energy values for the redox reactions of aqueous complexes were similarly determined.

SEAGrid (http:www.seagrid.org)^84–87^ is acknowledged for computational resources and services for the selected results used in this publication.

## Results and discussion

### Direct antioxidant potential: intrinsic thermochemical properties

**Fig. 2:**
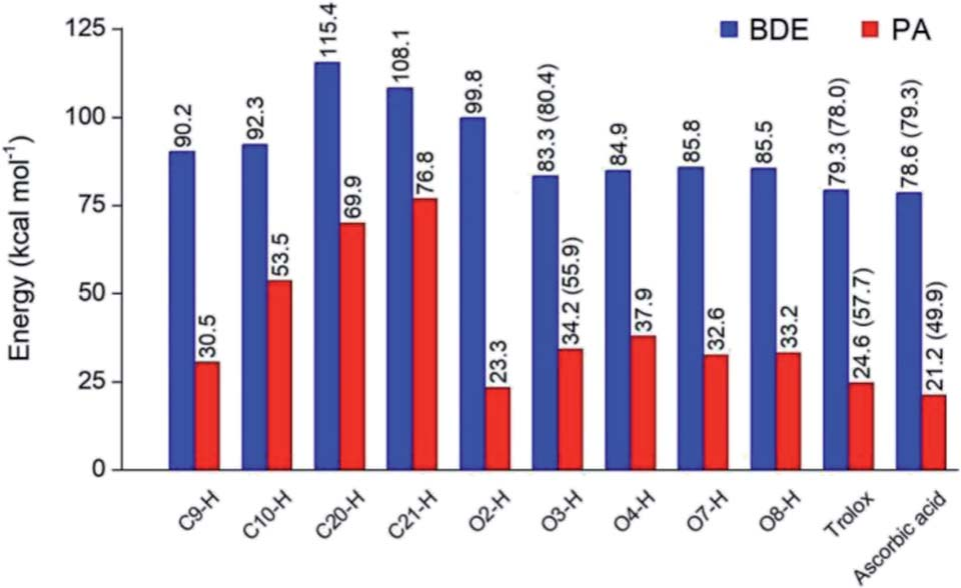
Thermochemical properties including BDE and PA values (in kcal mol^−1^) for rosmarinic acid and trolox, ascorbic acid being used as the compounds of reference in water at 298.15 K calculated at the M05-2X/6-311++G(2df,2p) level of theory. The values in parentheses correspond to the results obtained in the PEA phase.

Optimized structures of rosmarinic acid in neutral and mono-anionic forms in the water phase at 298.15 K calculated at the M05-2X/6-311++G(2df,2p) level of theory are presented in Fig. S1 (ESI file†). An intrinsic reactivity-based strategy, which only focuses on the chemical nature of the studied compound itself, is a helpful approach to quickly screen potential antioxidants. The intrinsic thermochemical parameters including BDE, PA, and IP values were first calculated to determine the antioxidant potential of RA *via* three mechanisms FHT, PL (Fig. 2), and SET (Fig. S2, ESI file†), respectively.

It can be seen in Fig. 2 that the phenolic O–H bonds (*i.e.* O3–H, O4–H, O7–H, and O8–H) have lower BDE values ranging from 83.3 to 85.8 kcal mol^−1^ than that of other bonds from 90.2 to 115.4 kcal mol^−1^. Therefore, the FHT reactions on the RA molecule may probably involve O–H sites. The two weakest O–H bonds are found at the O3–H (83.3 kcal mol^−1^) and O4–H (84.9 kcal mol^−1^) sites of the A ring (Fig. 1). These values are higher than the BDEs of trolox and ascorbic acid calculated at the same level of theory, 79.3 and 78.6 kcal mol^−1^, respectively. When compared with several other antioxidants in previous studies obtained by the same M05-2X method, the BDE value at the O3–H of RA is lower than that in water of 2-(*sec*-butyl)-7,8-dimethoxybenzo[de]imidazo[4,5,1-*ij*][1,6]-naphthyridin-10(9*H*)-one (84.9 kcal mol^−1^),^61^ xanthyletin (87.1 kcal mol^−1^),^62^*trans-p*-coumaric acid (85.0 kcal mol^−1^)^6^ and protocatechuic acid (83.9 kcal mol^−1^).^88^ Furthermore, the hydrogen dissociation in the PEA solvent is slightly more favorable than that in water with a lower BDE value, for example, a BDE (O3–H) of 80.4 kcal mol^−1^ as compared to 83.3 kcal mol^−1^ in water. This value is also slightly higher than those of trolox and ascorbic acid in the same medium.

The PA values characterize the deprotonation process of molecules involved in the first step of SPL-ET or SPL-HAT two-step mechanisms.^1^ The second step of SPL-ET mechanism is related to the electron transfer from the deprotonated antioxidant to the free radical, whereas the second transferred species in SPL-HAT is a hydrogen atom. Thus, the PA parameter allows characterizing the preponderance of these mechanisms. The lower the PA value, the higher the deprotonation potential of molecules. The PA values of different C–H and O–H bonds present in Fig. 2 vary from 23.3 to 76.8 kcal mol^−1^. The PA values of O–H bonds (from 23.3 to 37.9 kcal mol^−1^) are generally lower than the ones of C–H (from 53.5 to 76.8 kcal mol^−1^), except a quite low value of the C9–H bond (30.5 kcal mol^−1^). Furthermore, the PA value observed at the C9–H bond (30.5 kcal mol^−1^) is quite lower than that of C20–H (69.9 kcal mol^−1^) and C21–H (76.8 kcal mol^−1^). The lower PA value of the C9–H bond is due to the cleavage of the C10–O1 bond resulting from the proton dissociation at the C9–H position (Fig. S2, ESI file†). In addition, it is expected that a non-polar solvent like PEA is not favorable for transition of a charged particle like a proton. In fact, PA (C3–H) is equal to 55.9 kcal mol^−1^ that is remarkably higher than that in water (*i.e.* 34.2 kcal mol^−1^). Thus, the first step of SPL-ET or SPL-HAT mechanisms is expected to occur at the O2–H or C9–H of the RA molecule. The relatively low PA value at O2–H of the COOH group (23.3 kcal mol^−1^) probably indicates the presence of the mono-anionic form generated from the deprotonation at this position in water. This PA value is quite lower than that of piperidine[3,2-*b*]demethyl(oxy)aaptamine (60.2 kcal mol^−1^),^61^ pandanusin A (54.6 kcal mol^−1^), and 5-hydroxynoracronycin (36.5 kcal mol^−1^)^62^ (obtained at the M05-2X/6-311++G(d,p) level of theory).

The adiabatic IP value is the minimum energy required to remove an electron from a studied compound. Thus, the lower IP value represents the easier electron transferring ability and the higher antioxidant activity *via* the SET mechanism. As shown in Fig. S3 (ESI file†), the IP value in water of RA being 121.2 kcal mol^−1^ is lower than that of trolox (128.8 kcal mol^−1^), but higher than that of ascorbic acid (108.5 kcal mol^−1^). It is noteworthy that the PEA solvent is also unfavorable to the transfer of an electron particle from RA to free radical with IP in PEA (138.5 kcal mol^−1^) higher than that in water (121.2 kcal mol^−1^). In addition, RA presents an IP value in PEA higher than that of trolox (122.1 kcal mol^−1^) but lower than that of ascorbic acid (148.7 kcal mol^−1^).

### Kinetics of scavenging reactions towards HOO˙/CH_3_OO˙ radicals

Evaluating the antioxidant activities based on the thermochemical strategy allows considering the influence of the free radical nature by predicting reaction enthalpies (Δ_r_*H*^0^) and standard Gibbs free energies (Δ_r_*G*^0^) at 298.15 K. As discussed above, the RA^−^ mainly exists in a physiological environment under the mono-anionic form, RA^−^. Thus, in this section, we evaluate the standard Gibbs free energies of the FHT, PL, RAF and SET reactions of the RA^−^ towards HOO˙ and CH_3_OO˙ radicals as recommended by several previous studies^1,4^ (Table 1).

**Table 1:** Standard Gibbs free energies (Δ_r_*G*^0^, kcal mol^−1^) at 298.15 K of the FHT, PL, RAF and SET reactions for the rosmarinate mono-anion (RA^−^) towards HOO˙ and CH_3_OO˙ radicals in water at 298.15 K at the M05-2X/6-311++G(2df,2p) level of theory. Values in parentheses correspond to results obtained in PEA phase

Pos.	HOO˙				CH_3_OO˙			
	FHT	PL	RAF	SET	FHT	PL	RAF	SET
				30.8 (64.1)				32.5 (64.8)
C9H	1.2	44.4	—		2.2	48.4	—	
C10H	6.1	92.4	—		7.2	96.4	—	
C20	25.6	87.2	2.4 (−3.4)		26.7	91.2	9.8 (9.0)	
C21	18.3	96.8	9.7		19.4	100.8	10.4	
O3H	−5.0 (−5.2)	51.3	—		−3.9 (−3.5)	55.3	—	
O4H	−3.9	55.3	—		−2.8	59.2	—	
O7H	−2.5 (−3.4)	49.5 (103.6)	—		−1.4 (−1.7)	53.5 (88.8)	—	
O8H	−3.0	50.4	—		−1.9	54.4	—	
Trolox	−8.7 (−7.3)	40.7 (94.9)		19.4 (59.0)	−7.6 (−5.6)	44.7 (80.1)		21.1 (59.6)
Ascorbic acid	−9.4 (−5.8)	36.4 (86.8)		40.0 (86.1)	−8.3 (−4.1)	40.3 (72.0)		41.7 (86.8)

The Δ_r_*G*^0^ values of the FHT reaction between RA^−^ towards HOO˙ and CH_3_OO˙ at the phenolic O–H positions are all negative, and thus, the FHT reactions occurring at these positions are all favorable and spontaneous. The most favorable reactions in water are expected at the O3–H site with the Δ_r_*G*^0^ value being −5.0 and −3.9 kcal mol^−1^ for the reaction towards HOO˙ and CH_3_OO˙ radicals, respectively. Moreover, Δ_r_*G*^0^ values of these reactions are less negative than that of trolox and ascorbic acid being −8.7 and −9.4 kcal mol^−1^ for the reactions with HOO˙, and −7.6 and −8.3 kcal mol^−1^ for the ones with CH_3_OO˙, respectively. Besides, the lowest Δ_r_*G*^0^ value of FHT between RA^−^ and HOO˙ is very close to those obtained by the DFT/M05-2X method for piperidine[3,2-*b*]demethyl(oxy)aaptamine (−5.0 kcal mol^−1^),^61^ pandanusin (−5.0 kcal mol^−1^), citrusinine-I (−4.9 kcal mol^−1^),^62^ and 5-hydroxynoracronycin (−4.6 kcal mol^−1^)^62^ and remarkably lower than those of 9-amino-2-ethoxy-8-methoxy-3*H*-benzo[de][1,6]naphthyridine-3-one (7.1 kcal mol^−1^)^61^ and tryptamine (−3.8 kcal mol^−1^).^78^ In contrast, the reactions occurring at the C–H positions are all unfavorable with positive Δ_r_*G*^0^ values ranging from 1.2 to 25.6 kcal mol^−1^ for the HOO˙ radical and from 2.2 to 26.7 kcal mol^−1^ for the CH_3_OO˙ one. Furthermore, the FHT process occurs in the PEA solvent more favorably than in the aqueous phase. In fact, Δ_r_*G*^0^ values in PEA of the reactions with HOO˙ obtained at O3–H and O7–H are −5.2 and −3.4 kcal mol^−1^, which are slightly lower than those in water (*i.e.* −5.0 and −2.5 kcal mol^−1^, respectively).

Regarding the PL mechanism, it is observed that all the reactions towards both the radicals show large positive Δ_r_*G*^0^ values. The lowest Δ_r_*G*^0^ value of PL reactions was found at the C9–H bond with the values in water as 44.4 and 48.4 kcal mol^−1^ for HOO˙ and CH_3_OO˙ radicals, respectively. In addition, the Δ_r_*G*^0^ values of these reactions are higher than those of trolox (*i.e.* 40.7 kcal mol^−1^ for HOO˙ and 44.7 kcal mol^−1^ for CH_3_OO˙) and ascorbic acid (*i.e.* 36.4 kcal mol^−1^ for HOO˙ and 40.3 kcal mol^−1^ for CH_3_OO˙). As mentioned above, the PEA medium largely increases the Δ_r_*G*^0^ value of the PL process. For example, the Δ_r_*G*^0^ values obtained in PEA at O7–H (103.6 and 88.8 kcal mol^−1^) is higher than that in water (49.5 and 53.5 kcal mol^−1^ for HOO˙ and CH_3_OO˙, respectively).

The RAF reactions of RA^−^ were examined at the C20=C21 double bond. Similar to the PL reactions, the Δ_r_*G*^0^ values of the RAF reactions are all positive. It is noteworthy that the reactions of HOO˙ and CH_3_OO˙ radicals with RA^−^ at the C20 position are more favorable than those at the C21 one. Indeed, the Δ_r_*G*^0^ values of the RAF reaction at C20 are 2.4 and 9.8 kcal mol^−1^ for HOO˙ and CH_3_OO˙ radicals, respectively, whereas the ones at C21 are 9.7 and 10.4 kcal mol^−1^, in turn. In addition, the lowest Δ_r_*G*^0^ of RAF reactions between RA^−^ and HOO˙ is lower than those obtained by the DFT/M05-2X method for 9-amino-2-ethoxy-8-methoxy-3*H*-benzo[de][1,6]naphthyridine-3-one (5.2 kcal mol^−1^)^61^ and tryptamine (2.5 kcal mol^−1^).^78^ In contrast to FHT and PL, the Δ_r_*G*^0^ value of the RAF reaction in the PEA solvent is slightly lower than that in water. Indeed, Δ_r_*G*^0^ (C20) values in PEA are equal to −3.4 and 9.0 kcal mol^−1^ that are lower than those in water, which are 2.4 and 9.8 kcal mol^−1^ for HOO˙ and CH_3_OO˙ radical, respectively (Table 1).

Moreover, the SET reactions of RA^−^ toward HOO˙ and CH_3_OO˙ radicals also show positive Δ_r_*G*^0^ values of 30.8 and 32.5 kcal mol^−1^, in sequence. Additionally, the Δ_r_*G*^0^ values are comparable with the ones of trolox (*i.e.* 19.4 and 21.1 kcal mol^−1^ for HOO˙ and CH_3_OO˙ radicals, respectively) and ascorbic acid (*i.e.* 40.0 and 41.7 kcal mol^−1^ for HOO˙ and CH_3_OO˙ radicals). The non-polar PEA solvent is shown to be unfavorable for the electron transfer reaction as compared to the one in a polar solvent like water. Overall, the FHT and RAF reactions of RA^−^ toward HOO˙ and CH_3_OO˙ radicals are likely more favorable than that of the PL and SET reactions.

**Fig. 3:**
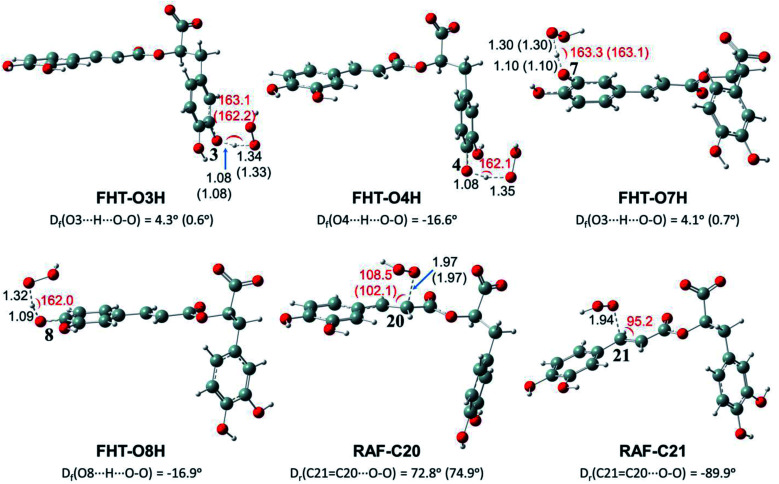
Optimized structures of the transition states (TSs) for FHT and RAF reactions of rosmarinate mono-anion (RA^−^) towards HOO˙ radicals in water calculated at the M05-2X/6-311++G(2df,2p) level of theory. *D*_f_ is the OHOO dihedral angle of the FHT TSs; *D*_r_ is the CCOO one of the RAF TSs. The values in parentheses correspond to the geometrical parameters obtained in the PEA phase.

**Fig. 4:**
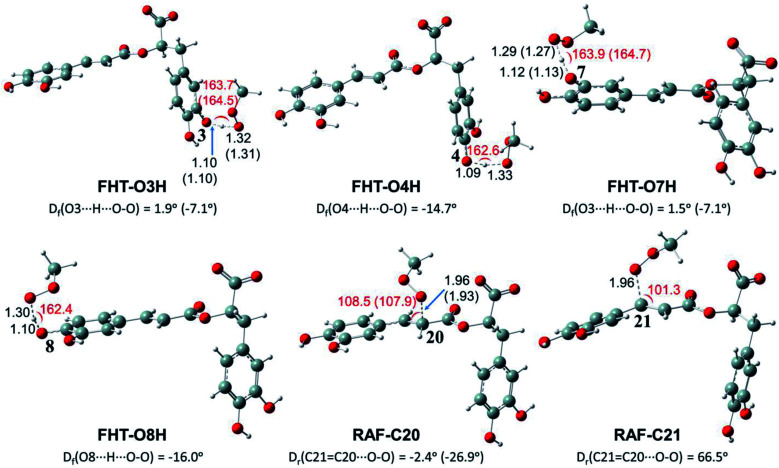
Optimized structures of the transition states (TSs) for FHT and RAF reactions of rosmarinate mono-anion (RA^−^) towards CH_3_OO˙ radicals in water calculated at the M05-2X/6-311++G(2df,2p) level of theory. *D*_f_ is the OHOO dihedral angle of the FHT TSs; *D*_r_ is the CCOO one of the RAF TSs. The values in parentheses correspond to the geometrical parameters obtained in the PEA phase.

Depending on the chemical structure of the potential antioxidant, the RAF reaction may be in competition with the FHT one.^1,61,62,78^ In this work, the kinetic calculations were considered for the FHT reactions at the O–H sites showing negative Δ_r_*G*^0^ values, also for all RAF and SET reactions. Optimized structures of the transition states (TSs) for FHT and RAF reactions of RA^−^ toward HOO˙ and CH_3_OO˙ radicals in water are presented in Fig. 3 and 4, respectively. The corresponding Cartesian coordinates and thermochemistry data are also presented in Tables S1 and S2 (ESI file†). The similar data for the reactions occurring in pentyl ethanoate (PEA) that mimics lipid media are also resumed in Table S3 (ESI file†).

As can be seen in Fig. 3 and 4, the O–H bond lengths at the TSs of FHT vary from 1.08 to 1.09 Å and from 1.10 to 1.18 Å for HOO˙ and CH_3_OO˙ radicals, respectively. Meanwhile, the distances between the shifting H-atom and the reacted O-atom of the radical are noticeably longer ranging from 1.30 to 1.35 Å for HOO˙ and from 1.29 to 1.33 Å for CH_3_OO˙. The bent angles for H⋯O⋯H in FHT reactions vary from 162.0 to 164.0°. For RAF reactions, the bond distances between the oxygen atom of the radical and the C-sp^2^ atom range from 1.94 to 1.97 Å, whereas the interactive O⋯C=C angles change from 95.2 to 108.5°.

In order to evaluate the kinetics of HOO˙ and CH_3_OO˙ scavenging reactions for RA^−^, the Gibbs free energy of activation (Δ*G*^≠^), diffusion rate constant (*k*_D_), TST thermal rate constant (*k*_T_), Eckart-tunneling-corrected rate constants (*k*_eck_), diffusion-corrected apparent rate constants (*k*_app_), and branching ratio (*Γ*) for FHT, RAF and SET reactions were calculated at 298.15 K in water and PEA using conventional transition state theory (TST). The kinetics data are shown in Table 2 for the reactions of HOO˙ radicals and Table 3 for the reactions of CH_3_OO˙ radicals.

**Table 2:** Gibbs free energy of activation (Δ*G*^≠^, kcal mol^−1^), diffusion rate constant (*k*_D_, M^−1^ s^−1^), TST thermal rate constant (*k*_T_, M^−1^ s^−1^), Eckart-tunneling-corrected rate constants (*k*_eck_, M^−1^ s^−1^), diffusion-corrected apparent rate constants (*k*_app_, M^−1^ s^−1^) and branching ratio *Γ* (%) at 298.15 K for the FHT, RAF and SET reactions of the rosmarinate mono-anion (RA^−^) with HOO˙ radicals in water calculated at the M05-2X/6-311++G(2df,2p) level of theory. The values in parentheses correspond to the results obtained in the PEA phase

Position	Δ*G*^≠^	*k*_D_	*k*_T_	*k*_eck_	*k*_app_	*Γ*
**FHT**						
O3H	18.6 (17.1)	2.41 × 10^9^ (2.55 × 10^9^)	4.73 × 10^2^ (8.25 × 10^3^)	6.44 × 10^4^ (1.39 × 10^5^)	4.73 × 10^2^ (8.25 × 10^3^)	25.70
O4H	18.8	2.41 × 10^9^	3.44 × 10^2^	4.26 × 10^4^	3.44 × 10^2^	18.67
O7H	18.8 (17.6)	2.39 × 10^9^ (2.53 × 10^9^)	8.73 × 10^2^ (7.05 × 10^3^)	2.72 × 10^5^ (2.58 × 10^5^)	8.73 × 10^2^ (7.05 × 10^3^)	47.41
O8H	20.2	2.40 × 10^9^	1.51 × 10^2^	8.44 × 10^4^	1.51 × 10^2^	8.21

**RAF**						
C20	21.4 (23.8)	1.98 × 10^9^ (2.10 × 10^9^)	9.76 × 10^−2^ (8.01 × 10^−3^)	1.36 × 10^−1^ (1.12 × 10^−2^)	9.76 × 10^−2^ (8.01 × 10^−3^)	0.01
C21	23.7	1.95 × 10^9^	2.65 × 10^−3^	4.42 × 10^−3^	2.65 × 10^−3^	0.00

**SET**						
	36.1 (103.1)	8.36 × 10^9^ (8.93 × 10^9^)	5.03 × 10^−13^ (4.10 × 10^−62^)	—	5.03 × 10^−13^ (4.41 × 10^−62^)	0.00

**Table 3:** Gibbs free energy of activation (Δ*G*^≠^, kcal mol^−1^), diffusion rate constant (*k*_D_, M^−1^ s^−1^), TST thermal rate constant (*k*_T_, M^−1^ s^−1^), Eckart-tunneling-corrected rate constants (*k*_eck_, M^−1^ s^−1^), diffusion-corrected apparent rate constants (*k*_app_, M^−1^ s^−1^) and branching ratio *Γ* (%) at 298.15 K for the FHT, RAF and SET mechanism of the rosmarinate mono-anion (RA^−^) with CH_3_OO˙ radicals in water calculated at the M05-2X/6-311++G(2df,2p) level of theory. The values in parentheses correspond to the results obtained in the PEA phase

Position	Δ*G*^≠^	*k*_D_	*k*_T_	*k*_eck_	*k*_app_	*Γ*
**FHT**						
O3H	19.1 (18.0)	2.41 × 10^9^ (2.36 × 10^9^)	3.52 × 10^2^ (3.74 × 10^3^)	8.10 × 10^4^ (1.34 × 10^5^)	3.52 × 10^2^ (3.74 × 10^3^)	7.84
O4H	18.6	2.40 × 10^9^	8.61 × 10^2^	2.07 × 10^5^	8.61 × 10^2^	19.17
O7H	18.3 (19.0)	2.39 × 10^9^ (2.35 × 10^9^)	3.22 × 10^3^ (1.73 × 10^3^)	1.72 × 10^6^ (1.65 × 10^5^)	3.22 × 10^3^ (1.73 × 10^3^)	71.60
O8H	21.1	2.40 × 10^9^	6.20 × 10^1^	7.07 × 10^4^	6.20 × 10^1^	1.38

**RAF**						
C20	22.3 (25.9)	1.97 × 10^9^ (1.94 × 10^9^)	2.39 × 10^−2^ (2.70 × 10^−4^)	3.42 × 10^−2^ (4.24 × 10^−4^)	2.39 × 10^−2^ (2.7 × 10^−4^)	0.00
C21	24.4	1.92 × 10^9^	8.79 × 10^−4^	1.65 × 10^−3^	8.79 × 10^−4^	0.00

**SET**						
	40.4 (104.5)	7.88 × 10^9^ (8.59 × 10^9^)	3.41 × 10^−16^ (3.77 × 10^−63^)	—	3.41 × 10^−16^ (3.77 × 10^−63^)	0.00

As observed in Table 2, FHT reactions demonstrate lower activation energies (*i.e.* from 18.6 for O3–H to 20.2 kcal mol^−1^ for O8–H) than those of RAF reactions (*i.e.* 21.4 and 23.7 kcal mol^−1^ for C20 and C21 positions, respectively) as well as than that of the SET reaction (*i.e.* 36.1 kcal mol^−1^). As a result, FHT reactions have high diffusion-corrected apparent rate constants (*k*_app_), and thus, the products of HOO˙ radical scavenging of RA^−^ almost result from these reactions with a total branching ratio (*Γ*) of 99.99%. It is noteworthy that the FHT reaction occurring at O7–H is the fastest with a *k*_app_ value of 8.73 × 10^2^ M^−1^ s^−1^ and it accounts for the highest *Γ* value of 47.41%. In contrast, RAF and SET reactions occur with very low rate constants; especially, the *k*_app_ value of the SET reaction is only 5.03 × 10^−13^ M^−1^ s^−1^ and, therefore, this reaction hardly contributes to HOO˙ radical scavenging of RA^−^. Regarding two RAF reactions, the one occurring at the C20 position with a *k*_app_ value of 9.76 × 10^−2^ M^−1^ s^−1^ is more dominant than that at the C21 one showing *k*_app_ of 2.65 × 10^−3^ M^−1^ s^−1^.

Furthermore, the overall rate coefficient, *k*_tot_, for radical scavenging of RA^−^ towards HOO˙ is 1.84 × 10^3^ M^−1^ s^−1^, which is close to those obtained by the DFT/M05-2X method for guaiacol (1.55 × 10^3^ M^−1^ s^−1^),^89^ 1-methyluric acid (1.08 × 10^3^ M^−1^ s^−1^)^90^ and about 100 times higher than those of vanillin (9.75 × 10^1^ M^−1^ s^−1^),^89^ caffeine (3.19 × 10^1^ M^−1^ s^−1^),^91^ and *N*(1)-acetyl-*N*(2)-formyl-5-methoxykynuramine (4.57 × 10^1^ M^−1^ s^−1^).^92^

Regarding the CH_3_OO˙ scavenging reactions, the FHT mechanism is also the main process in forming approximately 100% product. The Gibbs free energies of activation (Δ*G*^≠^) vary from 18.3 to 21.1 kcal mol^−1^, while the ones of RAF are 22.3 and 24.4 kcal mol^−1^ for the reactions at the C20 and C21 positions, respectively. The Δ*G*^≠^ value of SET is also the highest one being 40.4 kcal mol^−1^. Besides, the most potential position for the FHT process is found at the –O7H position with a *k*_app_ value of 3.22 × 10^3^ M^−1^ s^−1^ and *Γ* value of 71.60%. The RAF reactions at the C20 and C21 positions show lower *k*_app_ values (*i.e.* 2.39 × 10^−2^, 8.79 × 10^−4^, respectively) than the ones of FHT reactions. The SET reaction is also negligible with a *k*_app_ value of 3.41 × 10^−16^ M^−1^ s^−1^. The CH_3_OO˙ radical scavenging of RA^−^ has *k*_tot_ of 4.49 × 10^3^ M^−1^ s^−1^. Therefore, the CH_3_OO˙ radical scavenging reaction by RA^−^ is slightly more favorable and spontaneous than that of HOO˙ (*i.e.* 1.84 × 10^3^ M^−1^ s^−1^).

It is noteworthy that the PEA solvent shows different influences on the scavenging processes. In fact, the FHT reactions towards both HOO˙ and CH_3_OO˙ radicals are favored in the PEA solvent with lower Δ*G*^≠^ and *k*_app_ values, whereas the RAF and especially SET processes are all less favorable than those that occurred in the aqueous phase (Tables 2 and 3). The differences in reactivity of RA^−^ with HOO˙ and CH_3_OO˙ radicals *via* FHT and RAF reactions can be explained by different dipole moment values at their transition states (Table S4, ESI file†). As observed in Table S4,† the higher the dipole moment value of TS, the lower the Gibbs free energy of activation value, and thus the more favorable the reaction occurs.

Overall, the FHT reactions of RA^−^ towards both HOO˙ and CH_3_OO˙ radicals are more preponderant than those of RAF and SET reactions. The non-polar PEA solvent slightly enhances FHT reactions, while it is unfavorable to RAF and especially to SET reactions.

### Chemical nature of formal hydrogen transfer (FHT) reactions

**Fig. 5:**
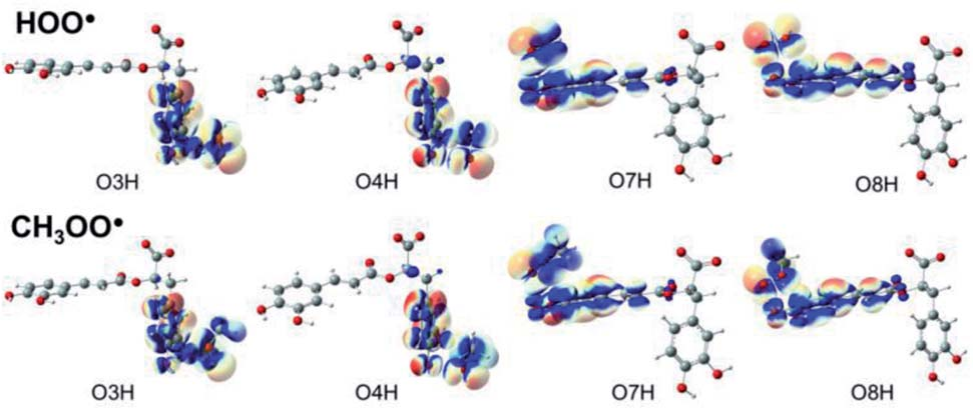
SOMO distributions of the transition states (TSs) for FHT reactions between RA^−^ with HOO˙ and CH_3_OO˙ radicals in the aqueous phase.

Understanding the chemical nature of the formal hydrogen transfer (FHT) process plays an important role in the potential applications of RA in chemical and biological fields. The FHT may occur *via* two different pathways, *i.e.* hydrogen atom transfer (HAT) or proton-coupled electron transfer (PCET), which have similar initial reactants and final products. For that reason, distinguishing these two processes is always a challenging task that needs multiple supplementary calculations and analyses, *e.g.* single occupied molecular orbital (SOMO) distributions (Fig. 5), natural bond orbital (NBO) analyses (Table S5, ESI file†), natural population analysis (NPA) charges, atomic spin densities (ASD), and natural electron configuration (NEC) at the transition states (TSs) of FHT reactions (Table S6, ESI file†).

Fig. 5 represents the SOMO distributions at the TSs for FHT reactions with HOO˙ and CH_3_OO˙ radicals at all four hydroxyl groups of RA^−^. Generally, the SOMO of HAT TSs is distributed along the H transition vector between the donor and the acceptor, whereas the one of PCET TSs is orthogonal to the transition vector.^61,93^ As can be seen in Fig. 5, the 2p orbitals of the acceptor (O atoms of hydroxyl group) and the ones of the donor (O atoms of the radicals) are interacted and distributed along the H-shifting vector. This observation is the first signal of the HAT process.

To provide more insights into the electron density interaction at the TSs of FHT reactions, we also performed the NBO analyses (Table S5, ESI file†). It is generally noted that the electron densities are essentially transferred from the lone pairs of the reactive oxygen atom on HOO˙ or CH_3_OO˙, *i.e.*, O41 or O42, to the first unoccupied anti-bonding orbitals of the shifted H from the –OH groups. The stabilization energies of these processes vary from 66.0 to 79.0 kcal mol^−1^ and from 71.2 to 85.8 kcal mol^−1^ for the reactions with HOO˙ and CH_3_OO˙ radicals, respectively. In the reverse trend, the electron densities are also donated from the electron lone pairs on the reactive oxygen atom of the studied radicals to the first unoccupied anti-bonding orbital of the shifted-H.

Furthermore, NPA charge and 1s occupancy of the mitigating H and two involved O atoms at the TSs of the FHT reactions are presented in Table S6 (ESI file†). The results indicate that the NPA charges of the mitigating H are all positive varying from 0.37 to 0.38 e^−^. These charges are similar to that reported in the literature for the HAT mechanism (*i.e.* 0.31 to 0.4 e^−^).^94^ Meanwhile, the NPA charges of two oxygen atoms involved in the FHT reaction are all negative ranging from −0.42 to −0.44 e^−^ and from −0.47 to −0.56 e^−^ for the reactive O atom of the radical and for the one of RA^−^, respectively. Moreover, the 1s occupancy show that the mitigating H is characterized by 1s^0.51–0.52^ orbital configuration, which corresponds to one H atom with the 1s^1.0^ configuration. In addition, the spin densities are all located at the H-donor, (*i.e.* 0.12286–0.14291) and -acceptor atoms (*i.e.* 0.28294–0.34933); thus, the ones at the mitigating-H are slightly negative (*i.e.* about −0.02). All the above-mentioned signals allow confirming that the shifting-H represents an atom-like species rather than a proton-like species. It means that the FHT processes at all four –OH groups have the chemical nature of the HAT mechanism.

### Preventive antioxidant potential based on iron ion-chelating activities

The secondary antioxidant activities of rosmarinic acid based on its ferrous and ferric ion chelation in preventing the formation of the reactive hydroxyl radical (HO˙) *via* the Haber–Weiss reaction^1,95,96^ were evaluated. The hydrated Fe(ii) and Fe(iii) ions existed in the octahedral-coordinated structures with six water molecules, as largely proposed in the literature^9,82,97,98^ in which the Fe(ii) or Fe(iii) ion is located at the center and H_2_O in the corner.

**Fig. 6:**
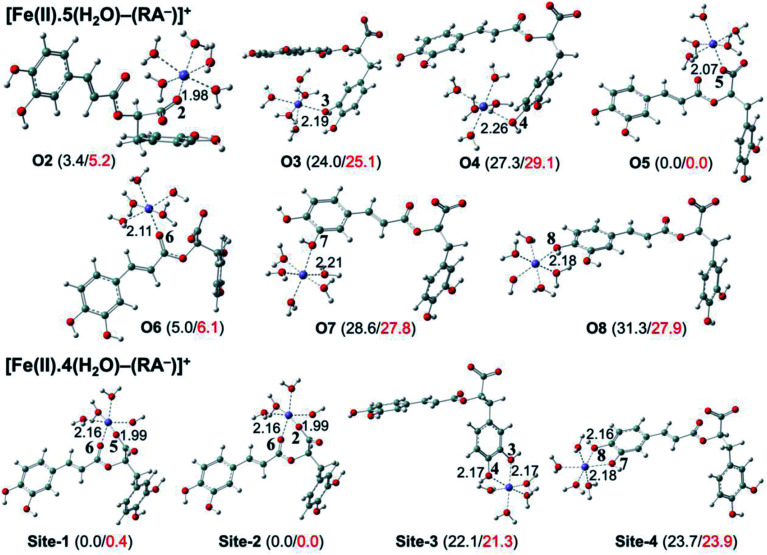
Optimized structures of 7 monodentate complex types and 4 bidentate ones between the rosmarinate mono-anion (RA^−^) and the [Fe(ii)(H_2_O)6]^2+^ ion in water. The numbers in parentheses are the relative values for standard enthalpies (in black) and Gibbs free energies (in red) (in kcal mol^−1^) of Fe(iii) complexes at 298.15 K.

**Fig. 7:**
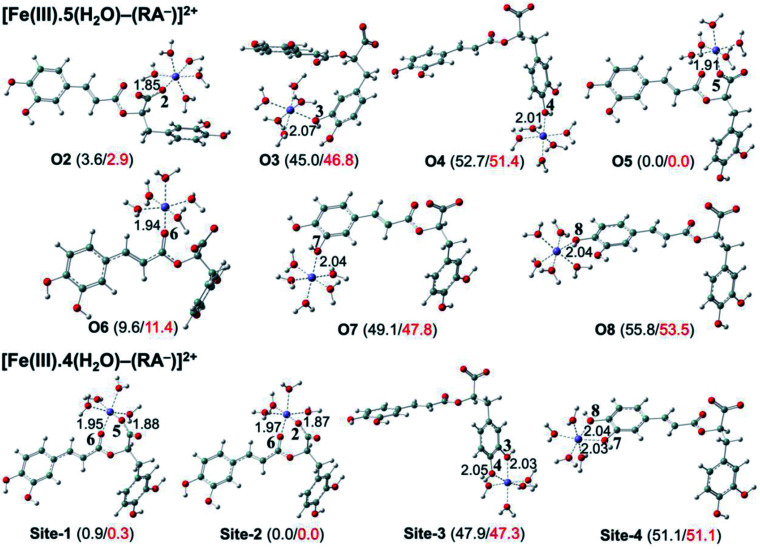
Optimized structures of 7 monodentate complex types and 4 bidentate ones between the rosmarinate mono-anion (RA^−^) and the [Fe(iii)(H_2_O)_6_]^3+^ ion in water calculated. The numbers in parentheses are the relative values for reaction enthalpies (in black) and standard Gibbs free energies (in red) (in kcal mol^−1^) of Fe(iii) complexes at 298.15 K.

The optimized structures and the relative energies of complexes between RA^−^ and [Fe(ii)(H_2_O)_6_]^3+^ and [Fe(iii)(H_2_O)_6_]^3+^ ions are shown in Fig. 6 and 7, respectively. Their Cartesian coordinates and thermochemistry data are resumed in Tables S7 and S8 (ESI file†). Table 4 presents the reaction enthalpies (Δ_r_*H*^0^), standard Gibbs free energies (Δ_r_*G*^0^), and formation constants (*K*_f_) of the chelating reactions for RA^−^ towards the hydrated Fe(ii) and Fe(iii) ions at 298.15 K. The similar data for the complexation processes between the neutral RA and the [Fe(ii)(H_2_O)_6_]^2+^ and [Fe(iii)(H_2_O)_6_]^3+^ ions are shown in Tables S9 and S10 (ESI file†), respectively. Since the mono-anionic form RA^−^ is the main existing form of rosmarinic acid in a physiological medium; thus, only the data related to the RA^−^ are presented in this section. Table S11 (ESI file†) resumes Cartesian coordinates and thermochemistry properties of ascorbate mono-anions, ascorbate radicals, superoxide anion radicals, oxygen molecules, neutral rosmarinic, mono-anion rosmarinate and aqueous iron complexes in water.

**Table 4:** Reaction enthalpies (Δ_r_*H*^0^), standard Gibbs free energies (Δ_r_*G*^0^) and formation constants (*K*_f_) of complexation reactions between the rosmarinate mono-anion (RA^−^) and [Fe(ii)(H_2_O)_6_]^2+^ and [Fe(iii)(H_2_O)_6_]^3+^ ions in water at 298.15 K. The unit of distances is Å; the units of Δ_r_*H*^0^ and Δ_r_*G*^0^ are kcal mol^−1^

Chelating position	Fe(ii) complexes			Fe(iii) complexes		
	Δ_r_*H*^0^	Δ_r_*G*^0^	*K*_f_	Δ_r_*H*^0^	Δ_r_*G*^0^	*K*_f_
O2	−24.2	−14.6	5.32 × 10^10^	−42.4	−37.1	1.57 × 10^27^
O3	−4.3	4.6	4.44 × 10^−4^	−1.1	6.6	1.34 × 10^−5^
O4	−0.6	8.9	2.85 × 10^−7^	7.1	11.8	2.27 × 10^−9^
O5	−28.4	−20.6	1.31 × 10^15^	−46.1	−40.1	2.65 × 10^29^
O6	−24.1	−15.3	1.64 × 10^11^	−37.1	−29.3	3.00 × 10^21^
O7	−0.5	6.4	1.90 × 10^−5^	3.0	7.6	2.65 × 10^−6^
O8	2.0	6.4	1.96 × 10^−5^	10.1	13.8	8.04 × 10^−11^
Site-1	−16.3	−21.7	8.12 × 10^15^	−33.1	−38.3	1.23 × 10^28^
Site-2	−16.3	−22.1	1.61 × 10^16^	−33.6	−38.4	1.29 × 10^28^
Site-3	5.3	−1.1	6.71 × 10^0^	13.7	8.4	7.34 × 10^−7^
Site-4	6.9	1.4	9.88 × 10^−2^	16.9	12.3	1.04 × 10^−9^

As can be seen in Fig. 6 and 7, the RA^−^ can chelate [Fe(ii)(H_2_O)_6_]^2+^ and [Fe(iii)(H_2_O)_6_]^3+^ ions *via* 7 oxygen atom positions including O2, O3, O4, O5, O6, O7, and O8 to form mono-dentate complexes. Besides, the bi-dentate complexes are formed at the two nearby oxygen atom positions, including site-1 (O5 and O6), site-2 (O2 and O6), site-3 (O3 and O4), and site-4 (O7 and O8) (Fig. 1). Regarding the Fe(ii) complexes, the Fe–O distances of the mono-dentate complexes vary from 1.98 to 2.26 Å, while the ones of the bi-dentate complexes vary from 1.91 to 2.18 Å (Fig. 6). In the case of Fe(iii) complexes, the Fe–O lengths vary from 1.84 to 2.06 Å for the mono-dentate complexes and from 1.88 to 2.05 Å for the bidentate ones (Fig. 7). These bond lengths are slightly shorter than those of Fe(ii)–RA^−^ complexes.

Furthermore, it is noteworthy that RA^−^ represents strong chelating ability towards both Fe(ii) and Fe(iii) ions compared to its neutral form RA (see Table S12, ESI file†). Indeed, Table 4 shows that the mono-dentate complexes formed at the O5 position have large negative Δ_r_*G*^0^ values of −20.6 and −40.1 kcal mol^−1^ for the Fe(ii) and Fe(iii) complexes, respectively. Therefore, the complexation reactions of RA^−^ with the hydrated iron ions at this site have the highest formation constants *K*_f_ of 1.31 × 10^15^ and 2.65 × 10^29^, respectively. Besides, the mono-dentate complexes formed at the O2 and O6 positions also show large negative Δ_r_*G*^0^ of −14.6/−15.3 kcal mol^−1^ for the Fe(ii) complexes, and −37.1/−29.3 kcal mol^−1^ for the Fe(iii) ones, respectively. Their *K*_f_ values vary from 5.32 × 10^10^ to 1.57 × 10^27^, respectively. Thus, the mono-dentate complexes of RA^−^ at the O2, O5, and O6 positions are all significantly favorable and exergonic, and hence, they account for high formation constants compared to other forms.

Regarding the bi-dentate complexes, all the complexes formed at the site-1 and site-2 positions have significantly large negative Δ_r_*G*^0^ values for the [Fe(ii)(H_2_O)_6_]^2+^ ion (*i.e.* −16.3 kcal mol^−1^ for both) and the [Fe(iii)(H_2_O)_6_]^3+^ ion (*i.e.* −38.3 and −38.4 kcal mol^−1^, respectively) (Table 4). These complexes are also favorable and spontaneous with high *K*_f_ values ranging from 8.12 × 10^15^ to 1.29 × 10^28^; thus, they are abundant in an aqueous environment. In contrast, the other bi-dentate complexes of RA^−^ are unstable with positive Δ_r_*G*^0^ and low *K*_f_ values.

Moreover, the chelating ability of RA^−^ towards the [Fe(iii)(H_2_O)_6_]^3+^ ion is better than the one towards the [Fe(ii)(H_2_O)_6_]^2+^ ion. For example, the Δ_r_*G*^0^ and *K*_f_ values for Fe(iii) complexes at the site-2 position are equal to −38.4 kcal mol^−1^ and 1.29 × 10^28^, respectively, which are higher than those for Fe(ii) complexes being −22.1 kcal mol^−1^ and 1.61 × 10^16^, respectively.

Overall, since the mono-anion form RA^−^ is the main existing form of rosmarinic acid under physiological conditions, RA^−^ plays the main role in the secondary antioxidant activity of rosmarinic acid *via* its chelation towards the iron ions. The most favorable chelating sites of RA^−^ constituted the O2, O5, O6, site-1, and site-2 positions. The coordination of RA^−^ with the [Fe(iii)(H_2_O)_6_]^3+^ ion is likely to be more favorable and stable than the one with [Fe(ii)(H_2_O)_6_]^2+^.

### Pro-oxidant activities of rosmarinic acid

The reduction reactions of Fe(iii)-to-Fe(ii) complexes involved in Fenton-like reactions that produce reactive hydroxyl (HO˙) radical^9,83^ are used to estimate the pro-oxidant risks of rosmarinic acid. The used reducing agents are ascorbate anion (Asc^−^) and superoxide anion (O_2_˙^−^).

Tables 5 and 6 represent the reaction enthalpies (Δ_r_*H*^0^) and standard Gibbs free energies (Δ_r_*G*^0^) of Fe(iii)-to-Fe(ii) reactions and mono-anion rosmarinate (RA^−^) complexes by Asc^−^ and O_2_˙^−^, respectively. The similar data of reduction processes of Fe(iii)-to-Fe(ii) complexes by two reducing agents for the iron complexes of neutral-rosmarinic form (RA) are shown in Table S13 (ESI file†).

**Table 5:** Standard enthalpy (Δ_r_*H*^0^), Gibbs free energy (Δ_r_*G*^0^), reorganization energy (*λ*), Gibbs free energy of activation (Δ*G*^≠^, kcal mol^−1^), diffusion rate constant (*k*_D_, M^−1^ s^−1^), TST thermal rate constant (*k*_T_, M^−1^ s^−1^), and diffusion-corrected apparent rate constants (*k*_app_, M^−1^ s^−1^) calculated at 298.15 K for the redox reaction between the superoxide anion (O_2_˙^−^) and the iron complexes in water

Position	Δ_r_*H*^0^	Δ_r_*G*^0^	*λ*	Δ*G*^≠^	*k*_D_	*k*_T_	*k*_app_
[Fe(iii)(H_2_O)_6_]^3+^ + O_2_˙^−^ → [Fe(ii)(H_2_O)_6_]^2+^ + O_2_ (eqn (25))							
	−38.2	−41.3	27.3	1.8	7.63 × 10^9^	7.30 × 10^12^	7.63 × 10^9^

[Fe(iii)L(H_2_O)_6−*n*_]^*x*+3^ + O_2_˙^−^ → [Fe(ii)L(H_2_O)_6−*n*_]^*x*+2^ + O_2_ (eqn (23))							
O2	−20.4	−19.2	26.8	0.5	8.47 × 10^9^	6.12 × 10^13^	8.47 × 10^9^
O3	−41.2	−43.2	27.0	2.4	8.54 × 10^9^	2.46 × 10^12^	8.51 × 10^9^
O4	−45.7	−43.9	25.6	3.3	8.43 × 10^9^	6.25 × 10^11^	8.43 × 10^9^
O5	−20.2	−21.4	24.5	0.1	8.58 × 10^9^	1.31 × 10^14^	8.58 × 10^9^
O6	−24.8	−26.9	28.6	0.0	8.56 × 10^9^	1.45 × 10^14^	8.56 × 10^9^
O7	−42.0	−42.8	25.6	2.5	8.51 × 10^9^	2.39 × 10^12^	8.47 × 10^9^
O8	−46.6	−47.1	24.4	5.3	8.64 × 10^9^	2.15 × 10^10^	6.16 × 10^9^
Site-1	−20.5	−23.7	27.1	0.1	8.54 × 10^9^	1.45 × 10^14^	8.54 × 10^9^
Site-2	−18.8	−18.3	22.3	0.2	8.50 × 10^9^	1.12 × 10^14^	8.50 × 10^9^
Site-3	−45.5	−49.7	25.1	6.0	8.62 × 10^9^	5.67 × 10^9^	3.42 × 10^9^
Site-4	−47.1	−51.0	25.3	6.6	8.43 × 10^9^	2.35 × 10^9^	1.84 × 10^9^

**Table 6:** Reorganization energy (*λ*), Gibbs free energy of activation (Δ*G*^≠^, kcal mol^−1^), diffusion rate constant (*k*_D_, M^−1^ s^−1^), TST thermal rate constant (*k*_T_, M^−1^ s^−1^), and diffusion-corrected apparent rate constants (*k*_app_, M^−1^ s^−1^) at 298.15 K for the reducing oxidation reaction between the ascorbate anion (Asc^−^) and the iron complexes in water

Position	Δ_r_*H*^0^	Δ_r_*G*^0^	*λ*	Δ*G*^≠^	*k*_D_	*k*_T_	*k*_app_
[Fe(iii)(H_2_O)_6_]^3+^ + Asc^−^ → [Fe(ii)(H_2_O)_6_]^2+^ + Asc˙ (eqn (26))							
	−39.3	−43.5	24.5	1.1	7.44 × 10^9^	2.22 × 10^13^	7.44 × 10^9^

[Fe(iii)L(H_2_O)_6−*n*_]^*x*+3^ + Asc^−^ → [Fe(ii)L(H_2_O)_6−*n*_]^*x*+2^ + Asc˙ (eqn (24))							
O2	−21.4	−21.3	24.0	0.5	7.58 × 10^9^	1.35 × 10^13^	7.57 × 10^9^
O3	−42.2	−45.4	24.2	4.7	7.60 × 10^9^	5.77 × 10^10^	6.72 × 10^9^
O4	−46.7	−46.1	22.8	5.9	7.60 × 10^9^	6.84 × 10^9^	3.60 × 10^9^
O5	−21.3	−23.7	21.7	0.0	7.62 × 10^9^	1.4 × 10^14^	7.62 × 10^9^
O6	−25.8	−29.1	25.8	0.1	7.61 × 10^9^	1.28 × 10^14^	7.61 × 10^9^
O7	−41.7	−43.6	22.8	4.8	7.59 × 10^9^	4.78 × 10^10^	6.55 × 10^9^
O8	−45.7	−49.3	21.6	8.8	7.64 × 10^9^	5.04 × 10^7^	5.00 × 10^7^
Site-1	−21.6	−25.9	24.3	0.0	7.60 × 10^9^	1.45 × 10^14^	7.60 × 10^9^
Site-2	−19.8	−20.5	19.5	0.0	7.58 × 10^9^	1.49 × 10^14^	7.58 × 10^9^
Site-3	−46.5	−51.9	22.3	9.8	7.63 × 10^9^	9.23 × 10^6^	9.22 × 10^6^
Site-4	−48.1	−53.2	22.5	10.5	7.56 × 10^9^	2.93 × 10^6^	2.92 × 10^6^

It can be seen that all the reaction enthalpies (Δ_r_*H*^0^) and standard Gibbs free energies of reactions (Δ_r_*G*^0^) for the reduction process of the Fe(iii) to Fe(ii) complexes by both Asc^−^ (Table 5) and O_2_˙^−^ (Table 6) are largely negative; thus, these reactions are spontaneous and exergonic. Especially, the reactions between Asc^−^ and O_2_˙^−^ with mono-dentate complexes at the O8 position and with the bi-dentate complexes at the site-3, site-4 have significantly negative Δ_r_*G*^0^ values being −49.3 and −47.1 kcal mol^−1^, −51.9 and −49.7 kcal mol^−1^, and −53.2 and −51.0 kcal mol^−1^, respectively. These values are noticeably lower than those of the similar reaction for [Fe(iii)(H_2_O)_6_]^3+^ to [Fe(ii)(H_2_O)_6_]^2+^ complexes (*i.e.* −43.5 and −41.3 kcal mol^−1^, respectively). Furthermore, the Δ_r_*G*^0^ value of the reduction process between the mono-dentate complexes with the two studied reducing agents at O3, O4, and O7 are all lower than those of [Fe(iii)(H_2_O)_6_]^3+^ complexes. Therefore, these complexes are expected to have risk to promote the Fenton reactions. However, it is noted that all three mono-dentate Fe(iii) complexes have low *K*_f_ values (Table 4); thus, their formation in aqueous solutions can be considered to be negligible, and their pro-oxidant risk is limited.

On the contrary, the main existing complexes, including the ones at the O2, O5, O6, site-1, and site-2 positions, do not enhance the Fenton reactions. Indeed, the Δ_r_*G*^0^ values of their reduction reactions with the Asc^−^ and O_2_˙^−^ agents are all higher than −29.1 and −26.9 kcal mol^−1^, respectively, which are significantly higher than those of the reduction reaction for [Fe(iii)(H_2_O)_6_]^3+^ complexes (−43.5 and 41.3 kcal mol^−1^, respectively). Thus, the reduction processes of Fe(iii)-to-Fe(ii) complexes are less favorable than that of the Fe(iii) complexes in the aqueous phase. Consequently, the pro-oxidant risks of these complexes are not taken into account.

In order to evaluate the rate of the reduction reactions for Fe(iii)-to-Fe(ii) complexes by the Asc^−^ and the O_2_˙^−^ values, the kinetics data for these SET processes were calculated using the Marcus theory.^71,72^ These kinetic parameters including reorganization energy (*λ*), Gibbs free energy of activation (Δ*G*^≠^, kcal mol^−1^), diffusion rate constant (*k*_D_, M^−1^ s^−1^), TST thermal rate constant (*k*_T_, M^−1^ s^−1^), and diffusion-corrected apparent rate constants (*k*_app_, M^−1^ s^−1^) are shown in Tables 5 and 6 for the superoxide anion (O_2_˙^−^) and the ascorbate anion (Asc^−^), respectively.

Regarding the reactions between the [Fe(iii)(H_2_O)_6_]^3+^ ion and the Fe(iii)–RA^−^ complexes with O_2_˙^−^ (Table 5), almost the complexes have higher reaction rates than that of the [Fe(iii)(H_2_O)_6_]^3+^ ion. Indeed, the reactions for all the Fe(iii)–RA^−^ complexes, except the ones formed at O8, site-3 and site-4, have a *k*_app_ value varying from 8.43 × 10^9^ to 8.58 × 10^9^ M^−1^ s^−1^. These values are higher than that of the [Fe(iii)(H_2_O)_6_]^3+^ ion (*i.e.* 7.63 × 10^9^ M^−1^ s^−1^). This means that these complexes have high risk to the reduction of Fe(iii)-to-Fe(ii) complexes and thus enhance the Fenton-like reactions. Conversely, the *k*_app_ values of the redox reactions between O_2_˙^−^ and the Fe(iii)–RA^−^ complexes obtained at the O8, site-3 and site-4 (*i.e.* 6.16 × 10^9^, 3.42 × 10^9^ and 1.84 × 10^9^ M^−1^ s^−1^, respectively) are smaller than that of the [Fe(iii)(H_2_O)_6_]^3+^ ion. These complexes have high potential to prevent the reduction of the Fe(iii)-to-Fe(ii) complexes by the O_2_˙^−^ agent. However, it is noteworthy that the Fe(iii)–RA^−^ complexes formed at O8, site-3 and site-4 positions are quite negligible (Table 4). Thus, when O_2_˙^−^ is the reducing agent, the Fe(iii)-to-Fe(ii) complex reduction processes are enhanced, and the pro-oxidant risks may be remarkable.

Regarding the redox reaction between the Asc^−^ and Fe(iii) complexes (Table 6), it can be seen that all reactions are fast and favorable with the *k*_app_ value ranging from 2.92 × 10^6^ to 7.62 × 10^9^ M^−1^ s^−1^ for the ones of the Fe(iii)–RA^−^ complexes, which are generally lower or similar to the reaction of the [Fe(iii)(H_2_O)_6_]^3+^ ion (*i.e.* 7.44 × 10^9^ M^−1^ s^−1^). The *k*_app_ value of the reaction of Fe(iii)–RA^−^ complexes formed at O2, O5, O6, site-1, and site-2 are slightly higher than that of the [Fe(iii)(H_2_O)_6_]^3+^ ion, and thus, these reactions occur slightly faster. Meanwhile, the reactions for the other complexes especially the ones at site-3 and site-4 have lower reaction rates. Indeed, the rates of the Fe(iii)–RA^−^ complexes at site-3 and site-4 are approximately 800 and 3000 times lower than that for the reaction of the [Fe(iii)(H_2_O)_6_]^3+^ ion. As a result, these complexes are able to prevent Fe(iii)-to-Fe(ii) reduction processes by the Asc^−^ agent. Therefore, the pro-oxidant risks of these complexes are insignificant.

Overall, the RA^−^ does not enhance the Fe(iii)-to-Fe(ii) reduction process by the ascorbate anion, but it slightly promotes this process when the superoxide anion is considered as the reducing agent. Thus, the RA^−^ may express the pro-oxidant risk depending on the reducing agent present in the environment.

## Conclusions

Based on the direct and indirect antioxidant activities of rosmarinic acid and its pro-antioxidant risks in an aqueous phase using the DFT approach, there are multiple conclusions as follows:

(i) Rosmarinic acid has an antioxidant potential *via* FHT and SET mechanisms with the smallest BDE (O3–H) value being 83.3 kcal mol^−1^ and the IP being 121.2 kcal mol^−1^. These values are similar or lower than several popular antioxidant compounds.

(ii) HAT is the responsible mechanism for HOO˙ and CH_3_OO˙ radical scavenging activities of mono-anion rosmarinate (RA^−^) in the aqueous phase with the negative Gibbs free energies and high rate constants at all –OH positions. Especially, the HAT reaction occurring at O7H represents the most preponderant one with branching ratios of 47.41% for HOO˙ and 71.60% for CH_3_OO˙ radical. In addition, RA^−^ has demonstrated its good antioxidant capacity to HOO˙ and CH_3_OO˙ radicals in comparison to other popular antioxidants with *k*_tot_ values of 1.84 × 10^3^ and 4.49 × 10^3^ M^−1^ s^−1^, respectively. The non-polar pentyl ethanoate solvent slightly enhances FHT reactions, while it is unfavorable to RAF and especially to SET ones compared to the ones in the aqueous phase.

(iii) RA^−^ has remarkable potential to chelate both Fe(iii) and Fe(ii) ions, especially at the O5 position for the mono-dentate complexes and the site-1, site-2 ones for the bi-dentate complexes. Moreover, the chelation process towards Fe(iii) ions is more favorable and spontaneous than that for Fe(ii) ions.

(iv) Reduction processes of Fe(iii)-to-Fe(ii) complexes by Asc^−^ and O_2_˙^−^ agents, which may be an initial step for Fenton-like reactions forming reactive HO˙ radicals were considered in comparison with the self-reduction process of [Fe(iii)(H_2_O)_6_]^3+^ ions. Consequently, RA^−^ may enhance the pro-oxidant risk when O_2_˙^−^ is present in the reactive media; however, this phenomenon is not observed if Asc^−^ is available.

Hopefully, the actual work may provide a multi-facet point of view into the antioxidant potential of rosmarinic acid before further chemical and biological applications.

## Author contributions

Dinh Hieu Truong: investigation – writing. Thi Chinh Ngo: investigation, review & editing. Nguyen Thi Ai Nhung: review & editing. Duong Tuan Quang: conception, review & editing. Thi Le Anh Nguyen: review & editing. Dorra Khiri: writing – review & editing. Sonia Taamalli: review & editing. Abderrahman El Bakali: review & editing. Florent Louis: investigation – review & editing. Duy Quang Dao: conceptualization – project administration – review & editing.

## Conflicts of interest

There are no conflicts to declare.

## Supplementary Material

RA-012-D1RA07599C-s001
