# Supplementary material for: New insights into the competition between antioxidant activities and pro-oxidant risks of rosmarinic acid
Source: RSC Adv. 2022 Jan 10;12(3):1499–514. doi: 10.1039/d1ra07599c (PMC8978883; doi:10.1039/d1ra07599c)
Supplement: RA-012-D1RA07599C-s001 [file RA-012-D1RA07599C-s001.pdf]

Electronic Supplementary Material (ESI) for RSC Advances.
This journal is © The Royal Society of Chemistry 2021

Supporting information

New insight into the competition between antioxidant activities and pro-oxidant risks of rosmarinic acid

Dinh Hieu Truong,[1,2] Thi Chinh Ngo,[1,2] Nguyen Thi Ai Nhung,[3] Duong Tuan Quang,[4] Thi Le Anh Nguyen,[1,2] Dorra Khiri[5], Sonia Taamalli[5], Florent Louis[5], Abderrahman El Bakali[5] and Duy Quang Dao[1,2,*]

[1]*Institute of Research and Development, Duy Tan University, Da Nang, 550000, Vietnam*

[2]*Faculty of Natural Sciences, Duy Tan University, Da Nang, 550000, Vietnam*

[3]*Department of Chemistry, University of Sciences, Hue University, Hue, 530000, Vietnam*

[4]*Department of Chemistry, University of Education, Hue University, Hue, 530000, Vietnam*

[5]*Université de Lille, CNRS, UMR 8522 – PC2A – PhysicoChimie des Processus de Combustion et de l'Atmosphère, 59000 Lille, France*

List of Figures

Figure S1: Optimized structure of rosmarinic acid in neutral form (**A**) and mono-anion one (**B**) in water phase at 298.15 K at the M05-2X/6–311++G(2df,2p) level of theory. The numbers are bond distances (in black in Å), bond angles (in red in degree) and tehedral angles (in blue in degree).

Figure S2: The total energy of RA deprotonated at the C9-H position as function of 68 optimization steps. Four figures are corresponding to the structures obtained at the step 1, step 14, step 20 and step 68.

Figure S3: IP values (in kcal mol^{-1}) for rosmarinic acid, trolox and ascorbic acid at 298.15K calculated in water and PEA at the M05-2X/6–311++G(2df,2p) level of theory.

List of Tables

Table S1: Cartesian coordinates and thermochemistry properties of the transition states (TSs) for FHT and RAF reaction of rosmarinate mono-anion (RA^{-}) towards $HOO^{\bullet}$ radical calculated in water at the M05-2X/6–311++G(2df,2p) level of theory.

Table S2: Cartesian coordinates and thermochemistry properties of the transition states (TSs) for FHT and RAF reaction of rosmarinate mono-anion (RA^{-}) towards $CH_3OO^{\bullet}$ radical calculated in water at the M05-2X/6–311++G(2df,2p) level of theory.

Table S3: Cartesian coordinates and thermochemistry properties of the transition states (TSs) for FHT and RAF reaction of rosmarinate mono-anion (RA^{-}) towards $HOO^{\bullet}$ and $CH_3OO^{\bullet}$ radicals calculated in PEA at the M05-2X/6–311++G(2df,2p) level of theory.

Table S4: Dipole moment values of rosmarinic acid anion (RA^{-}), the studied free radicals ($HOO^{\bullet}$ and $CH_3OO^{\bullet}$) and all the transition states of FHT and RAF reactions calculated in the aqueous phase. Gibbs energies of activation values ($\Delta G^{\#}$) are also resumed for comparison.

Table S5: NBO analyses calculated at the transition states (TSs) for FHT reaction between RA^{-} with $HOO^{\bullet}$ and $CH_3OO^{\bullet}$ radical in aqueous phase.

Table S6: NPA charges, atomic spin densities (ASD), 1S occupancy of shifting-H, natural electron configuration (NEC) calculated at the transition states (TSs) for shifting-H, donor and acceptor of RA^{-}.

Table S7: Cartesian coordinates and thermochemistry properties of optimized structures of 7 monodentate complexes types and 4 bidentate ones between the rosmarinate mono-anion (RA^{-}) and $[Fe(II).6H_2O]^{2+}$ ion in water calculated at the M05-2X/6–311++G(2df,2p) level of theory.

Table S8: Cartesian coordinates and thermochemistry properties of optimized structures of 7 monodentate complexes types and 4 bidentate ones between the rosmarinate mono-anion (RA^{-}) and $[Fe(III).6H_2O]^{3+}$ ion in water calculated at the M05-2X/6–311++G(2df,2p) level of theory.

Table S9: Cartesian coordinates and thermochemistry properties of optimized structures of 7 monodentate complexes types and 4 bidentate ones between the neutral rosmarinic (RA) and $[Fe(II).6H_2O]^{2+}$ ion in water calculated at the M05-2X/6–311++G(2df,2p) level of theory.

Table S10: Cartesian coordinates and thermochemistry properties of optimized structures of 7 monodentate complexes types and 4 bidentate ones between the neutral rosmarinic (RA) and $[Fe(III).6H_2O]^{3+}$ ion in water calculated at the M05-2X/6–311++G(2df,2p) level of theory.

Table S11: Cartesian coordinates and thermochemistry properties of ascorbate mono-anion, ascobate radical, superoxide anion radical, oxygen molecule, neutral rosmarinic, mono-anion rosmarinate and aqueous iron complexes in water calculated at the M05-2X/6–311++G(2df,2p) level of theory.

Table S12: Reaction enthalpies ($\Delta_r H^0$) and standard Gibbs free energies ($\Delta_r G^0$) and formation constants (K_f) of complexation reaction between the neutral rosmarinic (RA) and $[Fe(II).6H_2O]^{2+}$ and $[Fe(III).6H_2O]^{3+}$ ions in water phase at 298.15 K at the M05-2X/6–311++G(2df,2p) level of theory.

Table S13: The standard enthalpy ($\Delta_r \boldsymbol{H}^0$) and Gibbs free energy ($\Delta_r \boldsymbol{G}^0$) for the redox reaction between superoxide anion ($O_2^{\bullet -}$) and the iron complexes of neutral-rosmarinic form in water phase at the M05-2X/6–311++G(2df,2p) level of theory.

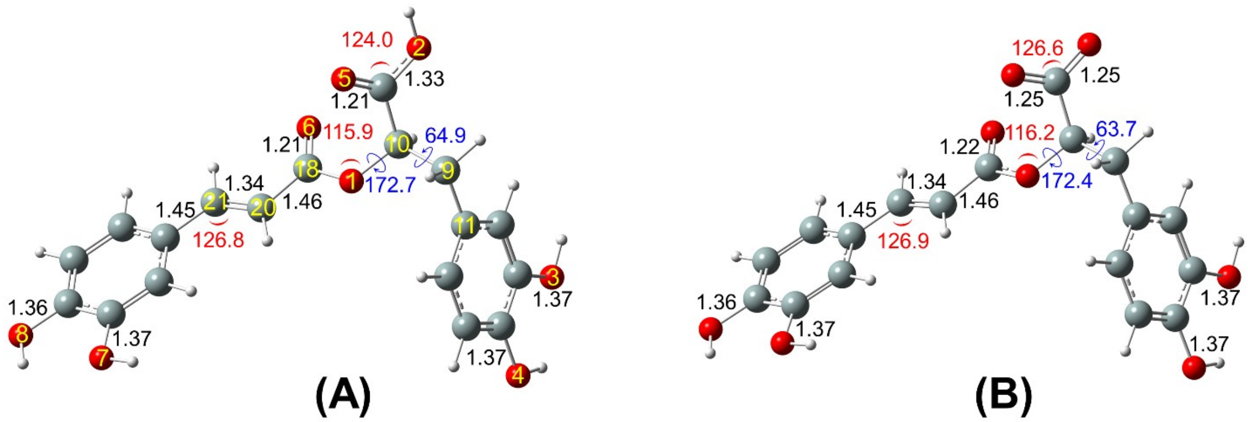

Figure S1: Optimized structure of rosmarinic acid in neutral form (**A**) and mono-anion one (**B**) in water phase at 298.15 K at the M05-2X/6–311++G(2df,2p) level of theory. The numbers are bond distances (in black in Å), bond angles (in red in degree) and tehedral angles (in blue in degree).

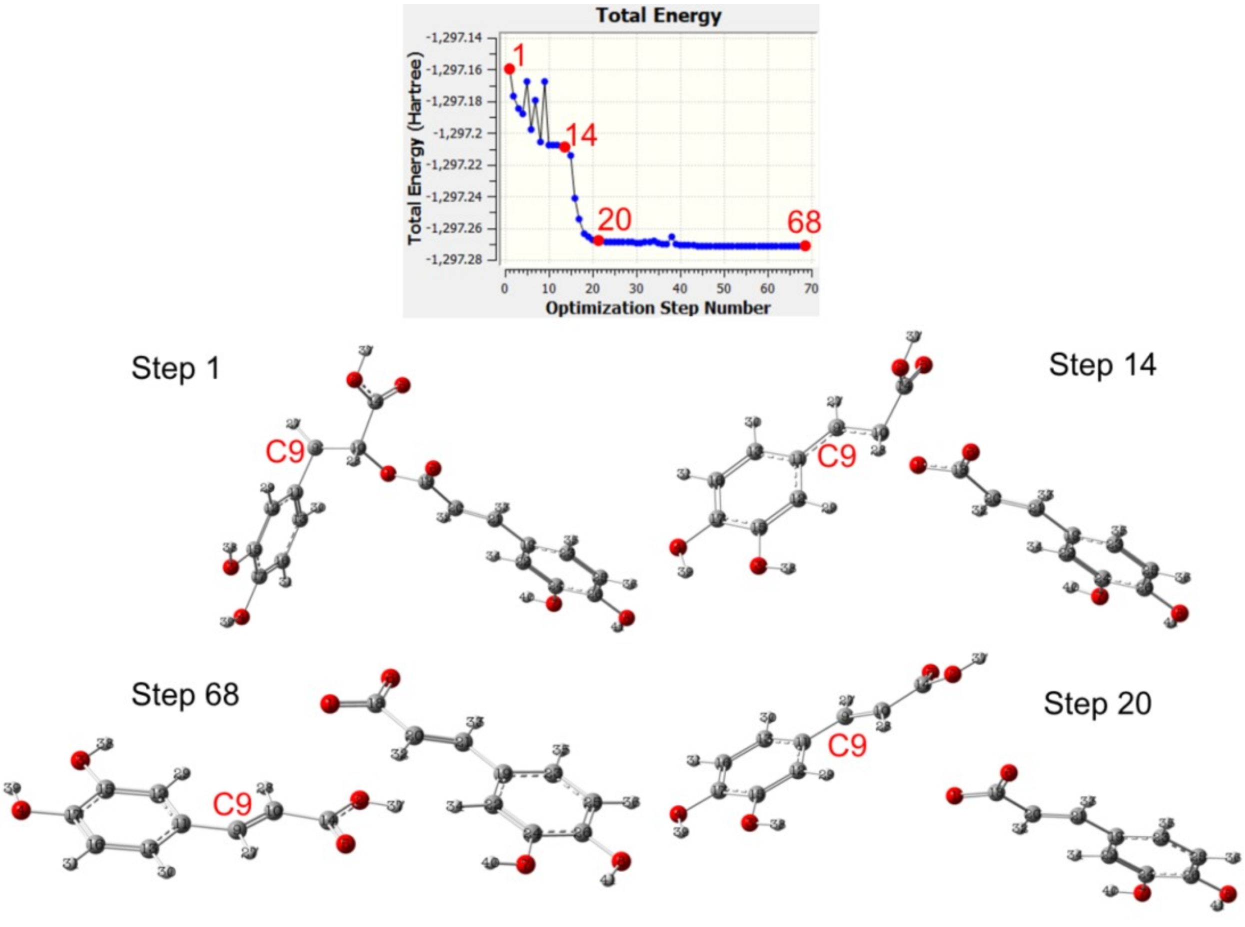

Figure S2: The total energy of RA deprotonated at the C9-H position as function of 68 optimization steps. Four figures are corresponding to the structures obtained at the step 1, step 14, step 20 and step 68.

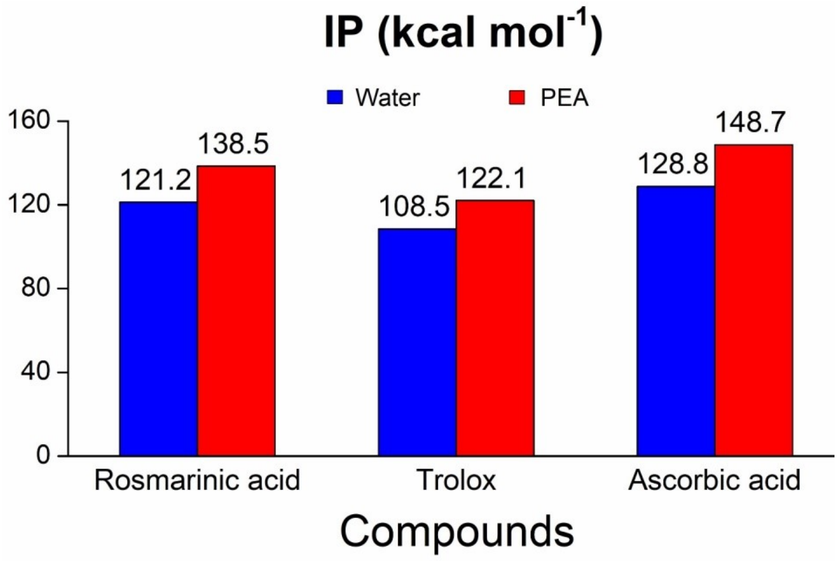

Figure S3: Adiabatic IP values (in kcal mol^{-1}) for rosmarinic acid, trolox and ascorbic acid at 298.15K calculated in water and PEA at the M05-2X/6–311++G(2df,2p) level of theory.

Table S1: Cartesian coordinates and thermochemistry properties of the transition states (TSs) for FHT and RAF reaction of rosmarinate mono-anion (RA^-) towards $HOO^\bullet$ radical calculated in water at the M05-2X/6–311++G(2df,2p) level of theory.

**TS-FHT-Rosmarinic-anion-O3-HOO**				
-1 2				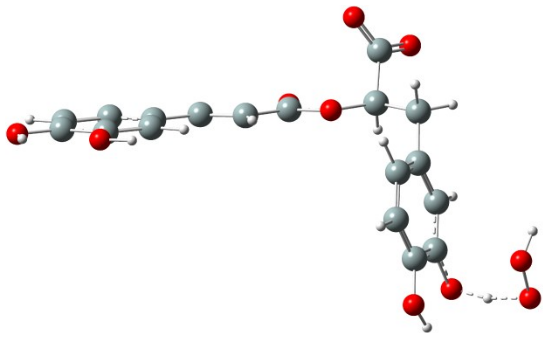
O	-0.31145000	1.48295500	-0.33982800	
O	-1.96845900	4.60912800	0.02907400	
O	-4.79944200	-1.55141000	1.81288900	
O	-3.82835600	-3.57250200	0.28620500	
O	-0.23258200	4.02471500	-1.25709100	
O	0.73584800	2.53399700	1.33859000	
O	5.60239600	-2.57012500	-1.54447600	
O	7.72535700	-2.01636800	-0.00007000	
C	-2.57169200	1.77858800	-1.00189200	
C	-1.45684100	2.31535700	-0.11312900	
C	-2.93116000	0.35994400	-0.66060800	
C	-3.73926800	0.08093200	0.41948700	
C	-2.42477200	-0.70619300	-1.42175800	
C	-1.17447600	3.77834800	-0.47327600	
C	-4.04035500	-1.24220000	0.75826300	
C	-2.71438200	-2.02077400	-1.11663700	
C	-3.52269000	-2.29846400	-0.02594500	
C	0.74711000	1.70381000	0.44959400	
C	4.22406200	0.13983200	0.53538600	
C	1.86597700	0.82935800	0.10135900	
C	3.02189300	0.92735400	0.76350600	
C	4.29309700	-0.86144200	-0.43993500	
C	5.34620800	0.39731900	1.31871200	
C	5.45386400	-1.57581300	-0.61643100	
C	6.51713400	-0.32197300	1.14028100	
C	6.57520800	-1.30806100	0.17554900	
H	-3.43311700	2.42679000	-0.86490700	
H	-2.25188000	1.84481700	-2.03956800	
H	-1.73843900	2.25291400	0.93312000	
H	-4.15256500	0.87207900	1.02782400	
H	-1.79604800	-0.48649300	-2.27107900	
H	-2.32818500	-2.83413700	-1.70962400	
H	1.69999100	0.13067000	-0.70186100	
H	3.09274600	1.66400800	1.55281000	
H	3.44557300	-1.09167400	-1.06781900	
H	5.29887200	1.16822600	2.07206700	
H	7.39065800	-0.12793100	1.74215000	
H	-4.37640100	-3.57751700	1.08396200	
H	4.79018900	-2.67863100	-2.05145200	
H	7.60003100	-2.66210800	-0.70626300	

O	-6.85516700	-1.96384600	0.66290500
O	-6.30155100	-1.74927900	-0.54956800
H	-5.79404800	-1.77774800	1.45210800
H	-6.39361600	-0.79690900	-0.71565700

Zero-point correction= 0.322121 (Hartree/Particle)
Thermal correction to Energy= 0.348375
Thermal correction to Enthalpy= 0.349319
Thermal correction to Gibbs Free Energy= 0.260852
Sum of electronic and zero-point Energies= -1447.894861
Sum of electronic and thermal Energies= -1447.868607
Sum of electronic and thermal Enthalpies= -1447.867663
Sum of electronic and thermal Free Energies= -1447.956130

TS-FHT-Rosmarinic-anion-O4-HOO

-1 2

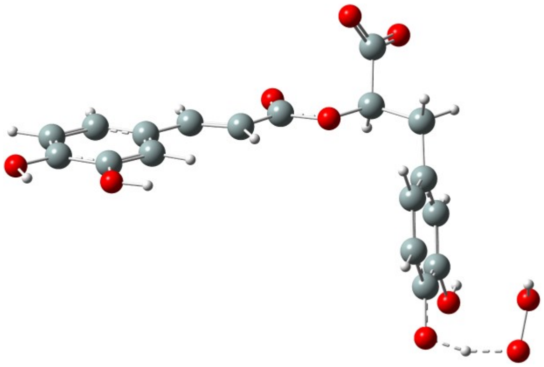

O	-0.30039200	1.58336100	-0.35886600
O	-1.86970200	4.76083100	-0.03476000
O	-4.81474400	-1.19947600	2.06884400
O	-0.17410300	4.10129000	-1.33870500
O	0.81378000	2.63900800	1.27270400
O	5.42706700	-2.75636200	-1.49716000
O	7.60195800	-2.21636400	-0.02139900
C	-2.56827900	1.92413500	-0.96951600
C	-1.41532500	2.45319300	-0.12440300
C	-2.96341700	0.53428800	-0.56441800
C	-3.72336200	0.33968600	0.57879000
C	-2.54618700	-0.57117000	-1.31991100
C	-1.10569400	3.89894200	-0.53092000
C	-4.06901100	-0.94055900	0.97579400
C	-2.89082300	-1.84580800	-0.94029700
C	-3.64995200	-2.05917900	0.21716000
C	0.78063700	1.78733500	0.40503800
C	4.19494900	0.09161800	0.48775800
C	1.86096400	0.86451900	0.06054600
C	3.02831900	0.93325400	0.70614000
C	4.20593700	-0.95304600	-0.44351300
C	5.34179300	0.33895500	1.23793500
C	5.33541500	-1.71797800	-0.61067400
C	6.48136400	-0.43094300	1.06825300
C	6.48260200	-1.45877400	0.14625100
H	-3.40504900	2.60452100	-0.83449000
H	-2.27053400	1.94020600	-2.01522500
H	-1.66963700	2.42723900	0.93017600
H	-4.05877500	1.18261500	1.16687900
H	-1.95608500	-0.40895100	-2.20822400
H	-2.58460000	-2.70806900	-1.51153400
H	1.65677000	0.15407600	-0.72327100
H	3.13810100	1.68766300	1.47389900
H	3.33700800	-1.18020800	-1.04271900
H	5.33912300	1.14286500	1.95753300
H	7.37404500	-0.24432000	1.64380000

H	4.60183300	-2.85379900	-1.98525000
H	7.44023300	-2.88117000	-0.70194100
O	-3.96824600	-3.28874000	0.62094900
H	-5.01553000	-3.44170500	0.42155700
O	-6.25530900	-3.30773200	-0.08392500
O	-6.16953500	-2.06450800	-0.60707700
H	-5.06437600	-0.37644700	2.50562100
H	-5.86830400	-2.18580000	-1.52172600

Zero-point correction= 0.321566 (Hartree/Particle)
Thermal correction to Energy= 0.348017
Thermal correction to Enthalpy= 0.348962
Thermal correction to Gibbs Free Energy= 0.259961
Sum of electronic and zero-point Energies= -1447.894327
Sum of electronic and thermal Energies= -1447.867875
Sum of electronic and thermal Enthalpies= -1447.866931
Sum of electronic and thermal Free Energies= -1447.955932

TS-FHT-Rosmarinic-anion-O7-HOO

-1 2

O	1.55186400	1.20637900	0.27518100
O	3.60431400	4.10987400	0.05854900
O	5.80367900	-2.27390800	-1.63720300
O	4.65833100	-4.19134200	-0.12043000
O	1.63032900	3.77565200	1.05872100
O	0.80737700	2.23535600	-1.57111900
O	-4.90514500	-1.97091500	1.09860200
O	-6.74145600	-1.43235500	-0.81429300
C	3.76534500	1.26153300	1.12993700
C	2.80090000	1.89917600	0.13870200
C	4.01671000	-0.18896300	0.82092800
C	4.81265700	-0.53751800	-0.26773800
C	3.44279900	-1.19719200	1.58082700
C	2.64685100	3.39444100	0.43963000
C	5.02789200	-1.86382900	-0.58316900
C	3.65776400	-2.53385300	1.26576300
C	4.44746300	-2.87018500	0.18556900
C	0.61349400	1.48580800	-0.63423400
C	-2.98516000	0.27889500	-1.01765900
C	-0.63493300	0.77187700	-0.35117700
C	-1.68229300	0.91623100	-1.16428800
C	-3.30576500	-0.54978700	0.04248900
C	-3.95753000	0.52684700	-2.00734100
C	-4.56660600	-1.14134800	0.11598100
C	-5.21260300	-0.03581000	-1.95280700
C	-5.52774200	-0.87412400	-0.89407100
H	4.69385300	1.82474600	1.08139400
H	3.35465700	1.36525000	2.13206400
H	3.16327000	1.77934100	-0.87722200
H	5.27442400	0.22671300	-0.87830200
H	2.82355700	-0.94118600	2.42687200
H	3.21581000	-3.32323600	1.85368300

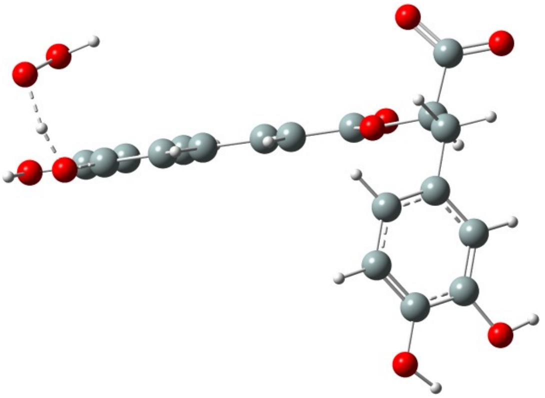

H	-0.64560300	0.14523100	0.52545300
H	-1.57396500	1.56386500	-2.02384400
H	-2.60248600	-0.76827500	0.83035200
H	-3.70318400	1.17846400	-2.82883300
H	-5.95198200	0.15989400	-2.71204100
H	6.16908100	-1.50812300	-2.09312800
H	5.22208600	-4.24955000	-0.90039000
H	-5.50745300	-1.40565500	1.82600700
H	-6.78171100	-1.99464500	-0.02666000
O	-6.15199800	-0.45690100	2.44205500
O	-6.01986900	0.55732100	1.55629500
H	-5.20520900	1.02068300	1.80967700

Zero-point correction= 0.321240 (Hartree/Particle)
Thermal correction to Energy= 0.347752
Thermal correction to Enthalpy= 0.348697
Thermal correction to Gibbs Free Energy= 0.259065
Sum of electronic and zero-point Energies= -1447.893779
Sum of electronic and thermal Energies= -1447.867267
Sum of electronic and thermal Enthalpies= -1447.866323
Sum of electronic and thermal Free Energies= -1447.955955

TS-FHT-Rosmarinic-anion-O8-HOO

-1 2

O	-1.61702500	1.18667300	-0.30351200
O	-3.58760600	4.13869400	-0.03088600
O	-5.92093900	-2.19052200	1.68211900
O	-4.86923600	-4.13550600	0.13477000
O	-1.66339000	3.75160400	-1.10652000
O	-0.81384800	2.21792300	1.51646600
O	4.78416900	-2.03922800	-1.32287800
C	-3.84335500	1.29243100	-1.12167900
C	-2.84931200	1.90640200	-0.14559100
C	-4.12722100	-0.15132700	-0.80911400
C	-4.90832300	-0.47956500	0.29649900
C	-3.60131200	-1.17353600	-1.58493500
C	-2.66572300	3.39805300	-0.44901600
C	-5.15627800	-1.79982800	0.61286200
C	-3.84940200	-2.50434700	-1.26891700
C	-4.62439900	-2.82049700	-0.17215100
C	-0.65534800	1.45990900	0.58094100
C	2.93825100	0.21910500	0.88063700
C	0.58018500	0.72895700	0.27330900
C	1.64768400	0.86811800	1.06157700
C	3.20657900	-0.62907400	-0.19159200
C	3.93937900	0.47746000	1.83431300
C	4.44897800	-1.21489000	-0.31181200
C	5.18004900	-0.09495500	1.71808600
C	5.45989800	-0.96053100	0.65189200
H	-4.75674900	1.87878700	-1.05969900
H	-3.44523000	1.38623200	-2.12979500
H	-3.19611800	1.79335700	0.87640000

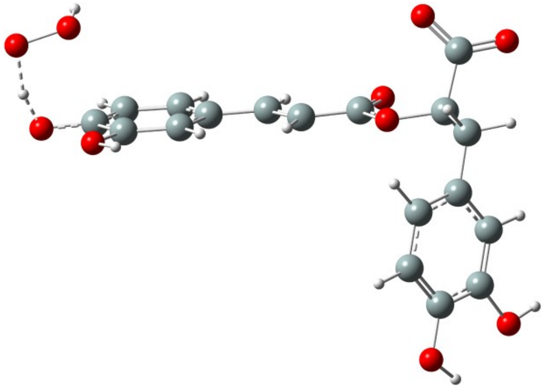

H	-5.33322700	0.29612300	0.91922500
H	-2.99437100	-0.93319400	-2.44437800
H	-3.44534000	-3.30457600	-1.86931600
H	0.56526600	0.09989500	-0.60128500
H	1.56831800	1.52013300	1.92049300
H	2.46065100	-0.84087100	-0.94217900
H	3.71818600	1.13706500	2.65831000
H	5.96052600	0.09285800	2.43798300
H	-6.24041000	-1.41616700	2.15779400
H	-5.41798400	-4.17872000	0.92639500
H	4.04679700	-2.13176200	-1.93824100
O	6.63623500	-1.56091400	0.54353600
H	7.16582600	-1.12022300	-0.29881700
O	7.54953000	-0.27308700	-1.24211200
O	6.49203500	0.56968600	-1.26157900
H	6.70247700	1.26715300	-0.61997300

Zero-point correction= 0.321465 (Hartree/Particle)
Thermal correction to Energy= 0.347946
Thermal correction to Enthalpy= 0.348890
Thermal correction to Gibbs Free Energy= 0.259699
Sum of electronic and zero-point Energies= -1447.891926
Sum of electronic and thermal Energies= -1447.865445
Sum of electronic and thermal Enthalpies= -1447.864501
Sum of electronic and thermal Free Energies= -1447.953692

TS-RAF-Rosmarinic-anion-C20-HOO

-1 2

O	1.27414500	-1.20841300	-0.20090300
O	3.49705900	-3.87730200	0.59094200
O	4.63604700	2.93255800	1.84562400
O	3.73972700	4.50226400	-0.15299400
O	1.71811400	-3.81144900	-0.76610800
O	0.35399000	-2.21386100	1.57699100
O	-5.00750700	2.05830600	-1.69276100
O	-6.88020800	1.88272600	0.21666700
C	3.58297700	-1.12832600	-0.75466000
C	2.54941200	-1.76742300	0.16156500
C	3.63575500	0.36760200	-0.60513100
C	4.13081100	0.93477400	0.56718500
C	3.17571000	1.20304700	-1.61172900
C	2.56999800	-3.28958800	-0.01575800
C	4.16300200	2.30616300	0.72105400
C	3.21181500	2.58441500	-1.46132800
C	3.70436700	3.13825800	-0.29812900
C	0.25231700	-1.51577500	0.59179700
C	-3.27656400	-0.09106500	0.75397000
C	-1.01223500	-0.91641200	0.11196100
C	-2.05562600	-0.79655800	0.99645200
C	-3.50846000	0.65696300	-0.41506400
C	-4.28563700	-0.15281300	1.72578900
C	-4.70233200	1.30907400	-0.59076100

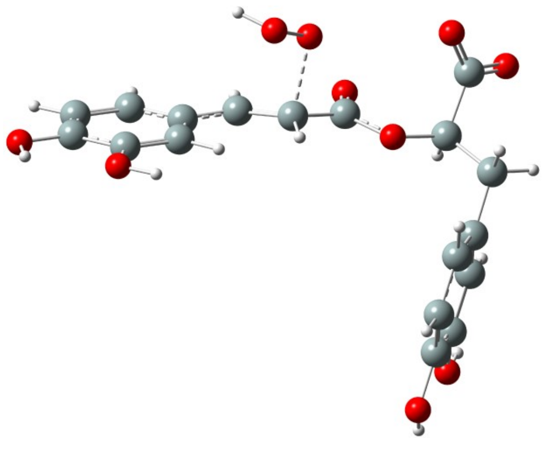

C	-5.48604200	0.50268300	1.54423100
C	-5.70095600	1.23520800	0.38896200
H	4.54508000	-1.56965100	-0.50543200
H	3.34545700	-1.38939100	-1.78370700
H	2.75076800	-1.52659200	1.20027800
H	4.49724100	0.30931000	1.36998900
H	2.78745300	0.77580500	-2.52351400
H	2.85830900	3.23972700	-2.24213400
H	-0.93337300	-0.29085400	-0.76039000
H	-1.98191000	-1.33010800	1.93266100
H	-2.75728400	0.73857600	-1.18542200
H	-4.11264800	-0.72359300	2.62456100
H	-6.26749500	0.45964600	2.28603500
H	4.90122300	2.27872000	2.50098800
H	4.11501400	4.71868600	0.70836200
H	-4.26159800	2.07394600	-2.30240100
H	-6.87281000	2.35350100	-0.62601100
O	-1.46143500	-2.51703500	-0.93995500
O	-2.49588800	-2.15177600	-1.76390500
H	-3.29565000	-2.36723600	-1.26508000

Zero-point correction= 0.325936 (Hartree/Particle)
Thermal correction to Energy= 0.352395
Thermal correction to Enthalpy= 0.353340
Thermal correction to Gibbs Free Energy= 0.265488
Sum of electronic and zero-point Energies= -1447.891184
Sum of electronic and thermal Energies= -1447.864724
Sum of electronic and thermal Enthalpies= -1447.863780
Sum of electronic and thermal Free Energies= -1447.951632

TS-RAF-Rosmarinic-anion-C21-HOO

-1 2

O	-1.22307100	1.07076100	-0.31450200
O	-3.08081400	4.06143300	0.20518300
O	-5.59225400	-2.22077300	1.78466800
O	-4.65474100	-4.15290700	0.15500600
O	-1.24600700	3.66660300	-1.01414300
O	-0.27417800	2.02502200	1.48277000
O	5.00772200	-2.33711000	-1.76150200
O	6.89244200	-2.19589800	0.14602800
C	-3.49072500	1.27183100	-0.98772900
C	-2.42113400	1.81574000	-0.05064500
C	-3.81148700	-0.17052200	-0.70673500
C	-4.56685400	-0.50432500	0.41501000
C	-3.34598600	-1.18683200	-1.52772700
C	-2.21229600	3.31369500	-0.30459100
C	-4.84871100	-1.82421900	0.70279900
C	-3.62719100	-2.51746800	-1.23957300
C	-4.37618600	-2.83894400	-0.12640000
C	-0.19588400	1.29714200	0.51036100
C	3.35946200	-0.09267100	0.64589500
C	0.99916300	0.57517100	0.10200900

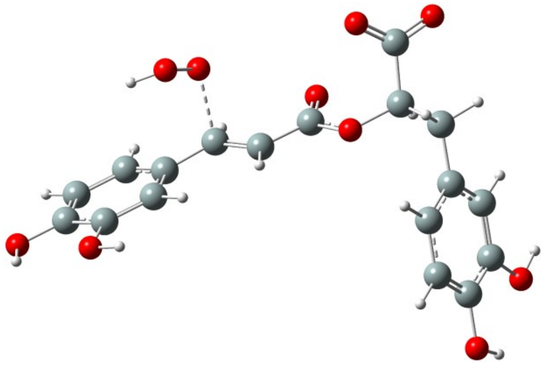

C	2.15555000	0.70790400	0.84952500	
C	3.55854000	-0.86218600	-0.50311600	
C	4.36300700	-0.03358900	1.60876100	
C	4.73301100	-1.56182100	-0.66830500	
C	5.54273600	-0.73851000	1.44241700	
C	5.73070700	-1.50366500	0.30671600	
H	-4.37690400	1.88726500	-0.85411700	
H	-3.14559800	1.38734500	-2.01298700	
H	-2.70864300	1.66974400	0.98563800	
H	-4.94554000	0.26697700	1.07205300	
H	-2.76035400	-0.94187900	-2.40055100	
H	-3.26935600	-3.31302500	-1.87450600	
H	0.96104600	0.02755000	-0.82333700	
H	2.05395600	1.14855700	1.82909700	
H	2.80866500	-0.92435900	-1.27722500	
H	4.21414600	0.56756700	2.49228500	
H	6.32539100	-0.70389200	2.18351300	
H	-5.87631500	-1.45039400	2.28852700	
H	-5.18028100	-4.19733600	0.96232300	
H	4.26374300	-2.32587100	-2.37339400	
H	6.86763400	-2.67684400	-0.69003500	
O	2.64677600	2.42728000	0.10602300	
O	3.42668300	2.25204500	-0.99739700	
H	4.27362900	1.92655800	-0.65639900	

Zero-point correction= 0.325521 (Hartree/Particle)
Thermal correction to Energy= 0.352019
Thermal correction to Enthalpy= 0.352963
Thermal correction to Gibbs Free Energy= 0.264571
Sum of electronic and zero-point Energies= -1447.887122
Sum of electronic and thermal Energies= -1447.860624
Sum of electronic and thermal Enthalpies= -1447.859680
Sum of electronic and thermal Free Energies= -1447.948071

Table S2: Cartesian coordinates and thermochemistry properties of the transition states (TSs) for FHT and RAF reaction of rosmarinate mono-anion (RA^{-}) towards $CH_3OO^{\bullet}$ radical calculated in water at the M05-2X/6–311++G(2df,2p) level of theory.

**TS-FHT-Rosmarinic-anion-O3-CH_3OO**			
-1 2			
O	-0.04694000	1.53008400	-0.30394700
O	-1.65624700	4.67976800	0.08121400
O	-4.58181800	-1.41946600	1.93113800
O	-3.57498400	-3.47643900	0.44701100
O	0.04455400	4.06596700	-1.23775500
O	1.05154400	2.57537700	1.34479400
O	5.76850100	-2.65673000	-1.56097100
O	7.93288700	-2.11119600	-0.07327900
C	-2.31855000	1.85192400	-0.91394800
C	-1.17529000	2.37926500	-0.05575200
C	-2.68697500	0.44053300	-0.55458500
C	-3.48948400	0.17927900	0.53148300
C	-2.19466300	-0.63828600	-1.31312900
C	-0.88306400	3.83625700	-0.43224400
C	-3.80666900	-1.13706300	0.88488500
C	-2.48562300	-1.94456800	-0.98789800
C	-3.28533100	-2.20599700	0.11665800
C	1.03131900	1.73903000	0.46179200
C	4.48101400	0.11428800	0.49698400
C	2.12797300	0.84298000	0.09816300
C	3.29759000	0.92589000	0.73793100
C	4.51198300	-0.90525900	-0.46123800
C	5.62375300	0.36584800	1.25195900
C	5.65637700	-1.64295000	-0.64880500
C	6.77841800	-0.37624000	1.06158500
C	6.79895400	-1.38023300	0.11397000
H	-3.16818100	2.51147700	-0.75909100
H	-2.02349400	1.90771500	-1.95952400
H	-1.43289200	2.32719100	0.99718100
H	-3.89854700	0.98098500	1.12867300
H	-1.57433600	-0.43021900	-2.17139000
H	-2.10644700	-2.76870100	-1.57041800
H	1.93422800	0.14109400	-0.69597900
H	3.39597900	1.66876700	1.51838800
H	3.64728800	-1.13289200	-1.06630300
H	5.60563300	1.15068500	1.99212400
H	7.66787400	-0.18653200	1.64110200
H	-4.11009400	-3.48575200	1.25301000
H	4.94452900	-2.76128700	-2.04956600
H	7.78263700	-2.76408000	-0.76790600
O	-6.55239800	-2.08829100	0.75402100
O	-5.99346400	-1.83638700	-0.44977400
C	-6.42779700	-0.56053600	-0.93891400
H	-6.28746600	0.18679000	-0.16565700

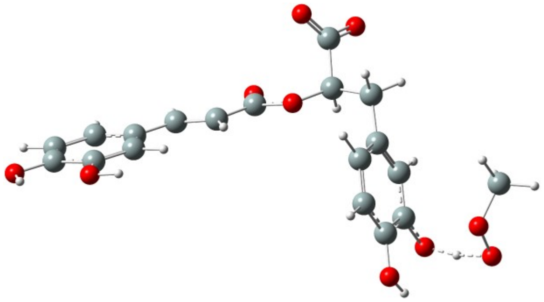

H	-5.80918300	-0.35390900	-1.80478200
H	-7.47447600	-0.63602400	-1.21789800
H	-5.54973000	-1.76177100	1.54504500

Zero-point correction= 0.351183 (Hartree/Particle)
Thermal correction to Energy= 0.378688
Thermal correction to Enthalpy= 0.379632
Thermal correction to Gibbs Free Energy= 0.288223
Sum of electronic and zero-point Energies= -1487.175063
Sum of electronic and thermal Energies= -1487.147558
Sum of electronic and thermal Enthalpies= -1487.146614
Sum of electronic and thermal Free Energies= -1487.238023

TS-FHT-Rosmarinic-anion-O4-CH_3OO

-1 2

O	-0.00921400	1.64119800	-0.35404400
O	-1.46872800	4.87560500	-0.07317800
O	-4.54028600	-0.89032400	2.27231300
O	0.19115000	4.13481000	-1.37964900
O	1.16219600	2.67892100	1.24845500
O	5.59705300	-2.86239400	-1.53087200
O	7.78112800	-2.39857600	-0.04441600
C	-2.27622900	2.04637400	-0.92717600
C	-1.08900000	2.55378200	-0.11704600
C	-2.71719300	0.68680000	-0.47083400
C	-3.44928400	0.55704400	0.69689700
C	-2.36446700	-0.45969800	-1.20278000
C	-0.73782100	3.97965300	-0.55870200
C	-3.83243700	-0.69710100	1.14401500
C	-2.75317400	-1.70554900	-0.77947000
C	-3.49450700	-1.85530600	0.40080300
C	1.08916700	1.81868500	0.39175700
C	4.44792000	0.01538800	0.46079900
C	2.13470800	0.85782100	0.04382500
C	3.30885800	0.89357500	0.67987400
C	4.42905800	-1.02544900	-0.47475700
C	5.59941400	0.22325700	1.21599600
C	5.53421700	-1.82551300	-0.64006300
C	6.71408700	-0.58265800	1.04860400
C	6.68595900	-1.60643100	0.12258700
H	-3.08355900	2.76264200	-0.79886700
H	-1.99712100	2.01915000	-1.97776800
H	-1.32339600	2.55882200	0.94241500
H	-3.72834500	1.42929900	1.27147900
H	-1.79058500	-0.34765800	-2.10936400
H	-2.50387400	-2.59462600	-1.33708800
H	1.89976500	0.14833000	-0.73225200
H	3.44678300	1.64891000	1.44212300
H	3.55650200	-1.22188400	-1.07951000
H	5.61970100	1.02419900	1.93861200
H	7.60996100	-0.42715200	1.62840500
H	4.76932200	-2.93505300	-2.01892800
H	7.60028400	-3.05460900	-0.72858000

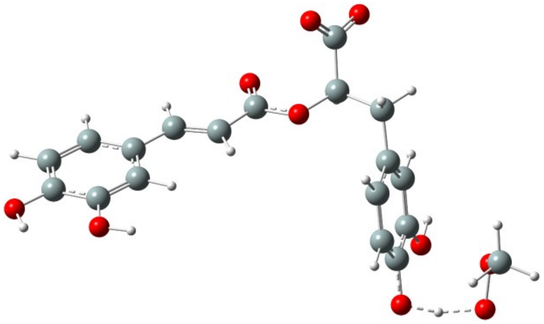

O	-3.86890800	-3.05446700	0.83391500
H	-4.95869200	-3.09670400	0.78298100
O	-6.21859600	-2.86347700	0.44490400
O	-6.06679900	-1.69681500	-0.22184400
C	-6.02808900	-1.94383000	-1.63306400
H	-7.01566800	-2.25731100	-1.95792700
H	-5.75060800	-1.00001000	-2.08833200
H	-5.29217500	-2.71325000	-1.84128300
H	-4.73737900	-0.04401500	2.69162100

Zero-point correction= 0.350341 (Hartree/Particle)
Thermal correction to Energy= 0.378092
Thermal correction to Enthalpy= 0.379036
Thermal correction to Gibbs Free Energy= 0.286328
Sum of electronic and zero-point Energies= -1487.174842
Sum of electronic and thermal Energies= -1487.147091
Sum of electronic and thermal Enthalpies= -1487.146147
Sum of electronic and thermal Free Energies= -1487.238855

TS-FHT-Rosmarinic-anion-O7-CH_3OO

-1 2

O	-1.74028200	1.19858800	-0.19394700
O	-3.73653000	4.12833200	0.15000700
O	-6.11825000	-2.29239300	1.44470300
O	-4.95222400	-4.14822900	-0.12559500
O	-1.75886700	3.80609900	-0.84675500
O	-1.00116600	2.12437000	1.70802500
O	4.68428000	-1.98237500	-1.14582400
O	6.41842000	-1.78931800	0.94120900
C	-3.93861400	1.34333200	-1.07685200
C	-2.97753200	1.90869400	-0.03948500
C	-4.22252500	-0.11588800	-0.84792500
C	-5.05833600	-0.50399900	0.19669200
C	-3.63920800	-1.09487400	-1.63842200
C	-2.78978900	3.41408500	-0.25907100
C	-5.30264700	-1.84084000	0.43872800
C	-3.88330000	-2.44173500	-1.39692800
C	-4.71222400	-2.81745200	-0.36006800
C	-0.81049600	1.41383800	0.74078600
C	2.74666600	0.09064800	1.12360500
C	0.42489200	0.68188300	0.44463300
C	1.46142600	0.76071100	1.28012200
C	3.08746900	-0.63423400	-0.00189800
C	3.68020000	0.19824100	2.17755800
C	4.32663600	-1.27108300	-0.08414700
C	4.90553400	-0.42103200	2.12603100
C	5.23584700	-1.16978800	1.00350200
H	-4.85672000	1.92173000	-1.01257600
H	-3.50968000	1.49092300	-2.06576800
H	-3.35748900	1.74303100	0.96353700
H	-5.52876400	0.23670600	0.82927800
H	-2.98929900	-0.80747700	-2.45069400
H	-3.43413700	-3.20833700	-2.00902300

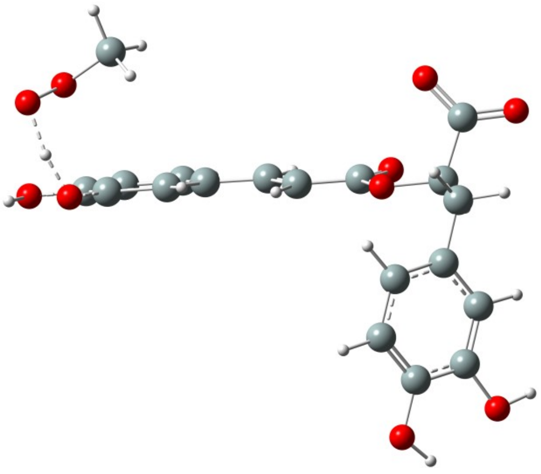

H	0.43370800	0.09155300	-0.45688500
H	1.35493200	1.36682700	2.16965600
H	2.42092800	-0.72480000	-0.84496500
H	3.41307500	0.77919500	3.04661100
H	5.61309400	-0.34296100	2.93523400
H	-6.48663700	-1.54673700	1.93042900
H	-5.54586300	-4.23466300	0.62933300
H	5.46159800	-1.39886500	-1.69665200
H	6.47976100	-2.28827800	0.11355100
O	6.29209200	-0.48978400	-2.06634100
O	6.06567400	0.44946800	-1.11893200
C	5.18939900	1.46200800	-1.63191800
H	5.72747400	2.04050900	-2.37666500
H	4.31415800	0.99201900	-2.06729900
H	4.92429600	2.07648400	-0.77915400

Zero-point correction= 0.350477 (Hartree/Particle)
Thermal correction to Energy= 0.378352
Thermal correction to Enthalpy= 0.379296
Thermal correction to Gibbs Free Energy= 0.284826
Sum of electronic and zero-point Energies= -1487.173679
Sum of electronic and thermal Energies= -1487.145805
Sum of electronic and thermal Enthalpies= -1487.144861
Sum of electronic and thermal Free Energies= -1487.239330

TS-FHT-Rosmarinic-anion-O8-CH_3OO

-1 2

O	-1.83061300	1.16753800	-0.28415400
O	-3.70455400	4.17823800	0.01794200
O	-6.23956100	-2.08703800	1.64016200
O	-5.26459400	-4.04654300	0.06401000
O	-1.78921500	3.74335400	-1.05539200
O	-1.01703400	2.15030400	1.55759300
O	4.49484800	-2.22898900	-1.25914600
C	-4.04489900	1.35696900	-1.12174200
C	-3.04088200	1.92461400	-0.12824200
C	-4.38104400	-0.07978600	-0.82919800
C	-5.17059100	-0.39452200	0.27448600
C	-3.89637300	-1.10949900	-1.62171400
C	-2.80602200	3.41363000	-0.40776800
C	-5.46536900	-1.70909800	0.57330600
C	-4.19207700	-2.43478800	-1.32338400
C	-4.97378800	-2.73753700	-0.22774500
C	-0.87132800	1.39882900	0.61475800
C	2.67944600	0.04977400	0.94356100
C	0.34478300	0.63354500	0.31306500
C	1.40538900	0.73108700	1.11717300
C	2.94021700	-0.79197600	-0.13328700
C	3.67661300	0.27075400	1.91468500
C	4.17224500	-1.40370900	-0.24714500
C	4.90503800	-0.32349500	1.80403000
C	5.18491700	-1.17370100	0.72358700
H	-4.93837500	1.97356500	-1.06165000

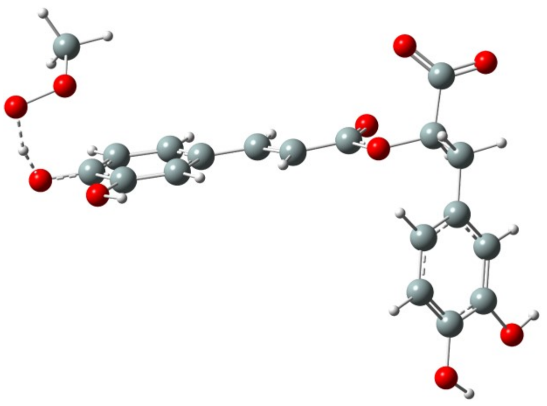

H	-3.63381900	1.44907600	-2.12476100
H	-3.40077300	1.80881600	0.88883200
H	-5.56497900	0.38726200	0.90955700
H	-3.28371900	-0.87953600	-2.47993000
H	-3.81926700	-3.24069400	-1.93626900
H	0.32325600	0.01667400	-0.57007000
H	1.33304800	1.37420600	1.98336100
H	2.19790000	-0.98084000	-0.89352700
H	3.45886600	0.92244800	2.74598400
H	5.68241700	-0.15670600	2.53244400
H	-6.52598900	-1.30864900	2.13005800
H	-5.80662800	-4.07932200	0.86079900
H	3.76027500	-2.30166800	-1.88071800
O	6.35742100	-1.77218200	0.60905200
H	6.85251900	-1.37225300	-0.29376300
O	7.20042000	-0.58865100	-1.27641800
O	6.17811600	0.29614300	-1.24254800
C	6.58156100	1.48056900	-0.54302200
H	6.99914400	1.20291400	0.41913400
H	7.31676600	2.00594500	-1.14498900
H	5.68047600	2.07140900	-0.42571200

Zero-point correction= 0.350692 (Hartree/Particle)
Thermal correction to Energy= 0.378265
Thermal correction to Enthalpy= 0.379209
Thermal correction to Gibbs Free Energy= 0.287896
Sum of electronic and zero-point Energies= -1487.172092
Sum of electronic and thermal Energies= -1487.144519
Sum of electronic and thermal Enthalpies= -1487.143575
Sum of electronic and thermal Free Energies= -1487.234887

TS-RAF-Rosmarinic-anion-C20-CH_3OO

-1 2

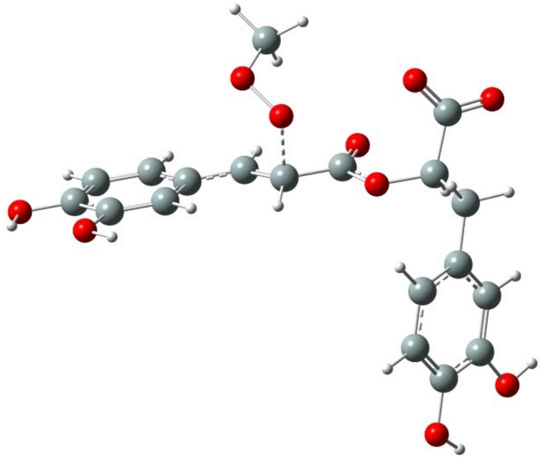

O	-1.21267600	1.01020100	-0.20651500
O	-3.09670500	3.93537000	0.54681700
O	-5.61926600	-2.43706000	1.66822100
O	-4.58854700	-4.27045300	-0.01748900
O	-1.32165500	3.65469900	-0.78716000
O	-0.24098100	1.91395200	1.59660400
O	4.96725800	-2.46648900	-1.73379300
O	6.84229700	-2.38550300	0.17543600
C	-3.48718900	1.22205900	-0.85607700
C	-2.42124100	1.72501800	0.10754600
C	-3.79136000	-0.23715800	-0.65655000
C	-4.57754200	-0.63767400	0.42112600
C	-3.27941500	-1.20428500	-1.50887500
C	-2.24386600	3.23882200	-0.05417200
C	-4.84531800	-1.97476900	0.63462500
C	-3.54472000	-2.55175000	-1.29448300
C	-4.32516100	-2.93962500	-0.22481700
C	-0.18421100	1.20640000	0.61403600
C	3.29759200	-0.32427100	0.76093200
C	1.01592700	0.46871000	0.16146800

C	2.09971900	0.40966200	1.01021400	
C	3.50652800	-1.05346100	-0.42495100	
C	4.31477600	-0.30015200	1.72713700	
C	4.68000400	-1.73602700	-0.61546800	
C	5.49396400	-0.98877300	1.53186700	
C	5.68252000	-1.71003900	0.36481800	
H	-4.37992300	1.81875800	-0.68560300	
H	-3.14534300	1.40032800	-1.87341600	
H	-2.69681000	1.50781900	1.13447500	
H	-4.99294500	0.09435500	1.10056700	
H	-2.67007100	-0.90714900	-2.34864400	
H	-3.15114500	-3.30929600	-1.95425200	
H	0.84344300	-0.29510100	-0.57682800	
H	2.07608600	1.01453100	1.90444300	
H	2.75878800	-1.08178100	-1.20324100	
H	4.16201600	0.26427400	2.63356400	
H	6.27989800	-0.97924100	2.27008200	
H	-5.94681100	-1.69795000	2.19207400	
H	-5.14136500	-4.36407300	0.76709500	
H	4.22033100	-2.45415700	-2.34227600	
H	6.81881100	-2.84071500	-0.67557500	
O	1.41582700	1.74176500	-1.26859000	
O	2.63532900	2.32386500	-1.09126000	
C	2.49216000	3.46244200	-0.24419400	
H	2.19635200	3.14421800	0.75225600	
H	3.46965100	3.93195300	-0.21610600	
H	1.75298500	4.13966900	-0.66188100	

Zero-point correction= 0.354197 (Hartree/Particle)
Thermal correction to Energy= 0.382077
Thermal correction to Enthalpy= 0.383021
Thermal correction to Gibbs Free Energy= 0.291281
Sum of electronic and zero-point Energies= -1487.170043
Sum of electronic and thermal Energies= -1487.142163
Sum of electronic and thermal Enthalpies= -1487.141219
Sum of electronic and thermal Free Energies= -1487.232959

TS-RAF-Rosmarinic-anion-C21-CH_3OO

-1 2

O	1.32892500	1.09251800	0.33477100	
O	3.46464200	3.77924200	-0.60830500	
O	4.71291800	-2.99530000	-1.87605500	
O	3.85802600	-4.59260100	0.11848600	
O	1.83445000	3.69730000	0.92263600	
O	0.33045000	2.09041300	-1.41091900	
O	-4.91803600	-2.24229400	1.96317900	
O	-6.46021600	-2.72255700	-0.18018300	
C	3.66140600	1.02992700	0.77903300	
C	2.57928500	1.66056300	-0.08685300	
C	3.72750300	-0.46388100	0.61583500	
C	4.20936000	-1.01485800	-0.56962100	
C	3.29049200	-1.31358900	1.62083900	
C	2.60775500	3.18247700	0.08682100	

C	4.25074000	-2.38424900	-0.73847500
C	3.33510900	-2.69290300	1.45508500
C	3.81389700	-3.23050600	0.27851200
C	0.26951700	1.36011000	-0.43791900
C	-3.21299700	-0.18044600	-0.56455100
C	-0.92407800	0.66185800	0.00908700
C	-2.08026000	0.73268200	-0.74817100
C	-3.48781600	-0.77851300	0.66632700
C	-4.04458100	-0.43292500	-1.64887400
C	-4.57046600	-1.62012500	0.79449600
C	-5.12715600	-1.28868300	-1.52156200
C	-5.39187700	-1.88358400	-0.30377100
H	4.60605600	1.48555400	0.49105000
H	3.46517800	1.28171300	1.81903100
H	2.73456500	1.41976500	-1.13365100
H	4.55826700	-0.37774200	-1.37107000
H	2.91332600	-0.89915300	2.54314000
H	2.99930800	-3.35935100	2.23427400
H	-0.86425900	0.09873800	0.92423300
H	-1.98808300	1.15740200	-1.73586800
H	-2.87318700	-0.59011800	1.53429700
H	-3.83822200	0.03732900	-2.59768000
H	-5.77459000	-1.50024100	-2.35765200
H	4.96626800	-2.33300500	-2.52763200
H	4.21916600	-4.79664200	-0.75188500
H	-4.29848700	-2.01221000	2.66417100
H	-6.50751700	-3.05007100	0.72605100
O	-2.91969000	2.28014900	-0.00446500
O	-2.22192900	3.41196500	-0.25397300
C	-1.58852500	3.84999700	0.94877400
H	-0.92126900	3.07172800	1.31133000
H	-1.03278200	4.74118400	0.68091500
H	-2.34393000	4.07431900	1.69550000

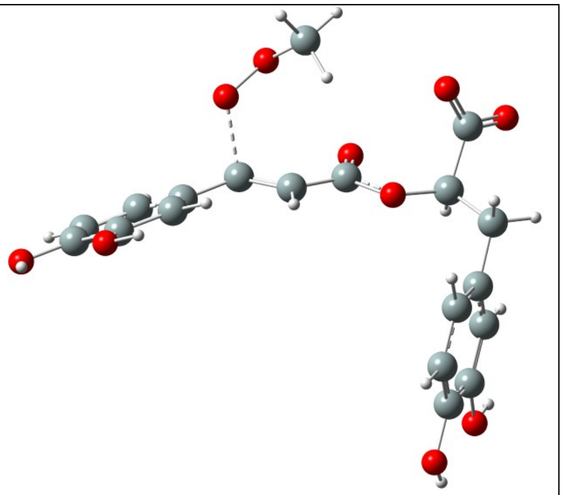

Zero-point correction= 0.353968 (Hartree/Particle)
Thermal correction to Energy= 0.381847
Thermal correction to Enthalpy= 0.382791
Thermal correction to Gibbs Free Energy= 0.291084
Sum of electronic and zero-point Energies= -1487.166719
Sum of electronic and thermal Energies= -1487.138840
Sum of electronic and thermal Enthalpies= -1487.137896
Sum of electronic and thermal Free Energies= -1487.229604

Table S3: Cartesian coordinates and thermochemistry properties of the transition states (TSs) for FHT and RAF reaction of rosmarinate mono-anion (RA^-) towards HOO$^\bullet$ and $CH_3OO^\bullet$ radicals calculated in PEA at the M05-2X/6–311++G(2df,2p) level of theory.

TS-FHT-Rosmarinic-anion-O3-HOO-PEA

-1 2			
O	-0.29018300	1.35855400	-0.31750400
O	-2.04487800	4.46349100	-0.17136100
O	-5.01719900	-1.53214600	1.72136100
O	-3.97896500	-3.60709100	0.36729800
O	-0.05019300	3.91936300	-1.06133700
O	0.61618400	2.14077400	1.58190300
O	5.96490300	-2.05458300	-1.86179800
O	7.88778900	-1.81691000	-0.03851800
C	-2.52747800	1.66505800	-1.03854700
C	-1.43288000	2.20479200	-0.12867400
C	-2.94158700	0.26739200	-0.67546800
C	-3.82223700	0.03945900	0.36114200
C	-2.40860900	-0.83919900	-1.35682900
C	-1.12495100	3.68107400	-0.48738000
C	-4.17855200	-1.26518600	0.72333600
C	-2.74237900	-2.13728100	-1.02217600
C	-3.63108600	-2.36042900	0.01711300
C	0.70334200	1.50181100	0.55993100
C	4.25008000	0.06099100	0.61739200
C	1.89641900	0.74667300	0.13207200
C	2.98895700	0.74167000	0.89426100
C	4.46275100	-0.68726100	-0.54572700
C	5.28241600	0.16104000	1.54529000
C	5.67098000	-1.30758800	-0.75750400
C	6.50098300	-0.46589700	1.33181700
C	6.70141300	-1.20188700	0.18155400
H	-3.36952900	2.34509800	-0.95003900
H	-2.16983100	1.69309400	-2.06628900
H	-1.74346400	2.15562500	0.91023400
H	-4.25181100	0.86078500	0.91721400
H	-1.71712000	-0.66389000	-2.16673100
H	-2.32932700	-2.97857100	-1.55551600
H	1.82870000	0.22633800	-0.80965000
H	2.95057200	1.30094500	1.81976100
H	3.68565800	-0.78538700	-1.28963300
H	5.12796500	0.73760000	2.44423900
H	7.30256100	-0.38890600	2.04932100
H	-4.59107000	-3.54193500	1.11383100
H	5.21626400	-2.07110400	-2.46615600
H	7.84979800	-2.27625900	-0.88486300
O	-7.06674800	-1.35198900	0.51409200
O	-6.45191900	-1.45209800	-0.68369100

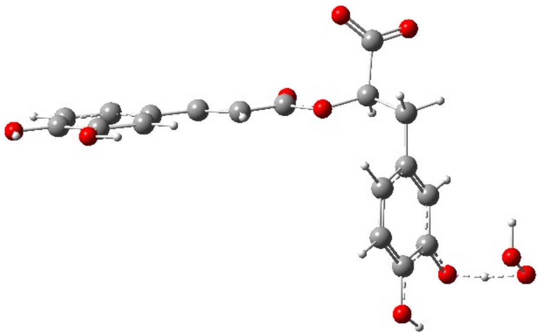

H	-6.02485700	-1.43923800	1.34135000
H	-6.20032100	-0.54450900	-0.92026400

Zero-point correction= 0.322625 (Hartree/Particle)
Thermal correction to Energy= 0.349043
Thermal correction to Enthalpy= 0.349988
Thermal correction to Gibbs Free Energy= 0.260192
Sum of electronic and zero-point Energies= -1447.855190
Sum of electronic and thermal Energies= -1447.828772
Sum of electronic and thermal Enthalpies= -1447.827828
Sum of electronic and thermal Free Energies= -1447.917624

TS-FHT-Rosmarinic-anion-O7-HOO-PEA

-1 2

O	1.43541600	1.10091700	0.19378900
O	3.31094200	4.13360800	0.07906400
O	6.16906300	-1.91763600	-1.51080100
O	5.25908400	-3.93323500	-0.00969300
O	1.22205900	3.69824400	0.79553300
O	0.74236000	1.84202400	-1.80997600
O	-5.10193000	-1.86536500	1.29184900
O	-6.86842800	-1.58607400	-0.70553700
C	3.59817000	1.35903900	1.13067200
C	2.62725900	1.89494600	0.08983900
C	4.04261400	-0.05071600	0.85161000
C	4.91426900	-0.30468900	-0.20622500
C	3.59078900	-1.12177500	1.60815700
C	2.33853400	3.39648500	0.34313700
C	5.31536700	-1.59327000	-0.49208400
C	3.99538700	-2.42153500	1.32396700
C	4.85739300	-2.66321600	0.27393800
C	0.53733500	1.25358800	-0.77463600
C	-3.07475100	0.01778100	-1.07270900
C	-0.72966700	0.57557100	-0.42781900
C	-1.75644400	0.61488900	-1.27365800
C	-3.43477500	-0.65528800	0.08171800
C	-4.02450500	0.14000800	-2.10555400
C	-4.71302700	-1.20058400	0.21296900
C	-5.29344200	-0.38893300	-2.00248300
C	-5.64919500	-1.06052100	-0.84386500
H	4.45211500	2.03129700	1.13241400
H	3.12319300	1.41181300	2.10856200
H	3.03719700	1.78669700	-0.90927400
H	5.28397400	0.51258200	-0.81200400
H	2.91357400	-0.94276600	2.42933600
H	3.64509600	-3.25566100	1.91216400
H	-0.76740300	0.07743600	0.52748300
H	-1.61403300	1.14328400	-2.20682300
H	-2.74906200	-0.77780300	0.90567500
H	-3.74278600	0.66771700	-3.00388700
H	-6.01194400	-0.28682700	-2.79954400
H	6.42172000	-1.12632200	-1.99589200
H	5.84959200	-3.90600400	-0.76977500

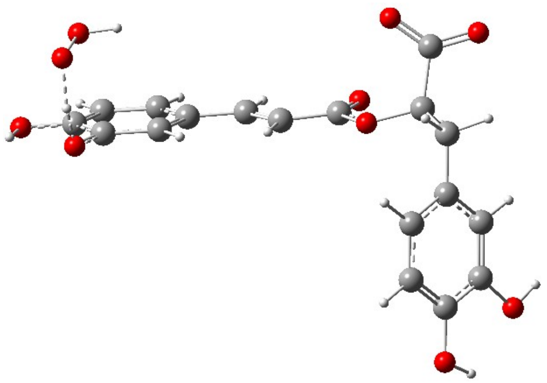

H	-5.48013300	-1.13525800	2.02276000
H	-6.90981000	-2.02001200	0.15919600
O	-5.91506000	-0.04609900	2.57962700
O	-5.91113600	0.80368900	1.52758600
H	-5.03811100	1.22856400	1.54622800

Zero-point correction= 0.322229 (Hartree/Particle)
Thermal correction to Energy= 0.348785
Thermal correction to Enthalpy= 0.349729
Thermal correction to Gibbs Free Energy= 0.259086
Sum of electronic and zero-point Energies= -1447.853608
Sum of electronic and thermal Energies= -1447.827052
Sum of electronic and thermal Enthalpies= -1447.826108
Sum of electronic and thermal Free Energies= -1447.916751

TS-RAF-Rosmarinic-anion-C20-HOO-PEA

-1 2

O	1.33844200	-1.37443800	-0.29992400
O	4.06575100	-3.54675700	0.50556900
O	4.34110900	3.20974500	1.69134900
O	3.08396200	4.66158400	-0.16664800
O	2.18920300	-3.93152900	-0.67607200
O	0.58271500	-2.08424100	1.69286900
O	-5.12940900	1.36015800	-1.96221000
O	-6.71341200	1.85500200	0.11360600
C	3.59289000	-0.93296200	-0.87996200
C	2.69054200	-1.71271700	0.06546800
C	3.46843200	0.55703100	-0.71487700
C	3.98658500	1.17405900	0.42273400
C	2.82259700	1.34309100	-1.65764400
C	2.99008900	-3.22659000	-0.04414900
C	3.85667700	2.53555900	0.60389500
C	2.69235700	2.71592200	-1.47881900
C	3.20695800	3.31751800	-0.34872200
C	0.39635300	-1.60759400	0.60065700
C	-3.08591600	-0.04733800	0.76546200
C	-0.93187500	-1.13759300	0.11521300
C	-1.84226500	-0.71285900	1.04604200
C	-3.47281000	0.33005800	-0.53223000
C	-3.95365800	0.23627200	1.82742700
C	-4.67534600	0.95957500	-0.73925600
C	-5.16339500	0.86996400	1.61400500
C	-5.53223300	1.23469800	0.33140300
H	4.61037000	-1.25400500	-0.67220200
H	3.35018900	-1.21502200	-1.90271700
H	2.85319200	-1.40390600	1.09294700
H	4.49649600	0.58535500	1.17450600
H	2.41306800	0.88059800	-2.54275500
H	2.19063500	3.32866700	-2.21178100
H	-0.96240600	-0.79616400	-0.90492500
H	-1.64319900	-0.95901600	2.07876400
H	-2.83316100	0.13241300	-1.37858200
H	-3.66636100	-0.04862100	2.82761300

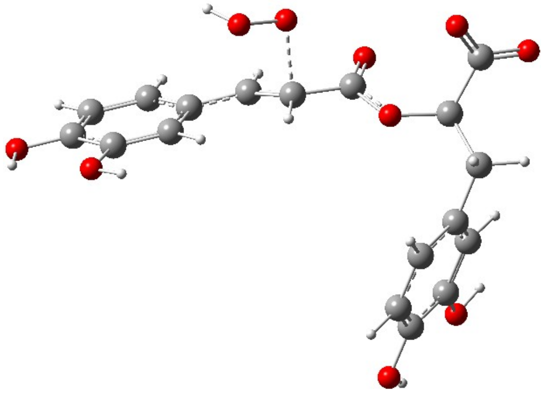

H	-5.83280600	1.08836100	2.43094300
H	4.75237300	2.59475600	2.30618700
H	3.49493000	4.89631800	0.67193100
H	-4.49565600	1.13046200	-2.64923500
H	-6.80503000	2.03309800	-0.82939600
O	-1.62937500	-2.91466400	-0.36082900
O	-2.72030600	-2.69196700	-1.15389400
H	-3.46797200	-2.71300700	-0.54171500

Zero-point correction= 0.326352 (Hartree/Particle)
Thermal correction to Energy= 0.353066
Thermal correction to Enthalpy= 0.354010
Thermal correction to Gibbs Free Energy= 0.264391
Sum of electronic and zero-point Energies= -1447.844099
Sum of electronic and thermal Energies= -1447.817385
Sum of electronic and thermal Enthalpies= -1447.816441
Sum of electronic and thermal Free Energies= -1447.906060

TS-FHT-Rosmarinic-anion-O3-CH3OO-PEA

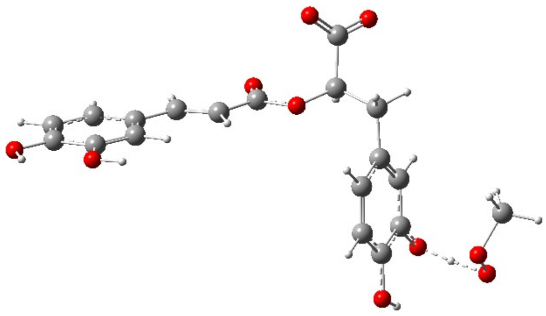

-1 2

O	0.00774700	1.42467100	-0.26912000
O	-1.66441900	4.57517000	-0.11882000
O	-4.87630400	-1.24282400	1.85433200
O	-3.83171700	-3.40025100	0.60678300
O	0.30300500	3.97446000	-1.03244800
O	0.97452900	2.19091800	1.60713800
O	6.13187700	-2.17598100	-1.91866600
O	8.09780700	-1.97893800	-0.13669000
C	-2.23646600	1.78339000	-0.94380600
C	-1.10792500	2.30257100	-0.06352000
C	-2.69457000	0.40938400	-0.54545000
C	-3.60876100	0.23891500	0.47032000
C	-2.17370500	-0.73289800	-1.18175200
C	-0.76895600	3.76737100	-0.44138500
C	-4.00932800	-1.04136200	0.87007300
C	-2.54553500	-2.00780300	-0.81042900
C	-3.46010300	-2.17304000	0.22020600
C	1.02245400	1.54463300	0.58717500
C	4.52679900	0.00128300	0.58109100
C	2.18466200	0.75369700	0.13904300
C	3.29135800	0.71902200	0.87966500
C	4.69490400	-0.75966000	-0.58116700
C	5.58037600	0.07828600	1.48705800
C	5.88117400	-1.41412700	-0.81368700
C	6.77678700	-0.58274300	1.25261200
C	6.93326000	-1.33067700	0.10326800
H	-3.05263700	2.49406600	-0.85501100
H	-1.89486900	1.77900400	-1.97754000
H	-1.39698900	2.27082300	0.98221100
H	-4.03675400	1.08971000	0.98058000
H	-1.46125900	-0.60023300	-1.98145400
H	-2.14047300	-2.87817900	-1.30166600
H	2.08344200	0.23285700	-0.79940200

H	3.28764200	1.28216600	1.80359800
H	3.90004700	-0.84156200	-1.30800300
H	5.46031800	0.66399700	2.38532000
H	7.59487600	-0.52381400	1.95299700
H	-4.46159700	-3.30915800	1.33484900
H	5.37035200	-2.17676500	-2.50694500
H	8.03025500	-2.44233500	-0.97890100
O	-6.79148900	-1.87074200	0.57942700
O	-6.15259300	-1.70993600	-0.59785200
C	-6.50204600	-0.44680300	-1.17222900
H	-6.36179000	0.33960800	-0.43803300
H	-5.83763200	-0.31245300	-2.01884000
H	-7.53845600	-0.48481100	-1.49641300
H	-5.83828800	-1.53193100	1.40746700

Zero-point correction= 0.351446 (Hartree/Particle)
Thermal correction to Energy= 0.379189
Thermal correction to Enthalpy= 0.380133
Thermal correction to Gibbs Free Energy= 0.287025
Sum of electronic and zero-point Energies= -1487.138166
Sum of electronic and thermal Energies= -1487.110424
Sum of electronic and thermal Enthalpies= -1487.109480
Sum of electronic and thermal Free Energies= -1487.202588

TS-FHT-Rosmarinic-anion-O7-CH_3OO-PEA

-1 2

O	-1.64537400	1.08638400	-0.13474100
O	-3.46889100	4.14245800	0.12285000
O	-6.47602800	-1.91698200	1.34215400
O	-5.55511600	-3.87591800	-0.22548100
O	-1.37147100	3.71108000	-0.57052800
O	-0.98179400	1.66854500	1.93042400
O	4.86713600	-1.88706100	-1.32099200
O	6.56476900	-1.86690800	0.76777500
C	-3.79117300	1.43679100	-1.08031500
C	-2.82354500	1.89594700	-0.00022500
C	-4.26555400	0.02328200	-0.88008700
C	-5.16688500	-0.26522500	0.14348800
C	-3.80975400	-1.01909900	-1.67348300
C	-2.50334400	3.40392700	-0.16201900
C	-5.59255000	-1.55939100	0.36060000
C	-4.23872800	-2.32424800	-1.45834700
C	-5.12975400	-2.60034000	-0.44154700
C	-0.76607700	1.14997200	0.86037500
C	2.80964000	-0.18954900	1.14823100
C	0.49376300	0.46991200	0.49254100
C	1.49984500	0.42099300	1.36271100
C	3.19444900	-0.75651200	-0.05211800
C	3.72674200	-0.18470100	2.22054300
C	4.46242300	-1.32295200	-0.19671500
C	4.98203400	-0.73811000	2.11029600
C	5.35881200	-1.31505300	0.90568800

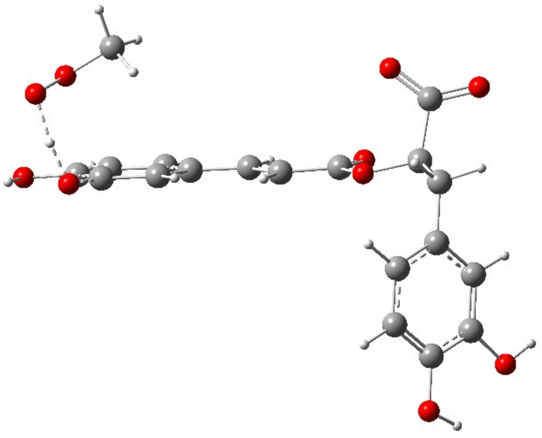

H	-4.63173600	2.12519400	-1.05627700
H	-3.30252100	1.53153100	-2.04825600
H	-3.24867200	1.73888100	0.98594300
H	-5.54000700	0.52943200	0.77668200
H	-3.10977800	-0.81346900	-2.46893400
H	-3.88484000	-3.13612100	-2.07482200
H	0.54374100	0.04269000	-0.49594900
H	1.34433900	0.87963200	2.33009900
H	2.53790600	-0.77391500	-0.90775000
H	3.42590800	0.26797700	3.15296700
H	5.67524700	-0.73227000	2.93581100
H	-6.72987300	-1.14487900	1.85668700
H	-6.16385800	-3.87436400	0.52056800
H	5.56838400	-1.16886000	-1.83082100
H	6.63602200	-2.22056100	-0.13059800
O	6.31713900	-0.18760200	-2.11710900
O	6.07274000	0.62832400	-1.06786300
C	5.14446500	1.64855000	-1.45259600
H	5.63548400	2.32355100	-2.14790600
H	4.27306200	1.19596200	-1.91394000
H	4.87746000	2.16586900	-0.53754600

Zero-point correction= 0.351400 (Hartree/Particle)
Thermal correction to Energy= 0.379179
Thermal correction to Enthalpy= 0.380123
Thermal correction to Gibbs Free Energy= 0.287162
Sum of electronic and zero-point Energies= -1487.136696
Sum of electronic and thermal Energies= -1487.108917
Sum of electronic and thermal Enthalpies= -1487.107973
Sum of electronic and thermal Free Energies= -1487.200934

TS-RAF-Rosmarinic-anion-C20-CH3OO-PEA

-1 2

O	-1.22305000	1.08553800	-0.25410500
O	-3.44625100	3.78194700	0.51458500
O	-5.19375700	-2.74814800	1.72304100
O	-4.18294000	-4.45905000	-0.06423100
O	-1.53982300	3.76133500	-0.68062400
O	-0.31685300	1.84076100	1.65188400
O	5.04948100	-2.19836600	-1.84112400
O	6.80472200	-2.35972000	0.14847300
C	-3.51464100	1.10839700	-0.85263300
C	-2.47855300	1.69826500	0.09320100
C	-3.70250100	-0.37272200	-0.66890700
C	-4.38129800	-0.85084600	0.45067100
C	-3.19122900	-1.29050700	-1.57460000
C	-2.46385700	3.24070600	-0.03452100
C	-4.53817600	-2.20684200	0.65107500
C	-3.34965100	-2.65757300	-1.37632800
C	-4.02221400	-3.12151700	-0.26419800
C	-0.22480700	1.25649800	0.59886500
C	3.27896600	-0.25951500	0.74735100

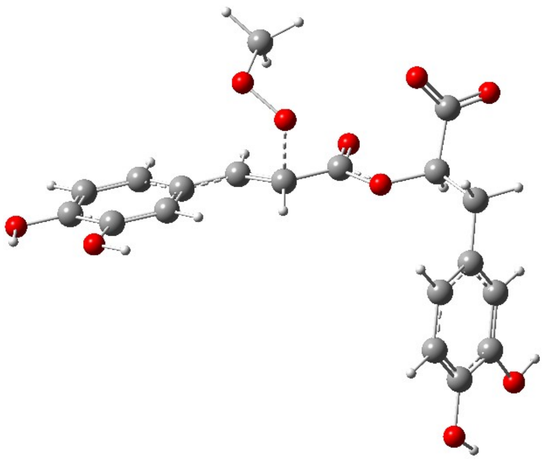

C	1.01509500	0.58021600	0.11809800	
C	2.07083400	0.47407800	0.99483000	
C	3.54106200	-0.87877500	-0.48650900	
C	4.24063300	-0.35716400	1.76020600	
C	4.70960000	-1.57344300	-0.67615200	
C	5.41463600	-1.06123600	1.56581300	
C	5.65588200	-1.67439900	0.34959900	
H	-4.44617700	1.63435600	-0.65987200	
H	-3.20955500	1.32106700	-1.87545400	
H	-2.71017600	1.44387500	1.12277500	
H	-4.79232800	-0.15705400	1.17268100	
H	-2.66007900	-0.93683100	-2.44511000	
H	-2.95267600	-3.37276800	-2.08016900	
H	0.86392300	-0.15484100	-0.65469000	
H	2.01324100	1.03360900	1.91656500	
H	2.83796900	-0.79547400	-1.30181300	
H	4.05343500	0.12357200	2.70785400	
H	6.15512000	-1.14187100	2.34585800	
H	-5.49850900	-2.05091700	2.31152600	
H	-4.66628200	-4.59049700	0.75811000	
H	4.36212500	-2.07234600	-2.50291100	
H	6.80834600	-2.70694400	-0.75071400	
O	1.52829300	1.80091600	-1.28031800	
O	2.45384400	2.71182500	-0.90063300	
C	1.82584600	3.73561500	-0.12743400	
H	1.72095100	3.39657700	0.90133300	
H	2.49049000	4.59354100	-0.17158200	
H	0.84706300	3.96123900	-0.54052500	

Zero-point correction= 0.354965 (Hartree/Particle)
Thermal correction to Energy= 0.382920
Thermal correction to Enthalpy= 0.383864
Thermal correction to Gibbs Free Energy= 0.291091
Sum of electronic and zero-point Energies= -1487.125260
Sum of electronic and thermal Energies= -1487.097305
Sum of electronic and thermal Enthalpies= -1487.096361
Sum of electronic and thermal Free Energies= -1487.189134

Table S4: Dipole moment values of rosmarinic acid anion (RA-), the studied free radicals ($HOO^{\bullet}$ and $CH_3OO^{\bullet}$) and all the transition states of FHT and RAF reactions calculated in the aqueous phase. Gibbs energies of activation values ($\Delta G^{\#}$) are also resumed for comparison.

	**Dipole moment**	$\Delta G^{\#}$
**RA^{-}**	**21.6**	
$HOO^{\bullet}$	3.0	
$CH_3OO^{\bullet}$	3.8	
**TS (RA^{-} + $HOO^{\bullet}$)**		
FHT (O3H)	22.5	18.6
FHT (O4H)	20.1	18.8
FHT (O7H)	19.6	18.8
FHT (O8H)	17.3	20.2
RAF C20	25.6	21.4
RAF C21	23.9	23.7
**TS (RA^{-} + $CH_3OO^{\bullet}$)**		
FHT (O3H)	22.4	19.1
FHT (O4H)	20.0	18.6
FHT (O7H)	19.1	18.3
FHT (O8H)	16.7	21.1
RAF C20	21.3	22.3
RAF C21	20.7	24.4

Table S5: NBO analyses calculated at the transition states (TSs) for FHT reaction between RA^- with $HOO^\bullet$ and $CH_3OO^\bullet$ radical in aqueous phase.

Position	Bond	Donor NBO (i)	Acceptor NBO (j)	E2 ($E_i - E_j$) (kcal/mol)
**$HOO^\bullet$**				
O3H	O3···H43···O41	LP(1) O3	LP*(1) H43	5.6
		LP(2) O3	LP*(1) H43	5.8
		LP(3) O3	LP*(1) H43	186.3
		LP(3) O41	LP*(1) H43	74.4
		LP(2) O41	σ*(1) O3–H43	30.3
O4H	O4···H40···O41	LP(1) O4	LP*(1) H40	5.4
		LP(2) O4	LP*(1) H40	6.6
		LP(3) O4	LP*(1) H40	188.6
		LP(3) O41	LP*(1) H40	66.0
		LP(2) O41	σ*(1) O4–H40	24.6
O7H	O7···H40···O42	LP(1) O7	LP*(1) H40	6.2
		LP(2) O7	LP*(1) H40	6.1
		LP(3) O7	LP*(1) H40	172.5
		LP(3) O42	LP*(1) H40	79.0
		LP(2) O42	σ*(1) O7–H40	19.6
O8H	O8···H41···O42	LP(1) O8	LP*(1) H41	5.2
		LP(2) O8	LP*(1) H41	8.5
		LP(3) O8	LP*(1) H41	180.6
		LP(3) O42	LP*(1) H41	72.1
		LP(2) O42	σ*(1) O8–H41	17.9
**$CH_3OO^\bullet$**				
O3H	O3···H47···O41	LP(1) O3	LP*(1) H47	6.0
		LP(2) O3	LP*(1) H47	8.0
		LP(3) O3	LP*(1) H47	174.2
		LP(3) O41	LP*(1) H47	71.2
		LP(2) O41	σ*(1) O3–H47	13.6
		LP(3) O41	σ*(1) O3–H47	31.7
O4H	O4···H40···O41	LP(1) O4	LP*(1) H40	5.8
		LP(2) O4	LP*(1) H40	6.2
		LP(3) O4	LP*(1) H40	179.1
		LP(2) O41	LP*(1) H40	73.2
		LP(3) O41	σ*(1) O4–H40	40.6
O7H	O7···H40···O41	LP(1) O7	LP*(1) H40	6.2
		LP(2) O7	LP*(1) H40	6.3
		LP(3) O7	LP*(1) H40	162.9
		LP(2) O41	LP*(1) H40	85.8
		LP(3) O41	σ*(1) O7–H40	38.2
O8H	O8···H41···O42	LP(1) O8	LP*(1) H41	5.6
		LP(2) O8	LP*(1) H41	8.6
		LP(3) O8	LP*(1) H41	170.5
		LP(3) O42	LP*(1) H41	79.8
		LP(2) O42	σ*(1) O8–H41	17.8
		LP(3) O42	σ*(1) O8–H41	32.0

Table S6: NPA charges, atomic spin densities (ASD), 1S occupancy of shifting-H, natural electron configuration (NEC) calculated at the transition states (TSs) for shifting-H, donor and acceptor of RA^{-}.

Position	Atoms	NPA charge		1s occupancy		NEC		ASD	
		$CH_3OO^{\bullet}$	$HOO^{\bullet}$	$CH_3OO^{\bullet}$	$HOO^{\bullet}$	$CH_3OO^{\bullet}$	$HOO^{\bullet}$	$CH_3OO^{\bullet}$	$HOO^{\bullet}$
O3H	H	0.3719	0.3817	0.5123	0.5145	$1s^{0.51}$	$1s^{0.51}$	-0.02135	-0.02077
	O3	-0.4317	-0.4424			$2s^{1.65}2p^{5.06}$	$2s^{1.65}2p^{5.05}$	0.12286	0.12840
	O (Rad.)	-0.5330	-0.4718			$2s^{1.80}2p^{4.61}$	$2s^{1.81}2p^{4.57}$	0.29378	0.34933
O4H	H	0.3710	0.3692	0.5123	0.5148	$1s^{0.51}$	$1s^{0.51}$	-0.02244	-0.02172
	O4	-0.4303	-0.4257			$2s^{1.65}2p^{5.05}$	$2s^{1.65}2p^{5.05}$	0.13083	0.13256
	O (Rad.)	-0.5574	-0.4803			$2s^{1.81}2p^{4.61}$	$2s^{1.81}2p^{4.58}$	0.29730	0.34840
O7H	H	0.3696	0.3779	0.5141	0.5161	$1s^{0.51}$	$1s^{0.52}$	-0.02113	-0.02064
	O7	-0.4404	-0.4387			$2s^{1.66}2p^{5.05}$	$2s^{1.65}2p^{5.04}$	0.13716	0.14291
	O (Rad.)	-0.5274	-0.4702			$2s^{1.80}2p^{4.61}$	$2s^{1.81}2p^{4.58}$	0.28294	0.33237
O8H	H	0.3760	0.3732	0.5119	0.5145	$1s^{0.51}$	$1s^{0.51}$	-0.02211	-0.02105
	O8	-0.4374	-0.4282			$2s^{1.65}2p^{5.04}$	$2s^{1.65}2p^{5.04}$	0.13190	0.13500
	O (Rad.)	-0.5490	-0.4770			$2s^{1.80}2p^{4.61}$	$2s^{1.81}2p^{4.58}$	0.29240	0.34143

Table S7: Cartesian coordinates and thermochemistry properties of optimized structures of 7 monodentate complexes types and 4 bidentate ones between the rosmarinate mono-anion (RA^-) and $[Fe(II).6H_2O]^{2+}$ ion in water calculated at the M05-2X/6–311++G(2df,2p) level of theory.

**RmAc-anion-FeII-5H2O-O2**				
1 5				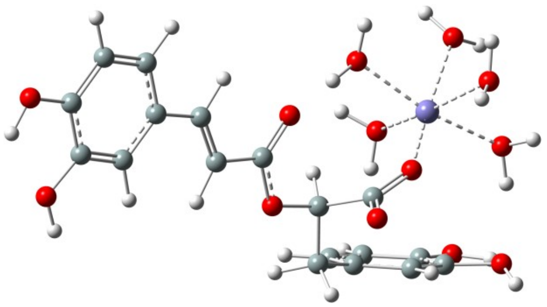
O	-1.00896600	-1.26172000	0.17973500	
O	1.72478700	0.68164500	1.24420800	
O	5.72256900	-1.15082100	1.36733500	
O	6.51535700	-1.99815300	-1.05250200	
O	0.12255100	-0.27577900	2.44705700	
O	-1.30186000	0.91158700	-0.26516800	
O	-8.27035400	-2.11787300	0.28992900	
O	-9.67546800	0.07850800	-0.19625500	
C	1.05395100	-2.40766700	0.35513600	
C	0.40386900	-1.03013300	0.21338500	
C	2.51875100	-2.36980400	0.01723800	
C	3.44749800	-1.86673200	0.92275700	
C	2.96078500	-2.76075300	-1.24137000	
C	0.74976400	-0.12923200	1.40853500	
C	4.77218700	-1.73421600	0.56035100	
C	4.29879700	-2.64647000	-1.60079600	
C	5.21339400	-2.12643500	-0.70114300	
C	-1.77149500	-0.19163800	-0.04454100	
C	-5.54461000	0.31117000	-0.19557400	
C	-3.19392200	-0.51357900	0.00380400	
C	-4.09806800	0.44772800	-0.20819200	
C	-6.18409500	-0.90916100	0.05709600	
C	-6.32055800	1.43987000	-0.44523100	
C	-7.55552500	-0.98187800	0.05598300	
C	-7.70421300	1.36732700	-0.44668800	
C	-8.32614700	0.16016800	-0.19716300	
H	0.88756500	-2.76600700	1.36847000	
H	0.54170400	-3.07808600	-0.32915200	
H	0.70838400	-0.55945200	-0.71679400	
H	3.13693000	-1.54368200	1.90517600	
H	2.25431000	-3.15318200	-1.95627100	
H	4.64279200	-2.95494700	-2.57486700	
H	-3.44747400	-1.53817700	0.21675700	
H	-3.72279300	1.44143000	-0.41260500	
H	-5.61382600	-1.80336400	0.25600300	
H	-5.83532100	2.38328800	-0.64000600	
H	-8.31128900	2.23688700	-0.63921000	
H	5.50811600	-1.26572100	2.29865400	
H	7.01453900	-1.66232600	-0.29965400	
H	-7.69153500	-2.86660900	0.45580700	
H	-9.93712400	-0.82956100	-0.00630500	
Fe	2.73456400	1.74783300	-0.08889500	

O	0.91562200	2.28513000	-1.15748500
O	3.65998100	3.31194200	-1.24910600
H	3.37347400	3.46927000	-2.15340200
H	4.62019700	3.25097300	-1.26798400
O	2.64823800	3.33727400	1.45335200
H	2.00061000	3.09146700	2.12099500
H	2.44804000	4.23641900	1.18012000
O	3.12652500	0.44715500	-1.74754000
H	3.38824000	-0.47860700	-1.65230900
H	2.51597100	0.49341200	-2.48892900
O	4.73140500	1.53633500	0.71021800
H	4.84194000	2.11758500	1.46890100
H	5.17993500	0.70599800	0.92297800
H	0.13521200	1.78567700	-0.84822300
H	0.65744900	3.21043400	-1.16864400

Zero-point correction= 0.438541 (Hartree/Particle)
Thermal correction to Energy= 0.476846
Thermal correction to Enthalpy= 0.477790
Thermal correction to Gibbs Free Energy= 0.366779
Sum of electronic and zero-point Energies= -2942.585697
Sum of electronic and thermal Energies= -2942.547392
Sum of electronic and thermal Enthalpies= -2942.546448
Sum of electronic and thermal Free Energies= -2942.657459

RmAc-anion-FeII-5H2O-O3

1 5

O	-1.70738500	-2.63736200	-0.24598400
O	-4.92024600	-3.80388400	-1.26695100
O	-2.15330500	2.59953700	0.22135000
O	-1.42018900	2.65719200	2.85523000
O	-2.92109400	-4.81721100	-1.17144100
O	-1.15697300	-1.10961600	-1.78836700
O	5.26331500	-2.67721400	1.99966700
O	6.91850500	-1.22225900	0.53095600
C	-3.80075200	-2.03479900	0.73428700
C	-3.11332500	-2.51235600	-0.53963000
C	-3.18142000	-0.77866500	1.29075000
C	-3.00494900	0.34715900	0.49372700
C	-2.73710500	-0.72387200	2.60684900
C	-3.69255600	-3.85161600	-1.03685700
C	-2.41321900	1.47798100	1.01473200
C	-2.15433300	0.41935300	3.13138900
C	-1.99253200	1.54316000	2.33733200
C	-0.84150100	-1.87052000	-0.88096100
C	2.92382800	-1.37200800	-0.52688800
C	0.51608600	-2.03137100	-0.36099300
C	1.53065800	-1.38146900	-0.94007600
C	3.38544100	-2.08017000	0.59094800
C	3.82867300	-0.61779600	-1.27011400
C	4.71319700	-2.02811400	0.93694800
C	5.16800600	-0.56369200	-0.92015100
C	5.61501300	-1.26686500	0.18053700

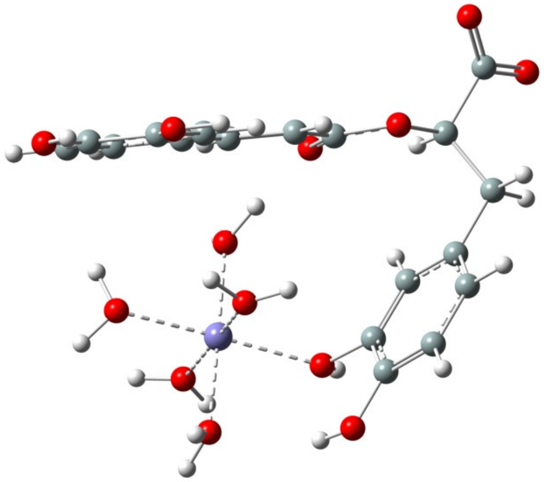

H	-4.84869500	-1.87985900	0.49319100	
H	-3.74848700	-2.82092000	1.48433500	
H	-3.23276000	-1.78764100	-1.33555300	
H	-3.30948700	0.35184400	-0.54227700	
H	-2.84772200	-1.59224900	3.23765400	
H	-1.82031400	0.45585600	4.15578000	
H	0.62878100	-2.67479400	0.49524500	
H	1.30458100	-0.77908000	-1.81033900	
H	2.71041700	-2.67190700	1.18995500	
H	3.47979300	-0.07336600	-2.13345300	
H	5.87432300	0.01620800	-1.49145500	
H	-2.93707500	2.85776900	-0.27620700	
H	-1.34726800	3.34305900	2.18079400	
H	4.60508500	-3.18875600	2.47717300	
H	7.05501200	-1.76844700	1.31362600	
Fe	-0.17255300	2.79137000	-0.68841500	
O	0.24559600	4.19288400	0.84737600	
O	1.86668500	2.95600200	-1.42581300	
H	2.38586500	3.76137100	-1.34612400	
H	2.03417600	2.59851700	-2.30312700	
O	0.65279900	1.36744500	0.64811100	
H	0.19533200	0.69726700	1.16593200	
H	1.55840000	1.07148700	0.50952500	
O	-0.81312400	4.38377300	-1.92344100	
H	-1.65568100	4.84467200	-1.95090400	
H	-0.22228600	4.81977300	-2.54355200	
O	-0.52387800	1.41850100	-2.18287700	
H	-0.72255100	0.45989200	-2.03521500	
H	-0.99957900	1.70456600	-2.96765300	
H	0.21468500	5.13721100	0.66147400	
H	0.99092500	4.03356900	1.43644200	

Zero-point correction= 0.437545 (Hartree/Particle)
Thermal correction to Energy= 0.476234
Thermal correction to Enthalpy= 0.477178
Thermal correction to Gibbs Free Energy= 0.364964
Sum of electronic and zero-point Energies= -2942.553273
Sum of electronic and thermal Energies= -2942.514585
Sum of electronic and thermal Enthalpies= -2942.513641
Sum of electronic and thermal Free Energies= -2942.625855

RmAc-anion-FeII-5H2O-O4

1 5

O	-2.66598900	-1.61126300	0.38561000	
O	-6.07984400	-2.26627900	-0.43825900	
O	-1.82283400	3.24764700	-1.74333300	
O	-0.19352700	3.62000300	0.35535800	
O	-4.62451300	-3.41425800	0.82899700	
O	-2.33328800	-2.80064600	-1.49141800	
O	4.30344000	-1.79268100	2.15640600	
O	5.96168700	-2.32788100	0.15305300	
C	-4.43172900	-0.07884200	0.79564900	
C	-3.99481600	-1.27827600	-0.04259300	

C	-3.41899700	1.03164500	0.72770000
C	-3.19810200	1.70444300	-0.47137900
C	-2.62403800	1.33210600	1.82684900
C	-4.98310300	-2.44757800	0.13327700
C	-2.16387400	2.61324100	-0.58006100
C	-1.59473300	2.26008900	1.72771200
C	-1.35221700	2.87020600	0.51736500
C	-1.92852300	-2.35038800	-0.44595600
C	1.88267100	-2.64803200	-0.48800000
C	-0.54477900	-2.44279800	0.05572500
C	0.45568700	-2.73128900	-0.78048200
C	2.37380700	-2.25551900	0.76618500
C	2.78901700	-2.91513500	-1.50984300
C	3.73106500	-2.15527300	0.97790800
C	4.15486200	-2.80578800	-1.30035600
C	4.63329300	-2.43040400	-0.06026700
H	-5.39720200	0.24388200	0.41687400
H	-4.55961800	-0.39321400	1.82875200
H	-3.95772000	-1.01429400	-1.09462600
H	-3.80260100	1.48592100	-1.34026600
H	-2.79026200	0.82113800	2.76158800
H	-0.94735100	2.47654700	2.56211200
H	-0.39092500	-2.15061500	1.08200100
H	0.19647400	-2.99713300	-1.79673000
H	1.70470900	-2.03619900	1.58437600
H	2.42071000	-3.20693600	-2.48043200
H	4.85948500	-3.01134000	-2.08950400
H	-2.49913600	3.12503900	-2.41731300
H	-0.34319900	4.34664300	-0.26328100
H	3.64476800	-1.67335300	2.84605500
H	6.12010300	-2.07678500	1.07037500
Fe	1.48928800	2.20822800	-0.17302900
O	0.87487200	2.49855500	-2.15526900
O	2.97775100	0.79025300	-0.70465000
H	3.81640300	0.97588200	-1.13499600
H	3.04181600	-0.08406800	-0.30009500
O	2.77854700	3.86991700	-0.05718900
H	2.55587100	4.70670900	0.35988500
H	3.58372300	3.99937800	-0.56510900
O	0.23641900	0.50508100	-0.04638200
H	-0.62136600	0.46940900	0.39565400
H	0.30504800	-0.27458400	-0.60881400
O	1.82926700	1.94253600	1.89305000
H	2.61901400	2.31426800	2.29780500
H	1.68549400	1.07403800	2.28215000
H	-0.04508300	2.77118400	-2.30138900
H	1.43051200	2.97224300	-2.78071700

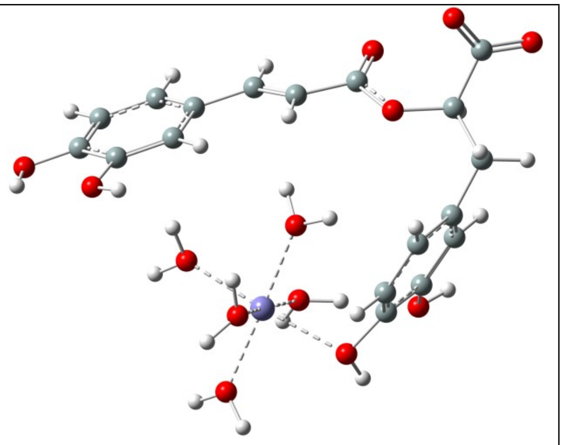

Zero-point correction= 0.438146 (Hartree/Particle)
Thermal correction to Energy= 0.476775
Thermal correction to Enthalpy= 0.477719
Thermal correction to Gibbs Free Energy= 0.366679

Sum of electronic and zero-point Energies=	-2942.548050
Sum of electronic and thermal Energies=	-2942.509420
Sum of electronic and thermal Enthalpies=	-2942.508476
Sum of electronic and thermal Free Energies=	-2942.619517

RmAc-anion-FeII-5H2O-O5

1 5

O	1.24269600	-0.29232500	0.02969100
O	2.15376200	3.05837400	-0.64052700
O	6.50230800	-2.39055700	-1.52932500
O	6.00293100	-4.36619200	0.18968100
O	0.49222700	2.18323800	0.57286300
O	0.12301200	0.40252500	-1.77469800
O	-4.46559000	-4.56007500	1.75168400
O	-6.52203700	-4.40509700	0.08698100
C	3.31998300	0.57318300	0.80603300
C	2.20820600	0.73363900	-0.22201900
C	4.04388200	-0.73781300	0.66165200
C	4.94962700	-0.91998500	-0.38151300
C	3.80926000	-1.78636600	1.53680000
C	1.56247200	2.11115300	-0.09663000
C	5.59896800	-2.12726300	-0.53949300
C	4.46371900	-3.00247000	1.38152000
C	5.35759400	-3.17759000	0.34527300
C	0.18223100	-0.29654300	-0.77583400
C	-3.15978300	-2.12264100	-0.68033900
C	-0.86196200	-1.20421600	-0.31047300
C	-2.00625300	-1.29980300	-0.99796700
C	-3.20359700	-2.96057800	0.44164700
C	-4.26248300	-2.07183300	-1.52905700
C	-4.32232300	-3.71648400	0.69205200
C	-5.39123800	-2.83417300	-1.27686500
C	-5.42632900	-3.65727100	-0.16885200
H	4.00627300	1.40535300	0.66663100
H	2.88650300	0.65507000	1.80009900
H	2.59393600	0.63023800	-1.23105000
H	5.15512300	-0.11410300	-1.07311300
H	3.11004200	-1.65766900	2.34832200
H	4.28712200	-3.82162400	2.06039000
H	-0.65752800	-1.75376100	0.59278500
H	-2.09369800	-0.70036300	-1.89466400
H	-2.36613500	-3.02527300	1.11915600
H	-4.23462100	-1.42923800	-2.39492000
H	-6.24833500	-2.80074100	-1.92949100
H	6.62150100	-1.62585600	-2.09797700
H	6.58342100	-4.30613100	-0.57640300
H	-3.68015800	-4.56180900	2.30499900
H	-6.37529700	-4.91679400	0.89071300
Fe	-1.01971400	3.54973800	0.22770600
O	-2.49671300	2.14930900	0.96209700
O	-2.56171800	5.00520900	-0.02469100
H	-3.47119500	4.78860300	0.19851600

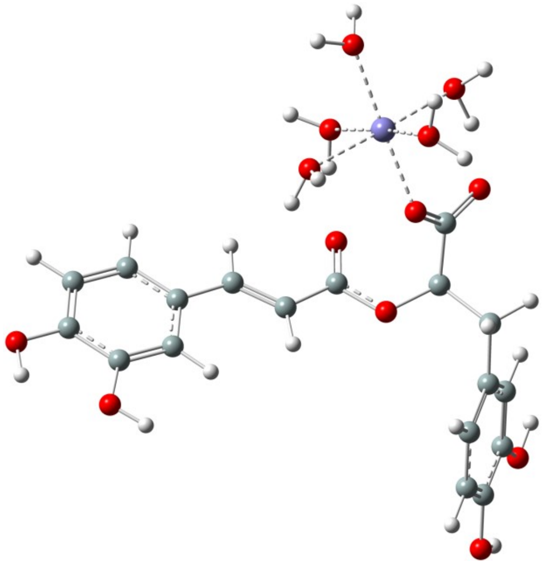

H	-2.58417700	5.57244600	-0.80037600
O	-0.84111100	4.19176100	2.27966400
H	0.02317200	4.12664000	2.69538500
H	-1.23421800	5.02163600	2.56387800
O	-1.34481200	2.72063700	-1.71281500
H	-0.86557900	1.88365900	-1.86115500
H	-2.23748800	2.60604100	-2.04859500
O	0.37983500	4.95414800	-0.51855100
H	0.60384200	5.73444400	-0.00666500
H	1.18958700	4.36988800	-0.58172700
H	-2.38360900	1.22299700	0.72529800
H	-2.60609000	2.17784000	1.91758600

Zero-point correction= 0.437354 (Hartree/Particle)
Thermal correction to Energy= 0.475907
Thermal correction to Enthalpy= 0.476851
Thermal correction to Gibbs Free Energy= 0.362965
Sum of electronic and zero-point Energies= -2942.591426
Sum of electronic and thermal Energies= -2942.552874
Sum of electronic and thermal Enthalpies= -2942.551930
Sum of electronic and thermal Free Energies= -2942.665816

RmAc-anion-FeII-5H2O-O6

1 5

O	-1.15873000	-0.35647300	-0.91153700
O	-2.63179200	2.82006000	-1.38592000
O	-5.74957800	-2.30649200	2.21966400
O	-4.97842900	-4.73411000	1.42824700
O	-1.05930900	1.76359200	-2.57024000
O	-0.01417000	1.20103100	0.19601500
O	4.80896400	-4.58942300	-0.96523600
O	6.92177200	-3.56273700	0.26039800
C	-3.43086900	-0.21856400	-1.60343400
C	-2.28741800	0.53121500	-0.93618800
C	-3.85682600	-1.42844900	-0.81719900
C	-4.62426600	-1.27128400	0.33510000
C	-3.47558700	-2.70469000	-1.19993100
C	-1.95794800	1.81180000	-1.70506200
C	-4.99604500	-2.37012000	1.08229100
C	-3.84978500	-3.81341500	-0.45016600
C	-4.60798600	-3.65096800	0.69117400
C	-0.05489500	0.09785900	-0.34709700
C	3.42492000	-1.36052700	0.13822700
C	1.04541100	-0.84662400	-0.42455500
C	2.22676900	-0.54312900	0.12657000
C	3.48349600	-2.62596700	-0.46037800
C	4.55958700	-0.85814200	0.77052800
C	4.64711500	-3.35330400	-0.41719300
C	5.73356300	-1.59158600	0.81483300
C	5.78239900	-2.83852500	0.22333200
H	-4.25691900	0.48253600	-1.70089100
H	-3.11510100	-0.50906300	-2.60266600
H	-2.54260400	0.79921300	0.08488700

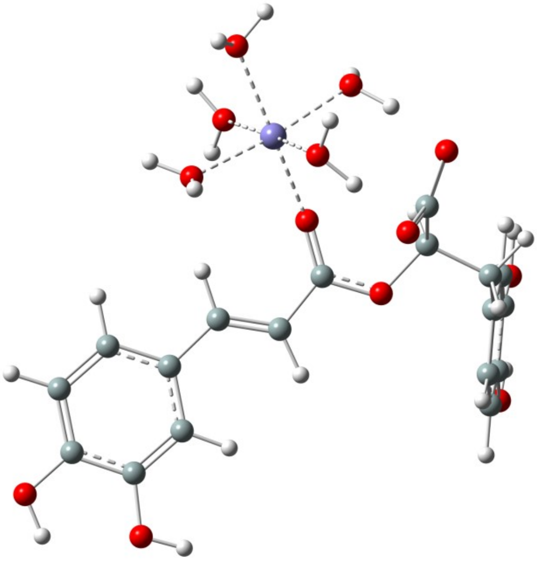

H	-4.93936800	-0.28582300	0.65018300
H	-2.88121200	-2.83959600	-2.09019600
H	-3.55832000	-4.80941100	-0.74368000
H	0.84237600	-1.77086000	-0.93812200
H	2.31559200	0.41609500	0.61587100
H	2.62309600	-3.04359900	-0.96020900
H	4.51997200	0.11650000	1.23131000
H	6.61592200	-1.21043100	1.30249400
H	-5.98132300	-1.39785400	2.42737100
H	-5.50123400	-4.43440200	2.17940400
H	4.00276600	-4.89044300	-1.39245500
H	6.78007000	-4.39992400	-0.19625200
Fe	0.55491400	3.21327800	0.48367400
O	-1.43358700	3.84535300	0.68401300
O	1.09480800	5.27332500	0.96687800
H	0.33842500	5.86044900	1.05833200
H	1.75854300	5.74957300	0.46055700
O	0.70310100	2.81444700	2.56309500
H	0.84255200	1.93504600	2.92404700
H	1.06042500	3.45206100	3.18669000
O	0.52909300	3.58179800	-1.59849400
H	-0.05460000	2.95600900	-2.11204100
H	0.34725800	4.47228000	-1.90733400
O	2.63760700	2.84418700	0.26926100
H	3.29344100	3.12022300	0.91603600
H	3.00422300	3.00031300	-0.60657500
H	-2.00666700	3.49791200	-0.05466400
H	-1.86289600	3.63805000	1.51811200

Zero-point correction= 0.436181 (Hartree/Particle)
Thermal correction to Energy= 0.474100
Thermal correction to Enthalpy= 0.475044
Thermal correction to Gibbs Free Energy= 0.362847
Sum of electronic and zero-point Energies= -2942.582824
Sum of electronic and thermal Energies= -2942.544905
Sum of electronic and thermal Enthalpies= -2942.543961
Sum of electronic and thermal Free Energies= -2942.656159

RmAc-anion-FeII-5H2O-O7

1 5

O	-3.21385100	-1.29550800	0.52259800
O	-6.23619400	-3.16219000	0.81302700
O	-5.99963700	3.11726900	-2.14966300
O	-4.14311000	4.69181100	-1.05793700
O	-4.23342000	-3.44850600	1.78393100
O	-2.98969700	-2.81314300	-1.11300800
O	3.95897400	-0.63992000	0.95132100
O	5.41758600	-2.20848500	-0.64896600
C	-5.24131900	-0.33759600	1.29345800
C	-4.63579600	-1.47790200	0.48605900
C	-4.95821700	1.01214900	0.69207700
C	-5.64410500	1.42061400	-0.45047700
C	-4.00163800	1.85778200	1.23233600

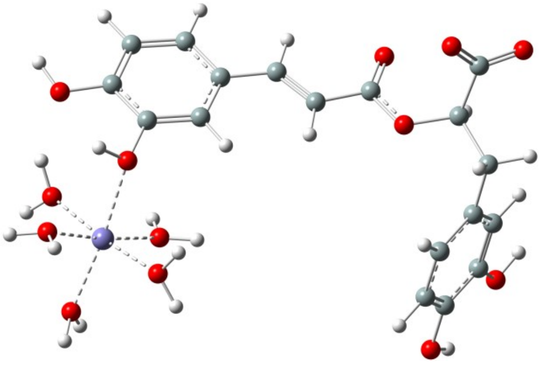

C	-5.05889700	-2.83649000	1.08103700	
C	-5.37184600	2.64183600	-1.03272500	
C	-3.72657700	3.08965400	0.64996200	
C	-4.40755900	3.48487800	-0.48293700	
C	-2.49899900	-2.06187400	-0.30033800	
C	1.27757600	-2.45050300	-0.76363000	
C	-1.05460400	-1.85038200	-0.10144000	
C	-0.17793300	-2.53883500	-0.83035700	
C	1.93926700	-1.57748500	0.10275200	
C	2.03755100	-3.27363500	-1.59117400	
C	3.30986800	-1.53534500	0.11284000	
C	3.42186900	-3.23116100	-1.57530500	
C	4.06329900	-2.35150900	-0.72568500	
H	-6.31246300	-0.51641100	1.33940800	
H	-4.84787800	-0.38352600	2.30699900	
H	-4.96810000	-1.43369500	-0.54628400	
H	-6.40001200	0.78274700	-0.88851800	
H	-3.46287500	1.55566200	2.11703900	
H	-2.98486500	3.75155500	1.06843800	
H	-0.77174300	-1.12983900	0.64805500	
H	-0.57549400	-3.24067900	-1.55098100	
H	1.40239800	-0.93123100	0.77808900	
H	1.53817600	-3.95664000	-2.25978500	
H	4.00204400	-3.86897800	-2.22377700	
H	-6.64692400	2.48565600	-2.47275600	
H	-4.71163100	4.79876100	-1.82777100	
H	4.74911500	-1.04457700	1.33476000	
H	5.86735200	-2.89743800	-1.14883500	
Fe	4.50046200	1.42076700	0.37375400	
O	2.83399300	1.61457700	-0.89555800	
O	5.23933000	3.33304900	-0.12172100	
H	4.88723400	3.81133800	-0.87784100	
H	5.51703300	3.98340600	0.52948000	
O	3.36460900	2.11217000	2.00136400	
H	2.93198000	1.50535400	2.60882800	
H	2.91056900	2.95777500	2.05345600	
O	5.53613700	0.54584300	-1.23565100	
H	5.81074700	-0.38236600	-1.21204000	
H	6.08849800	1.02145300	-1.86103200	
O	6.14048800	1.02057800	1.65802900	
H	6.10909600	1.30459200	2.57678100	
H	7.05112600	1.10648200	1.36014100	
H	2.85551700	1.27403500	-1.79461300	
H	1.91577900	1.66109100	-0.61570100	

Zero-point correction= 0.436142 (Hartree/Particle)
Thermal correction to Energy= 0.475251
Thermal correction to Enthalpy= 0.476195
Thermal correction to Gibbs Free Energy= 0.360994
Sum of electronic and zero-point Energies= -2942.546330
Sum of electronic and thermal Energies= -2942.507221
Sum of electronic and thermal Enthalpies= -2942.506277

Sum of electronic and thermal Free Energies= -2942.621479				
**RmAc-anion-FeII-5H2O-O8**				
1 5				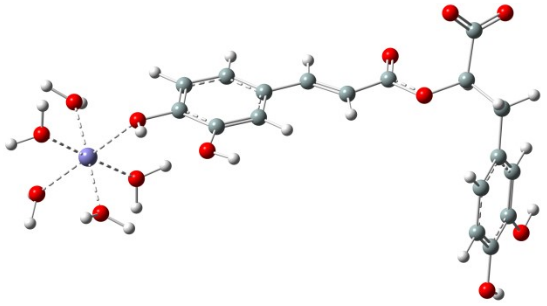
O	-3.87450000	-1.24499300	0.28419600	
O	-6.40185100	-3.61974600	-0.52444400	
O	-7.06086500	3.18411100	-1.84622400	
O	-5.82960600	4.69983100	-0.02712400	
O	-4.59438600	-3.78845200	0.79554400	
O	-3.04961300	-2.22630700	-1.55563200	
O	2.93613500	0.09187700	2.56571700	
O	4.85850700	-0.64837400	0.93704800	
C	-6.16370200	-0.88991800	0.79291200	
C	-5.19466800	-1.65230700	-0.10028300	
C	-6.08747500	0.59961600	0.59578700	
C	-6.63481100	1.17802100	-0.54804100	
C	-5.45675500	1.41866000	1.51951500	
C	-5.39767100	-3.17186400	0.07099800	
C	-6.54774300	2.53950800	-0.75576000	
C	-5.36876300	2.79074500	1.31452400	
C	-5.91116400	3.35505100	0.17837400	
C	-2.88016600	-1.65027000	-0.50450700	
C	0.91842100	-1.33975400	-0.16180400	
C	-1.56987000	-1.28838300	0.06536500	
C	-0.45374500	-1.60546200	-0.58766900	
C	1.21768000	-0.72160300	1.05621000	
C	1.95774100	-1.72771700	-1.00120400	
C	2.52788800	-0.49778800	1.41228100	
C	3.28126500	-1.51224500	-0.64236800	
C	3.55336400	-0.89480000	0.55614500	
H	-7.16138800	-1.25342800	0.55996300	
H	-5.94749000	-1.13895600	1.82988900	
H	-5.35198700	-1.39594400	-1.14296600	
H	-7.13767100	0.56289600	-1.28200400	
H	-5.02821500	0.98526800	2.40991800	
H	-4.88116400	3.43203400	2.03171800	
H	-1.58239800	-0.77722000	1.01370300	
H	-0.56148300	-2.11774100	-1.53400300	
H	0.43356900	-0.41392300	1.72990100	
H	1.73271900	-2.21123700	-1.93806500	
H	4.09482600	-1.83248600	-1.27229400	
H	-7.48390800	2.56196800	-2.44325900	
H	-6.26320000	4.91218100	-0.86025900	
H	2.19649700	0.27687500	3.15118600	
H	4.86618600	-0.41524300	1.87725600	
Fe	6.22594800	0.58410400	-0.23776600	
O	5.99217000	2.12144500	1.18858100	
O	7.64654500	1.80349600	-1.24944300	
H	7.89664000	2.64327700	-0.85285100	
H	7.65184900	1.92394600	-2.20333600	
O	7.76113800	-0.25371000	0.93688200	
H	7.76867000	-1.15730900	1.26509800	

H	8.67259800	0.04726300	0.88272400
O	4.63136800	1.38297300	-1.36144700
H	3.74516300	1.01169700	-1.41833600
H	4.59280500	2.29393600	-1.66692700
O	6.48297000	-0.90942200	-1.70626100
H	7.31070300	-1.39507300	-1.77595700
H	6.15728300	-0.76718000	-2.60067700
H	5.15555300	2.55911100	1.37184100
H	6.52613300	2.15978500	1.98764400

Zero-point correction= 0.435975 (Hartree/Particle)
Thermal correction to Energy= 0.476207
Thermal correction to Enthalpy= 0.477151
Thermal correction to Gibbs Free Energy= 0.357841
Sum of electronic and zero-point Energies= -2942.543205
Sum of electronic and thermal Energies= -2942.502973
Sum of electronic and thermal Enthalpies= -2942.502029
Sum of electronic and thermal Free Energies= -2942.621339

RmAc-anion-FeII-4H2O-site1

1 5

O	-0.84705100	0.21518400	1.28429100
O	-3.56951700	-0.85357000	2.86882400
O	-2.98848600	4.48401000	-2.24936500
O	-0.81637500	5.80970400	-1.44978400
O	-2.45871200	-2.13992700	1.43388800
O	-0.57136700	-1.01942800	-0.56657600
O	6.39334400	1.20085700	1.55523400
O	7.77134500	-0.10906900	-0.28885400
C	-2.77028800	1.54405600	1.71096800
C	-2.28853600	0.21950200	1.15702100
C	-2.26212500	2.70124000	0.89381500
C	-2.90039200	3.04525900	-0.29624200
C	-1.13684000	3.41423900	1.27892100
C	-2.83757300	-1.00770100	1.90429800
C	-2.41984800	4.07852300	-1.07524300
C	-0.65049000	4.45447300	0.49654600
C	-1.28752300	4.78906000	-0.68107000
C	-0.10068900	-0.34922800	0.35226700
C	3.65239000	-0.46164400	-0.25783900
C	1.31497600	-0.08037600	0.54084300
C	2.21160500	-0.62310100	-0.29210200
C	4.29968400	0.33814400	0.69357900
C	4.41615500	-1.13165600	-1.21043800
C	5.66780900	0.45191300	0.67940800
C	5.79620500	-1.01747100	-1.22513600
C	6.42631600	-0.22799900	-0.28347200
H	-3.85661500	1.51913300	1.70764100
H	-2.44715500	1.62968500	2.74461800
H	-2.53951800	0.12595700	0.10396100
H	-3.78237000	2.50691200	-0.61629000
H	-0.63233400	3.15800900	2.19754700
H	0.22241900	5.01435700	0.79276300

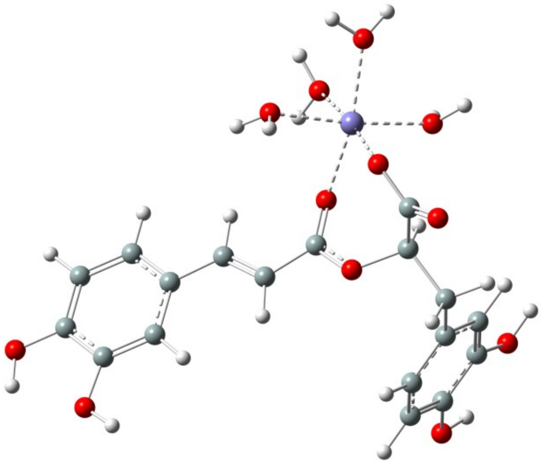

H	1.57352000	0.56024600	1.36654600
H	1.83213200	-1.25437700	-1.08396700
H	3.73732500	0.87272400	1.44349700
H	3.92322000	-1.74788100	-1.94565000
H	6.39430000	-1.53287700	-1.95876900
H	-3.77243900	3.96583500	-2.44717500
H	-1.38305200	5.90287300	-2.22282600
H	5.82652000	1.64281400	2.19272000
H	8.04070000	0.47635500	0.42823300
Fe	-1.90356500	-2.70902600	-0.39483100
O	-2.91811200	-4.57262500	-0.56593900
H	-2.44537900	-5.37860300	-0.34108500
H	-3.58250500	-4.79501500	-1.22290000
O	-3.51474700	-1.68013500	-1.40780100
H	-3.40971900	-1.50212900	-2.34661100
H	-4.43857300	-1.90103700	-1.26203200
O	-0.32388100	-3.93517900	0.48648800
H	0.59222500	-3.79939400	0.22923900
H	-0.34829800	-3.90725900	1.44785900
O	-1.09757800	-3.08106000	-2.34993000
H	-0.34343300	-2.52967400	-2.57864900
H	-0.92422000	-3.96548600	-2.68364500

Zero-point correction= 0.411609 (Hartree/Particle)
Thermal correction to Energy= 0.449375
Thermal correction to Enthalpy= 0.450319
Thermal correction to Gibbs Free Energy= 0.336903
Sum of electronic and zero-point Energies= -2866.139185
Sum of electronic and thermal Energies= -2866.101419
Sum of electronic and thermal Enthalpies= -2866.100475
Sum of electronic and thermal Free Energies= -2866.213892

RmAc-anion-FeII-4H2O-site2

1 5

O	-0.89052300	0.19675900	1.24733100
O	-2.37025200	-2.21554200	1.40232300
O	-3.19606800	4.45651700	-2.21713700
O	-1.05885700	5.83090100	-1.40520200
O	-3.63753900	-0.99215500	2.76008500
O	-0.56506800	-0.99837000	-0.62060700
O	6.31321800	1.40611300	1.57302100
O	7.74583700	0.13793700	-0.25917400
C	-2.86302500	1.44799500	1.68320000
C	-2.32996400	0.14894600	1.11516500
C	-2.39431200	2.63444100	0.88484400
C	-3.05122000	2.98277000	-0.29367100
C	-1.28756700	3.37268100	1.27636800
C	-2.84229000	-1.10612300	1.84129500
C	-2.60708400	4.04510900	-1.05514000
C	-0.83811700	4.44223100	0.51183900
C	-1.49361200	4.78114600	-0.65442400
C	-0.12050900	-0.32803000	0.31109200
C	3.63855500	-0.33427000	-0.26546200

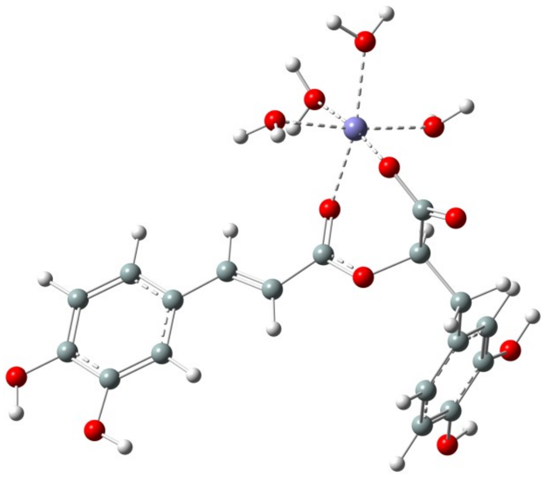

C	1.28478500	-0.01852800	0.51246100	
C	2.20319700	-0.53593100	-0.31314700	
C	4.25404600	0.48356100	0.69168800	
C	4.43007800	-0.98188900	-1.21089400	
C	5.61829500	0.63677600	0.69007400	
C	5.80636100	-0.82761800	-1.21317000	
C	6.40488000	-0.02036500	-0.26593000	
H	-3.94738200	1.38299100	1.67423400	
H	-2.54791000	1.53159900	2.71969300	
H	-2.57407600	0.05906900	0.05990100	
H	-3.91965800	2.42537400	-0.61813100	
H	-0.76900100	3.11346900	2.18628200	
H	0.01983900	5.02199100	0.81346000	
H	1.51861400	0.62586300	1.34257900	
H	1.84821900	-1.17758600	-1.10832400	
H	3.67038500	1.00176000	1.43669300	
H	3.96195300	-1.61221600	-1.95035500	
H	6.42581000	-1.32523100	-1.94134900	
H	-3.96724300	3.92085400	-2.41843800	
H	-1.63536000	5.92460100	-2.17086200	
H	5.72721800	1.83024900	2.20534300	
H	7.99120600	0.73071200	0.46049400	
Fe	-1.74938100	-2.79360300	-0.39914900	
O	-2.62560300	-4.72798200	-0.50275800	
O	-0.68802400	-3.28302400	-2.19532000	
H	-1.05860900	-3.87395400	-2.85660400	
H	-0.30424600	-2.53173800	-2.65731100	
O	-3.35212800	-1.89301000	-1.51219600	
H	-4.24755100	-2.23623100	-1.56637700	
H	-3.19223600	-1.37893600	-2.30793900	
O	-0.13198500	-3.88269800	0.61408200	
H	-0.10337500	-3.69954500	1.55780300	
H	0.76529300	-3.78939300	0.28253800	
H	-2.08917900	-5.47532500	-0.22474300	
H	-3.30786900	-5.05568700	-1.09343200	

Zero-point correction= 0.411548 (Hartree/Particle)
Thermal correction to Energy= 0.449364
Thermal correction to Enthalpy= 0.450308
Thermal correction to Gibbs Free Energy= 0.336350
Sum of electronic and zero-point Energies= -2866.139277
Sum of electronic and thermal Energies= -2866.101461
Sum of electronic and thermal Enthalpies= -2866.100517
Sum of electronic and thermal Free Energies= -2866.214475

RmAc-anion-FeII-4H2O-site3

1 5

O	-1.51925700	2.13675300	0.26964700	
O	-0.66464200	5.45548300	-0.71259000	
O	4.02584600	0.36444500	-0.66331800	
O	3.54835400	-1.45363800	1.11977500	
O	-2.50056700	4.64516600	0.29281400	
O	-2.34472800	2.20544700	-1.81393100	

O	-6.63370600	-2.76782300	2.06908400
O	-8.20968000	-3.54880000	0.08074900
C	0.41684000	3.25783600	1.04854300
C	-0.62309700	3.20279600	-0.06240000
C	1.24618200	2.00564300	1.11443600
C	2.25889800	1.79959700	0.18080800
C	1.00478400	1.02949200	2.07007800
C	-1.35111800	4.55867900	-0.17587600
C	2.99983500	0.64093000	0.21803300
C	1.75311200	-0.13984000	2.10648000
C	2.74996400	-0.32697500	1.17645900
C	-2.37671000	1.76636600	-0.68542100
C	-5.26500600	-0.73815700	-0.68341000
C	-3.32496400	0.76039300	-0.18613100
C	-4.24683400	0.25233800	-1.00573800
C	-5.42236900	-1.26699700	0.60337500
C	-6.11471400	-1.17290700	-1.69631500
C	-6.40135500	-2.19870400	0.85224300
C	-7.10074800	-2.11486300	-1.44668000
C	-7.24831000	-2.63065800	-0.17502200
H	1.04881600	4.11951700	0.85242200
H	-0.09250500	3.41768200	1.99626200
H	-0.15155200	2.98507300	-1.01546300
H	2.47131900	2.54540800	-0.57176600
H	0.22237100	1.17846600	2.79691400
H	1.56191100	-0.89791700	2.85025900
H	-3.22763300	0.47727200	0.84881400
H	-4.24697900	0.60914800	-2.02708800
H	-4.78498600	-0.95043200	1.41474500
H	-6.00201500	-0.76950100	-2.69034100
H	-7.76122800	-2.45507200	-2.22771400
H	4.14417200	1.05977800	-1.31865900
H	3.37707600	-2.06017200	1.84751600
H	-6.03891200	-2.41511900	2.73592600
H	-8.17397500	-3.79064700	1.01296800
Fe	5.16162800	-1.45392700	-0.33089700
O	6.42116100	-1.35040600	-2.02003300
H	6.66184100	-0.54022400	-2.47693600
H	6.84954600	-2.08257800	-2.47132300
O	6.34480500	-0.27995100	0.94191100
H	7.09171300	-0.65699200	1.41587400
H	6.53237700	0.64835300	0.77691200
O	3.88609700	-2.60267700	-1.53121400
H	2.99316400	-2.82044400	-1.24837000
H	3.87539500	-2.49347300	-2.48672700
O	6.24454200	-3.08085700	0.46988300
H	7.10657900	-3.36390900	0.15305200
H	5.82901600	-3.83707600	0.89307000

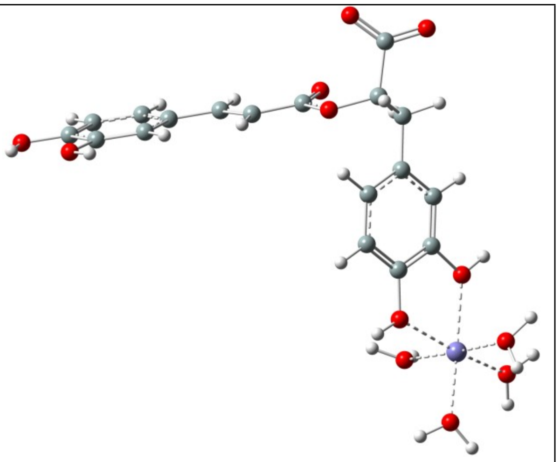

Zero-point correction= 0.410974 (Hartree/Particle)
Thermal correction to Energy= 0.449277
Thermal correction to Enthalpy= 0.450221

Thermal correction to Gibbs Free Energy= 0.335032
Sum of electronic and zero-point Energies= -2866.104524
Sum of electronic and thermal Energies= -2866.066221
Sum of electronic and thermal Enthalpies= -2866.065277
Sum of electronic and thermal Free Energies= -2866.180466

RmAc-anion-FeII-4H2O-site4

1 5

O	3.40615400	-1.17431500	-0.44543300
O	6.00040700	-3.59002500	-0.79725800
O	7.14165900	2.78566900	1.75538200
O	5.53309000	4.61860100	0.67173300
O	3.92335600	-3.52852500	-1.64511400
O	3.01888800	-2.55455200	1.27864300
O	-3.70657100	0.49607400	-0.62267900
O	-5.20600500	-0.69322000	1.08961300
C	5.51568300	-0.65054600	-1.39367100
C	4.77032900	-1.61593600	-0.48229900
C	5.52611100	0.75833100	-0.86595200
C	6.35513000	1.09340300	0.20307100
C	4.70506800	1.73725900	-1.40413800
C	4.89188400	-3.05660800	-1.02085600
C	6.35652900	2.37502500	0.71479400
C	4.70460200	3.02957700	-0.89209600
C	5.52727700	3.35258700	0.16734800
C	2.61900100	-1.76899300	0.44930200
C	-1.14494600	-1.52140500	1.08892800
C	1.21935000	-1.32963900	0.30085900
C	0.28272000	-1.83033000	1.10332500
C	-1.70873000	-0.61802600	0.18281200
C	-1.96885900	-2.15595400	2.01309200
C	-3.05786300	-0.37458200	0.22357600
C	-3.33289000	-1.90884400	2.04928800
C	-3.86907900	-1.01724700	1.15044000
H	6.53084200	-1.02678500	-1.49096000
H	5.05121100	-0.67555200	-2.37741300
H	5.17634800	-1.58497700	0.52366200
H	7.00945400	0.35007800	0.63806300
H	4.05787600	1.49323100	-2.23234000
H	4.06939000	3.79490000	-1.30941500
H	1.02016100	-0.61276400	-0.47839900
H	0.59994700	-2.54652800	1.84892000
H	-1.10289800	-0.10892400	-0.54974200
H	-1.53932600	-2.85371400	2.71358600
H	-3.96783400	-2.40341700	2.76707800
H	7.68687100	2.06392000	2.07838100
H	6.16721700	4.66019200	1.39518100
H	-3.10852300	0.93865100	-1.23382800
H	-5.73809000	-1.17678000	1.73115600
Fe	-5.86204700	0.73102400	-0.40115300
O	-5.71302700	2.23690700	1.04248800
O	-7.98188100	0.72355000	0.05759100

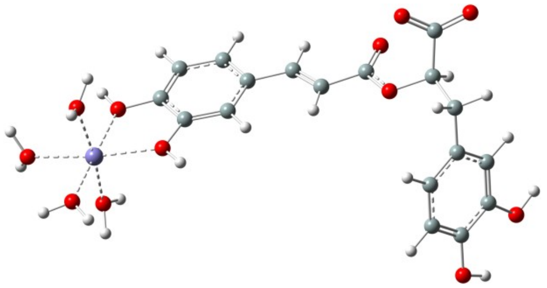

H	-8.26246300	1.03915300	0.92137900	
H	-8.48712800	-0.07225100	-0.13213000	
O	-6.46088200	2.08970700	-1.88690400	
H	-5.91972600	2.65176600	-2.44689600	
H	-7.36087800	2.42742500	-1.89038300	
O	-6.28183300	-0.82286200	-1.73580500	
H	-6.37848400	-0.61827700	-2.67105700	
H	-5.93642300	-1.71768500	-1.66308100	
H	-5.24790400	2.12578000	1.87715100	
H	-5.64116100	3.16144900	0.78607500	

Zero-point correction=	0.410938 (Hartree/Particle)
Thermal correction to Energy=	0.448907
Thermal correction to Enthalpy=	0.449851
Thermal correction to Gibbs Free Energy=	0.336109
Sum of electronic and zero-point Energies=	-2866.101618
Sum of electronic and thermal Energies=	-2866.063649
Sum of electronic and thermal Enthalpies=	-2866.062705
Sum of electronic and thermal Free Energies=	-2866.176447

Table S8: Cartesian coordinates and thermochemistry properties of optimized structures of 7 monodentate complexes types and 4 bidentate ones between the rosmarinate mono-anion (RA^-) and $[Fe(III).6H_2O]^{3+}$ ion in water calculated at the M05-2X/6–311++G(2df,2p) level of theory.

**RmAc-anion-FeIII-5H2O-O2**				
2 6				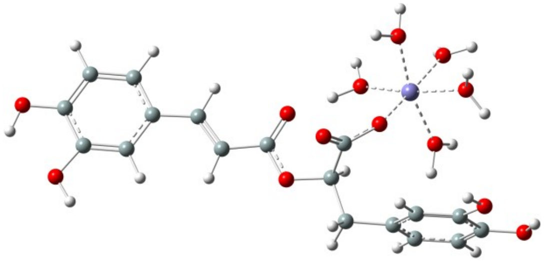
O	-0.94653700	-1.34149500	-0.11730700	
O	1.85067700	0.53741700	0.90648000	
O	5.69162500	-1.96625100	1.74331400	
O	6.73620400	-1.94210600	-0.70693700	
O	0.31659100	-0.46368300	2.16264800	
O	-1.26944900	0.86450000	-0.31250600	
O	-8.19774700	-2.25222200	0.14107400	
O	-9.61784100	-0.01772500	0.02878000	
C	1.13891500	-2.46802100	-0.10936800	
C	0.45909500	-1.10196900	-0.13508400	
C	2.63138300	-2.34991100	-0.26407900	
C	3.45280100	-2.22904700	0.85362400	
C	3.20954900	-2.33498000	-1.52857400	
C	0.85160200	-0.29232500	1.09910600	
C	4.81927500	-2.09155900	0.70766900	
C	4.58746500	-2.19797900	-1.67813800	
C	5.39508600	-2.07336200	-0.56390500	
C	-1.72846200	-0.26469800	-0.19877300	
C	-5.49461400	0.23335600	-0.13398300	
C	-3.13927300	-0.59791300	-0.13174100	
C	-4.05309900	0.37888100	-0.19154300	
C	-6.12238200	-1.01452600	-0.02072900	
C	-6.27967300	1.38241000	-0.19215200	
C	-7.49175300	-1.09293600	0.03235000	
C	-7.66121700	1.30394600	-0.13748100	
C	-8.27164900	0.07024200	-0.02515700	
H	0.88161800	-2.95908000	0.82569900	
H	0.72294900	-3.04997500	-0.92680500	
H	0.72353000	-0.56184600	-1.04019100	
H	3.02694900	-2.24391200	1.84729100	
H	2.58884800	-2.44633200	-2.40457200	
H	5.04282000	-2.19463500	-2.65551000	
H	-3.38074800	-1.64177900	-0.02875500	
H	-3.68740300	1.39189400	-0.29081000	
H	-5.54478800	-1.92476600	0.02536900	
H	-5.80237700	2.34563500	-0.27966900	
H	-8.27593600	2.18812400	-0.18091200	
H	5.23885900	-2.01540500	2.58948800	
H	7.14239500	-1.88317900	0.16488300	
H	-7.61433800	-3.01511300	0.16884000	
H	-9.87230200	-0.94449600	0.10499300	
Fe	2.52788000	1.97905800	-0.02477700	
O	0.80098600	2.25860100	-1.01562900	
O	3.23646700	3.61357800	-1.06883400	

H	2.95258200	3.94299400	-1.92846400
H	4.12333100	3.94871300	-0.89141100
O	2.06713200	3.30940800	1.46690700
H	1.45938100	3.04747200	2.16800400
H	1.97085900	4.25544500	1.30932400
O	3.37813400	0.79961700	-1.43787600
H	3.41579100	-0.17374300	-1.39906400
H	3.80529400	1.10023500	-2.24734700
O	4.38765200	2.01943700	0.85307500
H	4.49673400	2.27852800	1.77505200
H	5.07376200	1.38305800	0.62310000
H	0.00920000	1.70727500	-0.75618100
H	0.48865300	3.09910800	-1.36590900

Zero-point correction= 0.439598 (Hartree/Particle)
Thermal correction to Energy= 0.477767
Thermal correction to Enthalpy= 0.478712
Thermal correction to Gibbs Free Energy= 0.366595
Sum of electronic and zero-point Energies= -2942.361448
Sum of electronic and thermal Energies= -2942.323279
Sum of electronic and thermal Enthalpies= -2942.322335
Sum of electronic and thermal Free Energies= -2942.434452

RmAc-anion-FeIII-5H2O-O3

2 6

O	-1.30602000	-2.77487400	-0.24478500
O	-4.32453700	-4.32549700	-1.34247600
O	-2.37974300	2.37848700	0.17102800
O	-2.01056600	2.58008700	2.89870000
O	-2.21918700	-5.08730200	-1.17879800
O	-0.93554700	-1.13484300	-1.70377400
O	5.58613700	-2.07749300	2.10464600
O	7.14474500	-0.60265000	0.55350300
C	-3.49252600	-2.42546600	0.66623700
C	-2.71465500	-2.81875400	-0.58107500
C	-3.09044400	-1.08725800	1.23147700
C	-2.97358100	0.03792600	0.42578400
C	-2.81515200	-0.94861300	2.58980800
C	-3.10817500	-4.22579800	-1.07839800
C	-2.59803800	1.23927600	0.98919100
C	-2.46846000	0.26829100	3.14938500
C	-2.36617700	1.39634400	2.34846300
C	-0.52577100	-1.89135900	-0.80730300
C	3.16592100	-1.04131600	-0.46953800
C	0.82670500	-1.89706900	-0.28344400
C	1.78014000	-1.16337900	-0.87482600
C	3.67208200	-1.66501200	0.67955500
C	4.02147200	-0.27213800	-1.25574100
C	4.99463200	-1.51765300	1.01451500
C	5.35553900	-0.12261700	-0.91764600
C	5.84689200	-0.74256100	0.21461700
H	-4.54401200	-2.42573400	0.39298000
H	-3.35276700	-3.18810200	1.42873200

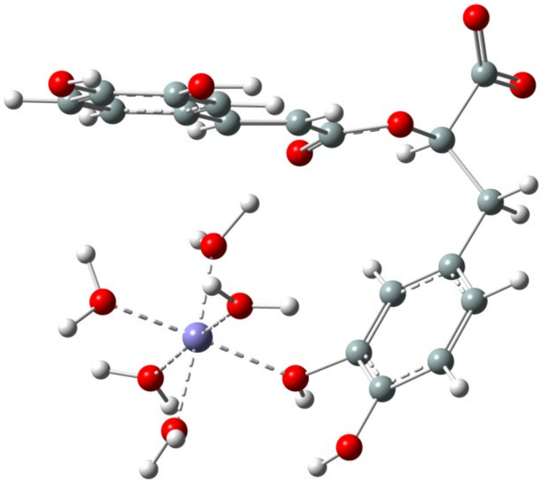

H	-2.89548300	-2.12334300	-1.39036300	
H	-3.15912300	-0.00402600	-0.63622300	
H	-2.88172500	-1.81505000	3.22896100	
H	-2.27643300	0.36455500	4.20541100	
H	1.00176800	-2.52705300	0.57172700	
H	1.50712200	-0.59980200	-1.75773500	
H	3.03528400	-2.26257800	1.31313000	
H	3.63993200	0.20536900	-2.14445300	
H	6.02495700	0.46794300	-1.52136000	
H	-3.20958600	2.71710100	-0.19396700	
H	-2.18099500	3.30872500	2.29303400	
H	4.96653600	-2.61765800	2.60218000	
H	7.31879200	-1.10233500	1.35962900	
Fe	-0.55171100	2.71699700	-0.72825300	
O	-0.24731300	4.27702300	0.54279500	
O	1.28017200	3.08795600	-1.46962300	
H	1.88366700	3.79330400	-1.20548100	
H	1.68325100	2.55544000	-2.16649100	
O	0.29728800	1.55347300	0.67843300	
H	-0.14912500	1.00604300	1.33988600	
H	1.25460300	1.45226300	0.76718100	
O	-1.32985900	4.05604500	-2.01546600	
H	-2.23679600	4.36773700	-2.12627700	
H	-0.77685400	4.47597100	-2.68746200	
O	-0.72786400	1.25710500	-1.98867000	
H	-0.73328500	0.18573700	-1.83232500	
H	-1.12262500	1.43637000	-2.85066200	
H	-0.45789200	5.19478900	0.33134000	
H	0.32118200	4.25520000	1.32271100	

Zero-point correction= 0.439428 (Hartree/Particle)
Thermal correction to Energy= 0.476532
Thermal correction to Enthalpy= 0.477476
Thermal correction to Gibbs Free Energy= 0.369388
Sum of electronic and zero-point Energies= -2942.294521
Sum of electronic and thermal Energies= -2942.257417
Sum of electronic and thermal Enthalpies= -2942.256473
Sum of electronic and thermal Free Energies= -2942.364561

RmAc-anion-FeIII-5H2O-O4

2 6

O	1.88601500	2.33889900	-0.38497000	
O	1.90771200	5.89137500	-0.08457900	
O	-3.48044100	2.04947600	1.91474400	
O	-3.86055200	-0.04356800	0.49636800	
O	3.29687600	4.49854200	-1.16594100	
O	3.13927100	2.62467200	1.45269300	
O	5.45534900	-3.89330800	-1.76628200	
O	7.29155500	-4.57224300	0.02621100	
C	0.07965900	3.70591200	-1.07737400	
C	1.30095500	3.62807900	-0.17140300	
C	-0.97207900	2.69983600	-0.70164800	
C	-1.73009600	2.90079300	0.44948700	

C	-1.18314900	1.56137200	-1.46715300
C	2.28014800	4.77395300	-0.50373900
C	-2.67878800	1.97253000	0.82106000
C	-2.13812700	0.62050100	-1.10425600
C	-2.86837200	0.84471600	0.03441600
C	2.84496300	1.97660100	0.47269900
C	5.15005100	-1.07086800	0.58141600
C	3.45796500	0.70113700	0.07585300
C	4.42730200	0.17031800	0.82328300
C	4.90584100	-1.88097200	-0.53415200
C	6.12275500	-1.46352300	1.49610200
C	5.61919100	-3.04155900	-0.71449700
C	6.84175900	-2.63496300	1.31575600
C	6.59435300	-3.42707300	0.21288100
H	-0.31776600	4.71361500	-0.99276100
H	0.39596900	3.54900800	-2.10592700
H	1.01107000	3.71694300	0.87063800
H	-1.58397500	3.78390000	1.05458200
H	-0.59829200	1.40130100	-2.35829400
H	-2.30070300	-0.25953500	-1.70017700
H	3.08669500	0.24810200	-0.82837500
H	4.71784500	0.71585600	1.71122400
H	4.16063100	-1.60708600	-1.26511000
H	6.31872500	-0.84474800	2.35763100
H	7.59626300	-2.94329800	2.02119400
H	-3.30490200	2.83368100	2.44230800
H	-4.20097000	0.32934600	1.33334200
H	4.78197800	-3.57239600	-2.37156200
H	6.99633200	-4.99146300	-0.78982500
Fe	-4.83811800	-1.72964200	-0.00400700
O	-5.81687100	-1.46084700	1.74331400
O	-5.93519100	-3.39216500	-0.26170300
H	-6.59352000	-3.72311800	0.36198500
H	-5.90243600	-3.97363100	-1.03185900
O	-6.21538700	-0.64882900	-0.97499700
H	-6.18670100	0.30702100	-1.10972900
H	-7.02743600	-1.00366600	-1.35891600
O	-3.44144200	-2.80330100	0.94105100
H	-2.62862300	-2.45349000	1.32752800
H	-3.47016800	-3.76136900	1.05976900
O	-3.93950700	-2.11104200	-1.74877200
H	-4.28951300	-1.84523300	-2.60943800
H	-3.22929900	-2.75494000	-1.87228900
H	-5.58211700	-1.89350600	2.57470000
H	-6.68310100	-1.04263300	1.83406700

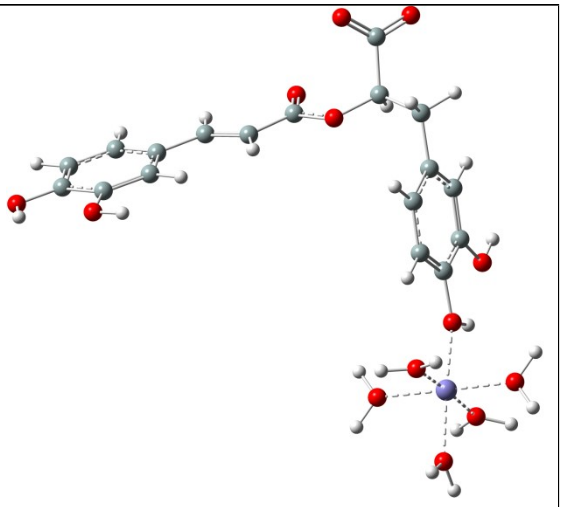

Zero-point correction= 0.440199 (Hartree/Particle)
Thermal correction to Energy= 0.478431
Thermal correction to Enthalpy= 0.479375
Thermal correction to Gibbs Free Energy= 0.366372
Sum of electronic and zero-point Energies= -2942.283305
Sum of electronic and thermal Energies= -2942.245074

Sum of electronic and thermal Enthalpies=	-2942.244130
Sum of electronic and thermal Free Energies=	-2942.357133

RmAc-anion-FeIII-5H2O-O5

2 6

O	1.07976700	-0.39675500	-0.01395500
O	2.63066700	2.61680800	-1.00026900
O	5.71388100	-3.70643300	-1.49995000
O	4.80595400	-5.40359400	0.34637200
O	0.96649300	2.23650800	0.43125400
O	-0.00788900	0.58588900	-1.69478200
O	-5.05075600	-4.03414800	1.66969200
O	-7.11157600	-3.55368000	0.07463400
C	3.32684300	0.04263100	0.64324400
C	2.23075800	0.36900500	-0.36674900
C	3.73016100	-1.40532700	0.58119900
C	4.55334400	-1.85298100	-0.44964800
C	3.27696500	-2.31377900	1.52407700
C	1.93184700	1.85294300	-0.33850700
C	4.90933900	-3.18358400	-0.52917100
C	3.63639400	-3.65363400	1.44766000
C	4.45024000	-4.09281800	0.42345000
C	-0.01217000	-0.19954100	-0.75282500
C	-3.53836500	-1.60638600	-0.64959500
C	-1.14151300	-0.98961000	-0.30066300
C	-2.30885700	-0.90136900	-0.95239300
C	-3.64938400	-2.50248200	0.42229300
C	-4.64742300	-1.38255700	-1.46214900
C	-4.83782200	-3.14716000	0.65892300
C	-5.84671700	-2.03217400	-1.22364600
C	-5.94726500	-2.91474500	-0.16604000
H	4.17451500	0.68623900	0.41943800
H	2.96410200	0.29164700	1.63749400
H	2.54158400	0.11806300	-1.37616600
H	4.92638800	-1.16087300	-1.19202700
H	2.63918100	-1.97785600	2.32672400
H	3.29068300	-4.36642500	2.17931400
H	-0.97662700	-1.62119300	0.55530000
H	-2.35024000	-0.23810300	-1.80609800
H	-2.80953600	-2.69824800	1.07073300
H	-4.56787100	-0.69453800	-2.28892400
H	-6.70917400	-1.86553000	-1.84807200
H	5.98217000	-3.02928500	-2.12619400
H	5.36844700	-5.52907800	-0.42524600
H	-4.25717200	-4.16225800	2.19597700
H	-7.00579100	-4.12920500	0.84085900
Fe	-0.16652800	3.76918900	0.28310100
O	-1.69888100	3.00758100	1.40982500
O	-1.30746800	5.45278700	0.16682400
H	-2.21248200	5.48755300	0.49742100
H	-1.08955800	6.29370300	-0.25000300
O	0.54513900	4.46453100	2.06873900

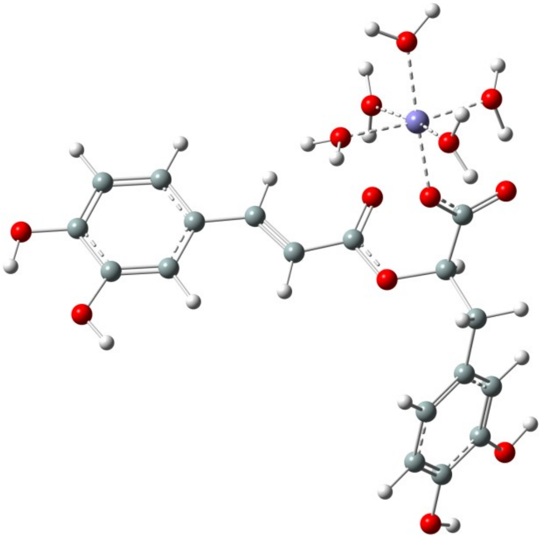

H	1.35477500	4.09657900	2.44149100
H	0.47027500	5.38769400	2.33604000
O	-1.14634500	2.91117100	-1.25517200
H	-0.81129600	2.03606800	-1.57789500
H	-1.61861200	3.37701500	-1.95270000
O	1.23374500	4.67614100	-0.81409200
H	1.94618700	3.94606900	-0.98070000
H	1.06093600	5.15836600	-1.62936800
H	-2.26592900	2.31267600	1.05568900
H	-1.63532800	2.90898100	2.36654500

Zero-point correction= 0.439417 (Hartree/Particle)
Thermal correction to Energy= 0.476841
Thermal correction to Enthalpy= 0.477785
Thermal correction to Gibbs Free Energy= 0.366781
Sum of electronic and zero-point Energies= -2942.366478
Sum of electronic and thermal Energies= -2942.329054
Sum of electronic and thermal Enthalpies= -2942.328110
Sum of electronic and thermal Free Energies= -2942.439114

RmAc-anion-FeIII-5H2O-O6

2 6

O	-1.18181700	0.19864800	0.83110500
O	-2.63216000	-2.99525100	0.98760100
O	-5.72229700	2.52379000	-2.16168000
O	-4.81519000	4.84747700	-1.22268300
O	-1.18985100	-2.00410900	2.36817300
O	0.04807000	-1.35802400	-0.18230000
O	4.51400400	4.72114700	0.91637800
O	6.70657800	3.78571700	-0.22576900
C	-3.47037500	0.06959600	1.47903300
C	-2.33857600	-0.66124400	0.77235900
C	-3.84186000	1.34853000	0.77913600
C	-4.63032700	1.30646900	-0.36864300
C	-3.38771000	2.57382800	1.24015400
C	-2.04267900	-1.99517600	1.43828000
C	-4.95285600	2.47051300	-1.03548800
C	-3.71231600	3.74738800	0.57097700
C	-4.49265000	3.70055900	-0.56614300
C	-0.04508400	-0.21458700	0.34221000
C	3.36230200	1.37540800	-0.09975100
C	1.01058000	0.74389500	0.43385100
C	2.22442100	0.49362900	-0.09342900
C	3.32502100	2.66264700	0.45980700
C	4.54173800	0.92452500	-0.69375000
C	4.43835400	3.46017500	0.41389500
C	5.66609300	1.72726200	-0.73651400
C	5.61982300	2.99467300	-0.18603500
H	-4.31810800	-0.61160300	1.50757600
H	-3.16242900	0.26864800	2.50246400
H	-2.57781700	-0.84108800	-0.27118900
H	-5.00128100	0.36205400	-0.74269700
H	-2.77647600	2.61823000	2.12802100

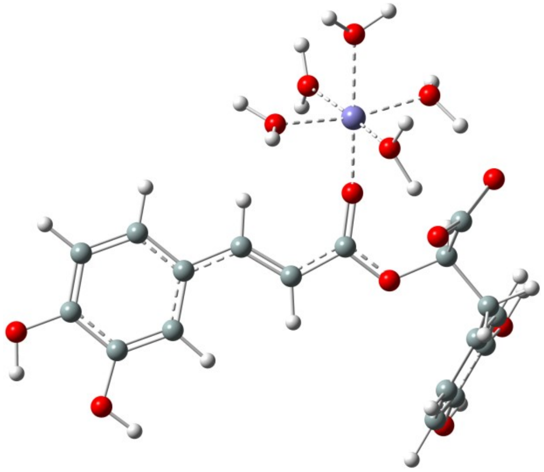

H	-3.36590600	4.70527200	0.92503200
H	0.75331300	1.66701600	0.92300400
H	2.37833600	-0.46119100	-0.57175100
H	2.42873800	3.04127900	0.92555800
H	4.57353000	-0.06324300	-1.12530800
H	6.58208000	1.38988600	-1.19272900
H	-6.01350600	1.64648400	-2.42244800
H	-5.36732600	4.62761200	-1.98057800
H	3.67673600	4.99929100	1.29736800
H	6.50398800	4.63017500	0.19436500
Fe	0.62790100	-3.19610500	-0.41034100
O	-1.20709200	-3.84456200	-0.91020600
O	1.17498900	-5.11354600	-0.87779400
H	0.50818400	-5.79540600	-1.01954800
H	2.01932500	-5.52924700	-0.67167500
O	1.08751200	-2.75809300	-2.34994300
H	0.82758700	-1.94370100	-2.79595300
H	1.31207500	-3.42507200	-3.00898300
O	0.37598300	-3.60197700	1.48112300
H	-0.33529200	-2.96747700	2.03102000
H	0.38644900	-4.50523900	1.81255000
O	2.61150900	-2.88798000	-0.01488600
H	3.33360300	-2.92468500	-0.65253700
H	2.96528600	-2.99093200	0.87641100
H	-1.90107100	-3.57194500	-0.21710700
H	-1.56075700	-3.70083800	-1.79464300

Zero-point correction= 0.438454 (Hartree/Particle)
Thermal correction to Energy= 0.475043
Thermal correction to Enthalpy= 0.475987
Thermal correction to Gibbs Free Energy= 0.367957
Sum of electronic and zero-point Energies= -2942.350378
Sum of electronic and thermal Energies= -2942.313789
Sum of electronic and thermal Enthalpies= -2942.312845
Sum of electronic and thermal Free Energies= -2942.420875

RmAc-anion-FeIII-5H2O-O7

2 6

O	-3.24264200	-1.26620500	0.57620800
O	-6.26033700	-3.12493500	0.96248100
O	-6.03170600	3.03053200	-2.23481200
O	-4.19223200	4.65801400	-1.19352500
O	-4.23834300	-3.39309400	1.89849600
O	-3.03734500	-2.81775200	-1.02949600
O	3.97942100	-0.59537800	0.83151700
O	5.36960100	-2.08857000	-0.91081600
C	-5.26532200	-0.28874100	1.33796800
C	-4.66540700	-1.45307100	0.56199800
C	-4.99028800	1.04042400	0.68866000
C	-5.67344300	1.40092600	-0.47169900
C	-4.04294300	1.91273300	1.20180400
C	-5.07754300	-2.79565200	1.19959200
C	-5.40692300	2.60161500	-1.09766000

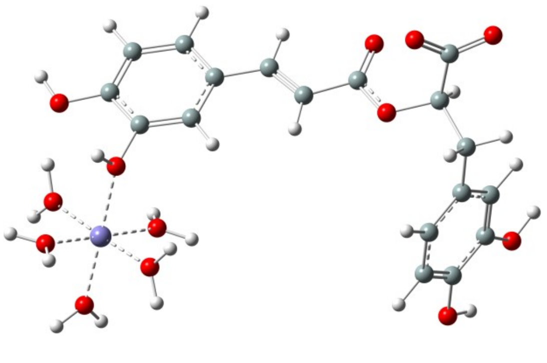

C	-3.77389500	3.12422400	0.57538700	
C	-4.45148900	3.47170800	-0.57493900	
C	-2.53956500	-2.04461000	-0.24299600	
C	1.22493900	-2.38851300	-0.82255600	
C	-1.09073700	-1.81606000	-0.08193800	
C	-0.23161400	-2.49763800	-0.83538300	
C	1.91055900	-1.53066700	0.03696200	
C	1.96339900	-3.16841300	-1.71126100	
C	3.27829600	-1.47519400	-0.02551100	
C	3.34375800	-3.10033100	-1.76744100	
C	4.01450400	-2.23973900	-0.92009600	
H	-6.33550800	-0.46827700	1.39983500	
H	-4.86240100	-0.29973200	2.34870900	
H	-5.00778800	-1.44175900	-0.46783800	
H	-6.42236000	0.74165700	-0.88979000	
H	-3.50668300	1.64768100	2.09982900	
H	-3.03925500	3.80675400	0.97265600	
H	-0.79541500	-1.08502000	0.65254300	
H	-0.64380600	-3.20536400	-1.54143600	
H	1.39746600	-0.91047300	0.75341700	
H	1.44467700	-3.83858400	-2.37799300	
H	3.89952100	-3.70510500	-2.46648100	
H	-6.67419800	2.38374200	-2.53662200	
H	-4.75722200	4.73215300	-1.96976700	
H	4.39855800	-1.06649800	1.56746600	
H	5.79708400	-2.72321200	-1.49673100	
Fe	4.59773400	1.30805400	0.43770900	
O	3.15707500	1.50020100	-0.93273800	
O	5.23307000	3.16813100	0.08877200	
H	4.86680000	3.76994900	-0.57163200	
H	5.99892200	3.57828200	0.51028400	
O	3.33407100	1.98523600	1.83581400	
H	2.72431000	1.44929700	2.35839700	
H	3.24077600	2.91419900	2.08310000	
O	5.80565400	0.52638300	-0.91322600	
H	5.84730300	-0.45134400	-1.04185100	
H	6.37872300	0.99375400	-1.53218800	
O	5.99999700	1.08693700	1.85099300	
H	5.91507500	1.36389000	2.77205700	
H	6.89583800	0.76341400	1.69070000	
H	3.25383100	1.30638300	-1.87395100	
H	2.27666800	1.86079500	-0.76583100	

Zero-point correction= 0.439383 (Hartree/Particle)
Thermal correction to Energy= 0.477734
Thermal correction to Enthalpy= 0.478678
Thermal correction to Gibbs Free Energy= 0.365588
Sum of electronic and zero-point Energies= -2942.289192
Sum of electronic and thermal Energies= -2942.250842
Sum of electronic and thermal Enthalpies= -2942.249898
Sum of electronic and thermal Free Energies= -2942.362988

RmAc-anion-FeIII-5H2O-O8

2 6			
O	-3.90078900	-1.23939700	0.30022600
O	-6.42575200	-3.65881600	-0.37804700
O	-7.12192300	3.12442700	-1.89005100
O	-5.86768400	4.68528200	-0.12595900
O	-4.60080400	-3.76628900	0.92403000
O	-3.09102200	-2.30288200	-1.50063000
O	2.91082300	0.43854200	2.32640500
O	4.81457200	-0.43549300	0.82651200
C	-6.18293000	-0.88245400	0.83727800
C	-5.22497300	-1.66991200	-0.04615700
C	-6.11055200	0.60143200	0.60038800
C	-6.67443700	1.15070900	-0.54968600
C	-5.46806900	1.44357200	1.49482900
C	-5.41628000	-3.18307000	0.18566300
C	-6.59249400	2.50691200	-0.79178000
C	-5.38491000	2.81024100	1.25506600
C	-5.94408300	3.34582300	0.11313700
C	-2.91633400	-1.67145000	-0.48376200
C	0.88352400	-1.30744500	-0.19895700
C	-1.60045300	-1.25952700	0.04396600
C	-0.49005400	-1.62537200	-0.59023500
C	1.17759700	-0.55243600	0.93885600
C	1.92129100	-1.78312400	-0.99604400
C	2.48781200	-0.27449500	1.25507600
C	3.24502900	-1.51851300	-0.67834800
C	3.50188900	-0.75718100	0.43369800
H	-7.18311400	-1.25283200	0.62722700
H	-5.95340800	-1.10349700	1.87773000
H	-5.40056300	-1.45182900	-1.09471800
H	-7.18665200	0.51697100	-1.26100200
H	-5.02706800	1.03256000	2.38975300
H	-4.88877400	3.46955600	1.94970700
H	-1.60841400	-0.67165800	0.94672600
H	-0.60014300	-2.21866600	-1.48734800
H	0.39571400	-0.17832300	1.57971800
H	1.69510500	-2.37424400	-1.86814000
H	4.05000400	-1.91158000	-1.27430900
H	-7.55749600	2.48866000	-2.46325300
H	-6.31480800	4.87715200	-0.95689400
H	2.19315500	0.64205500	2.93363100
H	4.76707000	-0.14375700	1.75768400
Fe	6.29501900	0.53088200	-0.19455200
O	6.31547700	1.87640400	1.29994300
O	7.72515300	1.60748900	-1.08160000
H	8.08100800	2.43921000	-0.74325600
H	8.18619900	1.36819500	-1.89597300
O	7.61299800	-0.61097900	0.78244400
H	7.40548800	-1.39190200	1.31221900
H	8.57101800	-0.48797600	0.75462400
O	4.87959100	1.56475700	-1.15646400

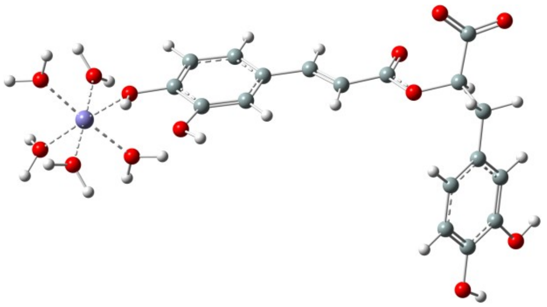

H	3.92127300	1.47270600	-1.06743500
H	5.07036400	2.26505300	-1.79520600
O	6.35072300	-0.76447200	-1.71687200
H	6.91878000	-1.54626000	-1.73624200
H	5.99728300	-0.60315900	-2.60171200
H	5.76226100	2.66801100	1.33370800
H	6.95487500	1.90175300	2.02371800

Zero-point correction= 0.440050 (Hartree/Particle)
Thermal correction to Energy= 0.478521
Thermal correction to Enthalpy= 0.479465
Thermal correction to Gibbs Free Energy= 0.364879
Sum of electronic and zero-point Energies= -2942.278660
Sum of electronic and thermal Energies= -2942.240190
Sum of electronic and thermal Enthalpies= -2942.239245
Sum of electronic and thermal Free Energies= -2942.353831

RmAc-anion-FeIII-4H2O-site1

2 6

O	0.91386900	0.23457900	-1.22859900
O	3.68922600	-0.82096500	-2.68468000
O	2.84018700	4.70945100	2.23183100
O	0.62327500	5.89242100	1.33822800
O	2.68919900	-2.09932000	-1.17523100
O	0.55662500	-1.21605500	0.44618900
O	-6.32808800	1.34791100	-1.40143400
O	-7.70757900	-0.15247100	0.28053600
C	2.82761000	1.59133900	-1.59460900
C	2.35093200	0.27162800	-1.02727300
C	2.25184900	2.75660300	-0.83590900
C	2.85433500	3.18233800	0.34587300
C	1.10136700	3.39607400	-1.27187300
C	2.97960700	-0.94349100	-1.71980000
C	2.31220000	4.22512700	1.06972000
C	0.55414300	4.44580500	-0.54525300
C	1.15465900	4.86237400	0.62565400
C	0.11735600	-0.40587500	-0.41456800
C	-3.60612000	-0.56610800	0.17412100
C	-1.26975300	-0.11067900	-0.57798600
C	-2.17981800	-0.75081800	0.18541200
C	-4.24374900	0.35552400	-0.67209800
C	-4.37609000	-1.33680200	1.04657500
C	-5.60676700	0.48951600	-0.63257700
C	-5.75101300	-1.20297400	1.08544700
C	-6.37075500	-0.29229700	0.24963400
H	3.91274000	1.59154200	-1.53735800
H	2.55305400	1.63543000	-2.64456300
H	2.54824000	0.21446200	0.03901800
H	3.75638900	2.70389200	0.70224900
H	0.62627200	3.07609900	-2.18607000
H	-0.33799700	4.95076900	-0.88003800
H	-1.51517300	0.63377900	-1.31480500
H	-1.80814100	-1.48299000	0.88934900

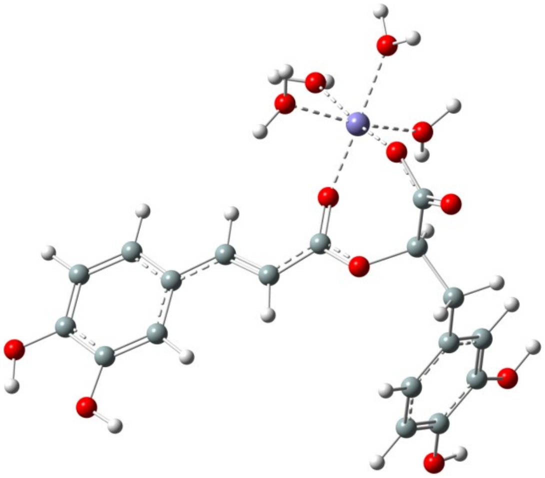

H	-3.67662200	0.96653900	-1.35666300
H	-3.88815500	-2.04434500	1.69780700
H	-6.35574100	-1.79231100	1.75473200
H	3.64322200	4.23818400	2.46749000
H	1.17285500	6.05108400	2.11302800
H	-5.76133400	1.86413900	-1.98087100
H	-7.97475500	0.51999700	-0.35750600
Fe	1.82995000	-2.68996400	0.38692100
O	3.12696800	-4.26223500	0.42934800
H	3.85557300	-4.36909500	-0.19215300
H	3.16690900	-4.94488400	1.10769400
O	3.02688900	-1.71129300	1.73594100
H	2.73558400	-1.05267700	2.37666700
H	3.90676700	-2.02017300	1.97949700
O	0.50053200	-3.93395600	-0.58090700
H	0.83780100	-4.74977600	-0.97000600
H	-0.20965800	-3.60735700	-1.14512600
O	0.98964400	-3.59696300	2.04603300
H	1.12562900	-3.32606800	2.95984600
H	0.14192200	-4.04969000	1.97684900

Zero-point correction= 0.414743 (Hartree/Particle)
Thermal correction to Energy= 0.450612
Thermal correction to Enthalpy= 0.451556
Thermal correction to Gibbs Free Energy= 0.344076
Sum of electronic and zero-point Energies= -2865.912969
Sum of electronic and thermal Energies= -2865.877101
Sum of electronic and thermal Enthalpies= -2865.876157
Sum of electronic and thermal Free Energies= -2865.983636

RmAc-anion-FeIII-4H2O-site2

2 6

O	-0.94370600	0.19764300	1.17366000
O	-2.38090200	-2.30024300	1.21654800
O	-3.31995500	4.44839400	-2.23067100
O	-1.25807900	5.87509800	-1.32395700
O	-3.58057200	-1.14429400	2.68069900
O	-0.52158500	-1.15164700	-0.56578000
O	6.19742200	1.77028500	1.42826500
O	7.69413200	0.36054100	-0.23180400
C	-2.97772600	1.35149300	1.59827800
C	-2.37973100	0.08975900	1.01416900
C	-2.53256300	2.57333600	0.84063900
C	-3.17262100	2.92848600	-0.34473200
C	-1.46398700	3.33815400	1.28267300
C	-2.84536800	-1.18327200	1.73003400
C	-2.74780000	4.02641300	-1.06524400
C	-1.03503200	4.44376800	0.55942100
C	-1.67256400	4.79069300	-0.61447800
C	-0.11849200	-0.35517500	0.32227300
C	3.61936500	-0.27557600	-0.22237500
C	1.24737100	0.02817400	0.49420100
C	2.20539800	-0.54097900	-0.26449400

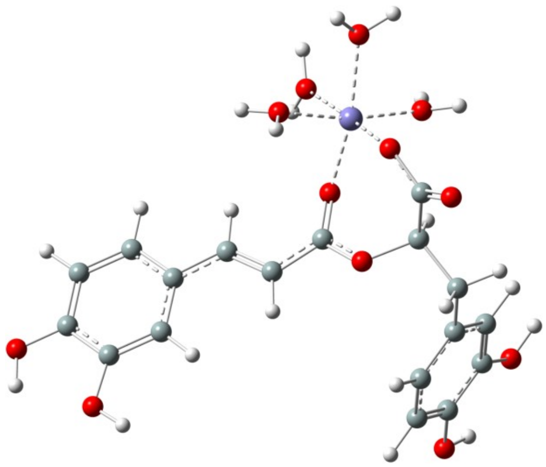

C	4.18679700	0.67136000	0.64528500	
C	4.44935000	-0.99508000	-1.08308200	
C	5.54118400	0.88008000	0.63731700	
C	5.81567000	-0.78676300	-1.08930900	
C	6.36614900	0.14844600	-0.23262200	
H	-4.05817500	1.24291700	1.55733500	
H	-2.69271000	1.41787400	2.64428500	
H	-2.60065300	0.00721700	-0.04717500	
H	-4.01231400	2.35076200	-0.70639600	
H	-0.96037000	3.07310800	2.19920400	
H	-0.20760800	5.04587300	0.89968100	
H	1.43942400	0.76709700	1.25230400	
H	1.89299100	-1.28056100	-0.98810800	
H	3.57212000	1.24423700	1.32170000	
H	4.01548600	-1.72246800	-1.75057600	
H	6.46628300	-1.33641800	-1.74928600	
H	-4.06564500	3.89183700	-2.46845600	
H	-1.81845100	5.97109200	-2.10137900	
H	5.59006300	2.25716000	1.99172400	
H	7.90987100	1.04156700	0.41647500	
Fe	-1.54247900	-2.82659600	-0.37064100	
O	-2.30811800	-4.73308900	-0.48989300	
O	-0.56141500	-3.35193500	-2.09584800	
H	-0.71163700	-4.17196800	-2.57841800	
H	-0.02833500	-2.75354400	-2.63015500	
O	-3.10568100	-2.22230500	-1.54426600	
H	-4.02315100	-2.16185600	-1.25539200	
H	-3.08255000	-2.23252700	-2.50781100	
O	-0.04706000	-3.75195800	0.68047200	
H	0.04283700	-3.62863900	1.63255000	
H	0.81812200	-3.95102500	0.30479500	
H	-1.99025900	-5.42150800	0.10526700	
H	-3.21913300	-4.93056500	-0.73263100	

Zero-point correction= 0.415200 (Hartree/Particle)
Thermal correction to Energy= 0.450807
Thermal correction to Enthalpy= 0.451751
Thermal correction to Gibbs Free Energy= 0.345128
Sum of electronic and zero-point Energies= -2865.914069
Sum of electronic and thermal Energies= -2865.878461
Sum of electronic and thermal Enthalpies= -2865.877517
Sum of electronic and thermal Free Energies= -2865.984140

RmAc-anion-FeIII-4H2O-site3

2 6

O	-1.51145000	2.07184400	0.28874100	
O	-0.60069700	5.40486300	-0.59423100	
O	4.19986600	0.34979400	-0.53651900	
O	3.56541400	-1.56254100	0.95098100	
O	-2.46318300	4.58848100	0.35716800	
O	-2.33663500	2.18715700	-1.79297400	
O	-6.73227900	-2.73317600	2.03807400	
O	-8.36260500	-3.41479300	0.05725100	

C	0.44413600	3.14294900	1.08322400
C	-0.60230700	3.13094600	-0.02390500
C	1.26425200	1.88445600	1.09454100
C	2.36156300	1.77220200	0.24342000
C	0.92450900	0.81540200	1.91602500
C	-1.30885500	4.50236300	-0.09770900
C	3.07160500	0.59942700	0.24940600
C	1.65182100	-0.36691700	1.91418700
C	2.72759500	-0.45217800	1.06676000
C	-2.37889300	1.73343700	-0.67107700
C	-5.33745100	-0.68618600	-0.68828500
C	-3.34940800	0.74448300	-0.18259200
C	-4.29256800	0.27830900	-1.00306900
C	-5.49310700	-1.23961200	0.58836700
C	-6.21625100	-1.06862600	-1.69748200
C	-6.49899400	-2.14376100	0.83118800
C	-7.22964200	-1.98272100	-1.45399200
C	-7.37533600	-2.52302900	-0.19232100
H	1.08126900	4.00598000	0.91601300
H	-0.06022800	3.26742600	2.03860000
H	-0.13921800	2.93169400	-0.98517800
H	2.65138600	2.58744200	-0.40176600
H	0.07544400	0.90620700	2.57348800
H	1.38754900	-1.19211800	2.55535900
H	-3.25332600	0.44188100	0.84691900
H	-4.29114000	0.65374900	-2.01774300
H	-4.83310500	-0.96419700	1.39663500
H	-6.10512400	-0.64581700	-2.68356500
H	-7.91303200	-2.28215400	-2.23199000
H	4.44009000	1.07586200	-1.13059800
H	3.25366500	-2.33049400	1.45005600
H	-6.11359300	-2.41945300	2.70269000
H	-8.32034000	-3.68070700	0.98262300
Fe	5.18414500	-1.41285300	-0.29172800
O	6.60438000	-0.89592600	-1.58436500
H	7.40108600	-0.39524300	-1.36661500
H	6.53936100	-1.01346600	-2.54095000
O	6.32481100	-0.59432700	1.14291300
H	6.88354900	-1.10985000	1.73836000
H	6.32451300	0.32843900	1.42680900
O	4.22698900	-2.22884700	-1.85438300
H	4.26648300	-3.16891300	-2.07214900
H	3.49648300	-1.82166100	-2.33746500
O	6.04289100	-3.16838600	0.09273400
H	6.88207100	-3.43069200	-0.30988300
H	5.72234600	-3.88855400	0.65204800

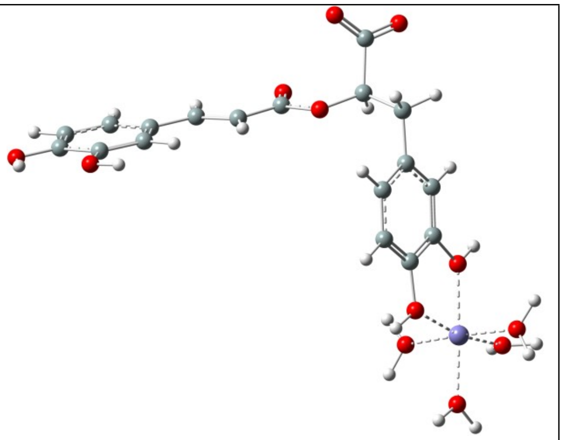

Zero-point correction= 0.414305 (Hartree/Particle)
Thermal correction to Energy= 0.450152
Thermal correction to Enthalpy= 0.451096
Thermal correction to Gibbs Free Energy= 0.343555
Sum of electronic and zero-point Energies= -2865.838040

Sum of electronic and thermal Energies= -2865.802194
Sum of electronic and thermal Enthalpies= -2865.801249
Sum of electronic and thermal Free Energies= -2865.908791

RmAc-anion-FeIII-4H2O-site4

2 6

O	-3.36656700	-1.19156300	0.41818000
O	-5.99024700	-3.57838500	0.74270800
O	-6.98910000	2.85045100	-1.79516500
O	-5.36758500	4.64306600	-0.66455300
O	-3.92087800	-3.53880100	1.61130700
O	-2.96679100	-2.58294000	-1.29489900
O	3.72348300	0.57528000	0.58034000
O	5.26419200	-0.79706200	-0.83583200
C	-5.47937300	-0.64569800	1.34700900
C	-4.73686700	-1.61774200	0.44058200
C	-5.45591200	0.76774300	0.83201900
C	-6.25986500	1.12826800	-0.24776600
C	-4.62730500	1.72638200	1.39463600
C	-4.87844400	-3.05752300	0.97743600
C	-6.22971400	2.41503200	-0.74569600
C	-4.59487800	3.02359700	0.89634900
C	-5.39301400	3.37204500	-0.17374300
C	-2.57561200	-1.79927600	-0.46119600
C	1.20709700	-1.60399800	-1.00365800
C	-1.17086100	-1.37528500	-0.29012800
C	-0.22385100	-1.90285800	-1.05973900
C	1.72284600	-0.59777000	-0.18134400
C	2.07342700	-2.35193200	-1.79802800
C	3.07457100	-0.39265600	-0.18208200
C	3.44366100	-2.13512500	-1.78849700
C	3.92195100	-1.14847100	-0.96602500
H	-6.50229300	-1.00495400	1.42500600
H	-5.03142800	-0.68689200	2.33786200
H	-5.13151100	-1.58077300	-0.56970000
H	-6.91957900	0.40131600	-0.70185900
H	-3.99961000	1.46245400	2.23170400
H	-3.95397000	3.77316900	1.33308700
H	-0.97976600	-0.65214100	0.48539500
H	-0.52511900	-2.63517100	-1.79548600
H	1.08472500	0.01175800	0.43697000
H	1.67054500	-3.12196000	-2.43526000
H	4.10944400	-2.71840100	-2.40322800
H	-7.54618400	2.14390000	-2.13120200
H	-5.99069600	4.70469300	-1.39609300
H	3.13430200	1.05275500	1.18101100
H	5.87519000	-1.34921800	-1.34564100
Fe	5.75395000	0.77477700	0.36391600
O	5.54466900	2.00200500	-1.20684200
O	7.68010700	0.63621300	-0.09559600
H	8.10507700	1.06147800	-0.85185400
H	8.32033300	0.08841500	0.37719100

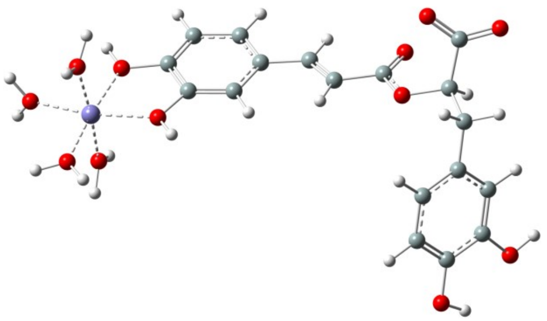

O	6.03252000	2.35032000	1.54434800	
H	5.38711100	2.83758600	2.07376300	
H	6.90352000	2.75409000	1.66173400	
O	6.14179900	-0.45143600	1.90087700	
H	6.26877200	-0.14808900	2.80907900	
H	6.01920900	-1.40930800	1.90076800	
H	5.41557900	1.74168600	-2.12750700	
H	5.49169800	2.96383000	-1.13731200	

Zero-point correction= 0.414376 (Hartree/Particle)
Thermal correction to Energy= 0.450046
Thermal correction to Enthalpy= 0.450990
Thermal correction to Gibbs Free Energy= 0.344425
Sum of electronic and zero-point Energies= -2865.832714
Sum of electronic and thermal Energies= -2865.797045
Sum of electronic and thermal Enthalpies= -2865.796101
Sum of electronic and thermal Free Energies= -2865.902665

Table S9: Cartesian coordinates and thermochemistry properties of optimized structures of 7 monodentate complexes types and 4 bidentate ones between the neutral rosmarinic (RA) and $[Fe(II).6H_2O]^{2+}$ ion in water calculated at the M05-2X/6–311++G(2df,2p) level of theory.

**RmAc-FeII-5H2O-O2**				
2 5				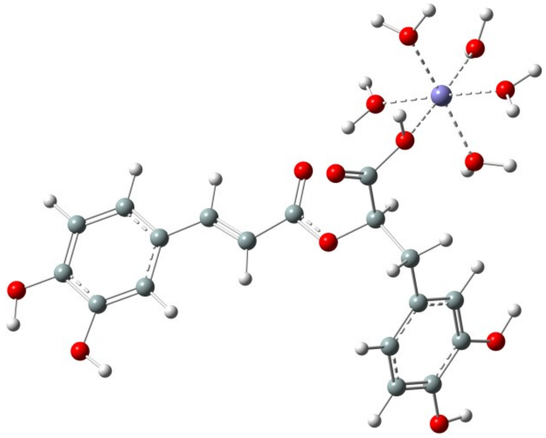
O	0.26994100	0.39268900	1.00737200	
O	-2.73926300	-1.43446300	1.51904200	
O	-2.44803600	4.79074800	-1.88315900	
O	-0.31945000	6.19894100	-1.11201700	
O	-0.86258600	-1.41910800	2.72990600	
O	0.33827900	-1.49288400	-0.19391000	
O	7.58294600	0.70376100	0.66911400	
O	8.72894500	-1.28460500	-0.65314900	
C	-1.69969600	1.51936700	1.73826300	
C	-1.14338000	0.27607200	1.04270400	
C	-1.34141100	2.77723000	0.99504600	
C	-2.10059800	3.18139100	-0.10013400	
C	-0.24236300	3.53427800	1.36977200	
C	-1.51204100	-0.94634400	1.85155400	
C	-1.75938500	4.31903000	-0.80319600	
C	0.10145800	4.68041100	0.66521900	
C	-0.65222000	5.07548400	-0.42169000	
C	0.92598000	-0.57075400	0.34471000	
C	4.60751400	-1.22090600	-0.33294900	
C	2.36421100	-0.36785100	0.36621900	
C	3.15996100	-1.25099200	-0.24919100	
C	5.37679000	-0.20474700	0.24967000	
C	5.25153700	-2.24751000	-1.01932500	
C	6.74515100	-0.23092100	0.14094400	
C	6.63175000	-2.27380200	-1.12999700	
C	7.38243700	-1.26916700	-0.55203000	
H	-2.77950600	1.40495600	1.80321100	
H	-1.29942600	1.54924500	2.74849900	
H	-1.53891700	0.19353800	0.03541500	
H	-2.96860300	2.61281300	-0.40196600	
H	0.35239600	3.23059000	2.21687000	
H	0.95282600	5.27677500	0.95219800	
H	2.72081100	0.50193500	0.89081300	
H	2.68035400	-2.08649100	-0.74100000	
H	4.91008300	0.60654900	0.78683500	
H	4.66452800	-3.03225000	-1.46975200	
H	7.13762800	-3.06472500	-1.65924900	
H	-2.94603100	-2.15818600	2.13338600	
H	-3.19760400	4.22627400	-2.08767100	
H	-0.95134100	6.32403500	-1.82801800	
H	7.09520800	1.38564000	1.13827000	
H	9.08982200	-0.51668900	-0.19530600	
Fe	-3.81534800	-1.98410200	-0.42610900	
O	-3.73965700	-3.87633900	0.51140800	

O	-4.88981300	-2.64808400	-2.11762200
H	-5.51110600	-3.38126100	-2.10894600
H	-4.62027500	-2.50711900	-3.02930900
O	-5.60850600	-1.60992600	0.61646800
H	-5.70303400	-1.10079000	1.42651200
H	-6.48790200	-1.76332000	0.25976500
O	-1.96925900	-2.32763700	-1.29893300
H	-1.10600400	-2.05358600	-0.92910800
H	-1.81611200	-2.86197500	-2.08177900
O	-3.86928800	0.03678800	-1.02734500
H	-4.72308700	0.47858400	-0.96394400
H	-3.48757600	0.27629500	-1.87899200
H	-3.08851800	-4.53244800	0.24379400
H	-4.54778500	-4.34239800	0.74735900

Zero-point correction= 0.450088 (Hartree/Particle)
Thermal correction to Energy= 0.490658
Thermal correction to Enthalpy= 0.491602
Thermal correction to Gibbs Free Energy= 0.372046
Sum of electronic and zero-point Energies= -2942.987139
Sum of electronic and thermal Energies= -2942.946569
Sum of electronic and thermal Enthalpies= -2942.945625
Sum of electronic and thermal Free Energies= -2943.065181

RmAc-FeII-5H2O-O3

2 5

O	-1.71355900	-2.65482300	-0.26073400
O	-4.96794600	-3.68799700	-1.23471100
O	-2.11613400	2.64830400	0.30460400
O	-1.24941500	2.59840800	2.89694600
O	-3.03869700	-4.81515900	-1.11849200
O	-1.17577400	-1.03863900	-1.71590300
O	5.30733800	-2.90557000	1.82846700
O	6.93353400	-1.32318900	0.46391900
C	-3.79479100	-1.98100900	0.74386100
C	-3.09967000	-2.47385000	-0.52756100
C	-3.12447800	-0.75423300	1.30435900
C	-2.97941500	0.39608700	0.53738400
C	-2.60991000	-0.75721900	2.59483200
C	-3.66260600	-3.80012700	-0.98817800
C	-2.34621500	1.50021400	1.06596100
C	-1.98820200	0.36035000	3.12817600
C	-1.85776000	1.51124200	2.36760900
C	-0.83841700	-1.84602900	-0.86627000
C	2.92388900	-1.39336300	-0.53546300
C	0.51605500	-2.05256600	-0.37319900
C	1.52621400	-1.36753600	-0.92244300
C	3.40479800	-2.19421800	0.50967900
C	3.81517400	-0.57698400	-1.22834500
C	4.73779900	-2.16881600	0.83597200
C	5.15958900	-0.54845800	-0.89682600
C	5.62548900	-1.34179300	0.13274800
H	-4.83351800	-1.77874200	0.49687800

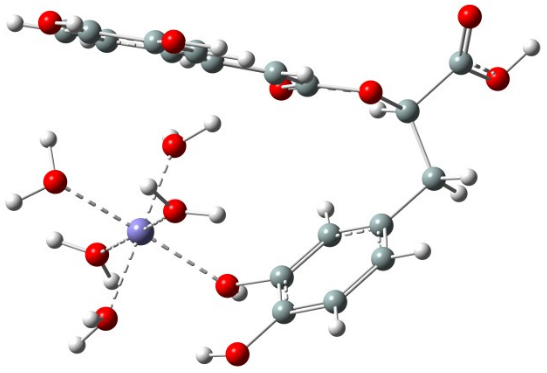

H	-3.77697600	-2.77251900	1.48853400
H	-3.22561300	-1.76181300	-1.33664000
H	-3.34336000	0.44235900	-0.47838900
H	-2.69882900	-1.64694400	3.19841900
H	-1.60134600	0.35713800	4.13429900
H	0.63442500	-2.75320700	0.43552500
H	1.28901900	-0.70359700	-1.74337900
H	2.74127700	-2.83777800	1.06637200
H	3.45115200	0.03695700	-2.03724900
H	5.85568600	0.07979800	-1.42803100
H	-5.30740800	-4.54519800	-1.52604300
H	-2.91938700	2.92983600	-0.14735500
H	-1.20531900	3.31281400	2.24988100
H	4.66029500	-3.46604200	2.26482000
H	7.08550200	-1.93587700	1.19276500
Fe	-0.16534300	2.88753300	-0.66426200
O	0.27302600	4.26066500	0.89116900
O	1.85249100	3.09795700	-1.43919400
H	2.35022900	3.91835800	-1.37767100
H	2.02261300	2.73027200	-2.31179500
O	0.70971200	1.41558500	0.57810500
H	0.31022700	0.75649900	1.15407700
H	1.64499600	1.20898200	0.48425000
O	-0.86176000	4.47808400	-1.86700000
H	-1.71932000	4.91083700	-1.88555800
H	-0.29154100	4.93432500	-2.49191000
O	-0.54501800	1.51859800	-2.15854100
H	-0.73540900	0.56613300	-1.99958700
H	-1.04023700	1.79700900	-2.93409000
H	0.18341000	5.20600100	0.72955100
H	1.06073800	4.13061100	1.43027000

Zero-point correction= 0.450829 (Hartree/Particle)
Thermal correction to Energy= 0.489674
Thermal correction to Enthalpy= 0.490618
Thermal correction to Gibbs Free Energy= 0.378512
Sum of electronic and zero-point Energies= -2942.993792
Sum of electronic and thermal Energies= -2942.954947
Sum of electronic and thermal Enthalpies= -2942.954003
Sum of electronic and thermal Free Energies= -2943.066109

RmAc-FeII-5H2O-O4

2 5

O	1.76478500	2.44914000	-0.40911500
O	1.96521800	5.93834400	0.19755500
O	-3.64347900	2.25771500	1.81254400
O	-4.06310800	0.23418100	0.19772200
O	3.23321800	4.64676800	-1.11998700
O	3.13155100	2.70236500	1.34609900
O	4.71661500	-4.12307400	-1.67543800
O	6.61812200	-4.86015600	0.02263100
C	0.02334100	3.94591100	-1.02840600
C	1.23498900	3.73300900	-0.12439800

C	-1.07037100	2.95812200	-0.72626200
C	-1.84203200	3.11352500	0.42280100
C	-1.31013000	1.88226500	-1.56616100
C	2.27831400	4.79369400	-0.40877100
C	-2.83071600	2.19881100	0.72227400
C	-2.31051800	0.96260000	-1.27478300
C	-3.05612300	1.12487900	-0.13120100
C	2.74437000	2.02929600	0.41963800
C	4.80321500	-1.17584400	0.53222400
C	3.22744200	0.70205900	0.04019900
C	4.19868800	0.12759400	0.75517500
C	4.41634600	-2.01547700	-0.51962900
C	5.80518600	-1.60141800	1.39984400
C	5.02138200	-3.23737800	-0.68559800
C	6.41557800	-2.83441700	1.23381300
C	6.02806100	-3.65545700	0.19373400
H	-0.33110300	4.96226700	-0.87502600
H	0.34245900	3.84957200	-2.06302400
H	0.95250800	3.79737100	0.92222400
H	-1.67726100	3.95201200	1.08507500
H	-0.71920100	1.75876800	-2.45960700
H	-2.51679800	0.13239400	-1.92956000
H	2.76436600	0.24478000	-0.81789700
H	4.58593200	0.68592600	1.59699000
H	3.64374900	-1.71699100	-1.21111300
H	6.10942200	-0.96019000	2.21210000
H	7.19216900	-3.16986900	1.90180200
H	2.61482100	6.61198100	-0.04562400
H	-3.47325000	3.04493600	2.33636900
H	-4.53367800	0.56702900	0.97617200
H	4.02915000	-3.77898500	-2.25178500
H	6.23270200	-5.29447600	-0.74666000
Fe	-4.37202400	-1.90895400	0.03092900
O	-5.18918700	-1.61804800	1.97294000
O	-4.86780900	-3.97203200	0.02941200
H	-5.45056400	-4.36753100	0.68347300
H	-4.30279100	-4.66866700	-0.31602800
O	-6.26524100	-1.43932400	-0.76793600
H	-6.42985900	-0.66345200	-1.31136800
H	-6.90910000	-2.11126600	-1.00936400
O	-2.48693500	-2.36868100	0.85208200
H	-1.77226800	-1.74369300	1.00366800
H	-2.32495300	-3.14011600	1.40255000
O	-3.58487300	-2.12102800	-1.91918800
H	-4.12981400	-2.44559700	-2.64262900
H	-2.70404600	-2.49198300	-2.03210900
H	-4.69486500	-1.76062000	2.78522100
H	-6.11824700	-1.78080200	2.16046500

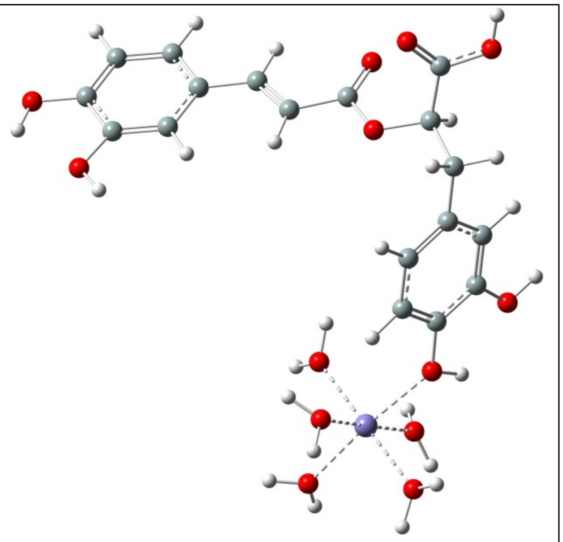

Zero-point correction= 0.448641 (Hartree/Particle)
Thermal correction to Energy= 0.488408
Thermal correction to Enthalpy= 0.489352

Thermal correction to Gibbs Free Energy= 0.372749
Sum of electronic and zero-point Energies= -2942.987582
Sum of electronic and thermal Energies= -2942.947815
Sum of electronic and thermal Enthalpies= -2942.946871
Sum of electronic and thermal Free Energies= -2943.063474

RmAc-FeII-5H2O-O5

2 5

O	1.13371300	-0.36453200	0.08022400
O	2.57147000	2.70090800	-0.90902400
O	5.95143800	-3.32463400	-1.53975200
O	5.17446400	-5.12450200	0.26526700
O	0.86593500	2.29796400	0.47552300
O	0.03149900	0.49962100	-1.65760200
O	-4.76643800	-4.39811800	1.68427300
O	-6.81975100	-4.08739400	0.03838000
C	3.35536700	0.20433900	0.73097300
C	2.22954600	0.46870300	-0.26684700
C	3.85401200	-1.21130000	0.62715500
C	4.69188900	-1.57596500	-0.42441700
C	3.47001400	-2.17192500	1.54849900
C	1.80663200	1.91528300	-0.20308700
C	5.12889600	-2.87892600	-0.54616800
C	3.91243200	-3.48358900	1.43010800
C	4.73910200	-3.84161100	0.38495200
C	0.04975300	-0.26068500	-0.70330600
C	-3.37117500	-1.91381700	-0.64859800
C	-1.03016300	-1.12634900	-0.26228400
C	-2.18639600	-1.12959500	-0.93799700
C	-3.44457100	-2.79709900	0.43690500
C	-4.47499900	-1.77953900	-1.48724300
C	-4.59185800	-3.51675900	0.66113600
C	-5.63267500	-2.50515400	-1.26145100
C	-5.69615800	-3.37463700	-0.19034900
H	4.15583100	0.90946000	0.51927000
H	2.98185500	0.40312500	1.73216900
H	2.55380400	0.25771100	-1.28160000
H	5.01139600	-0.84064900	-1.15010000
H	2.82081100	-1.90000900	2.36603100
H	3.62057500	-4.23702000	2.14422500
H	-0.83923000	-1.73279900	0.60641000
H	-2.25540000	-0.48147000	-1.80149000
H	-2.60808100	-2.92422700	1.10649300
H	-4.42446000	-1.10129800	-2.32435600
H	-6.49086000	-2.40799300	-1.90616400
H	2.24907100	3.62158000	-0.86202800
H	6.16453400	-2.61831800	-2.15476800
H	5.73371100	-5.19213700	-0.51598900
H	-3.97608200	-4.46599400	2.22641300
H	-6.69095300	-4.64175000	0.81657800
Fe	-0.58432000	3.83183500	0.29178200
O	-1.98791600	2.75844300	1.45495200

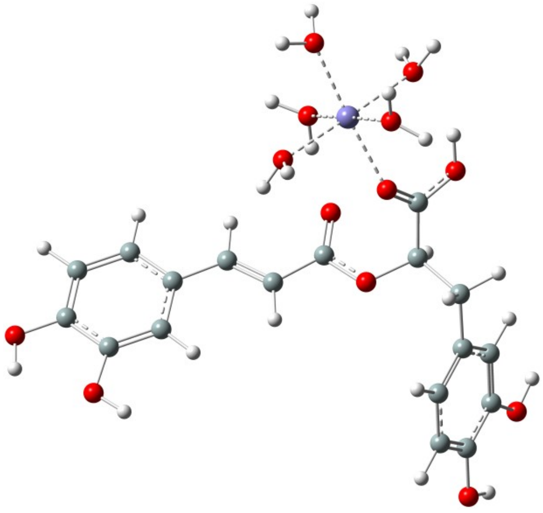

O	-1.93577000	5.45862800	0.15517200
H	-2.75828400	5.42842700	0.65264000
H	-2.10957800	5.94131400	-0.65781200
O	0.20676300	4.69732800	2.04550600
H	1.11284700	4.57616800	2.34274700
H	-0.11084200	5.53409600	2.39621800
O	-1.27038000	2.91316700	-1.47563000
H	-0.90817800	2.03137800	-1.68265200
H	-2.19300100	2.92766200	-1.74366300
O	0.81926200	4.93413100	-0.96642000
H	1.07672400	5.82888100	-0.71648000
H	0.56680700	4.96223500	-1.89725000
H	-2.21302900	1.84549600	1.25156700
H	-1.92985600	2.83210000	2.41243600

Zero-point correction= 0.450504 (Hartree/Particle)
Thermal correction to Energy= 0.489033
Thermal correction to Enthalpy= 0.489977
Thermal correction to Gibbs Free Energy= 0.376603
Sum of electronic and zero-point Energies= -2943.000595
Sum of electronic and thermal Energies= -2942.962066
Sum of electronic and thermal Enthalpies= -2942.961122
Sum of electronic and thermal Free Energies= -2943.074497

RmAc-FeII-5H2O-O6

2 5

O	1.05688200	0.40436400	-1.26054200
O	3.58312300	-1.92219400	-2.20227200
O	4.83128700	3.16512200	2.39146600
O	3.36472400	5.31034800	1.79634200
O	1.39337500	-1.87783400	-2.63460300
O	0.31342600	-1.13201000	0.17053700
O	-5.58917800	3.45068200	-1.36177900
O	-7.36284200	2.31510100	0.24264900
C	3.33580900	0.88906700	-1.72378300
C	2.35761700	-0.17348400	-1.22822700
C	3.35659800	2.08027700	-0.80485700
C	4.11389900	2.04057900	0.36371200
C	2.60757200	3.21244800	-1.08391800
C	2.36830400	-1.41356200	-2.09755100
C	4.11585400	3.11502900	1.22989800
C	2.60880500	4.29542100	-0.21405000
C	3.35923800	4.25066300	0.94334700
C	0.09512500	-0.14664400	-0.53142000
C	-3.54135000	0.75229900	0.08223500
C	-1.16124000	0.55976700	-0.65192600
C	-2.21932600	0.16217600	0.06881000
C	-3.88289700	1.86297500	-0.70189600
C	-4.50692700	0.18547700	0.91166500
C	-5.15354500	2.37838700	-0.64521100
C	-5.78868200	0.70516700	0.96939100
C	-6.11675300	1.80031300	0.19409300
H	4.31900500	0.43248400	-1.77986300

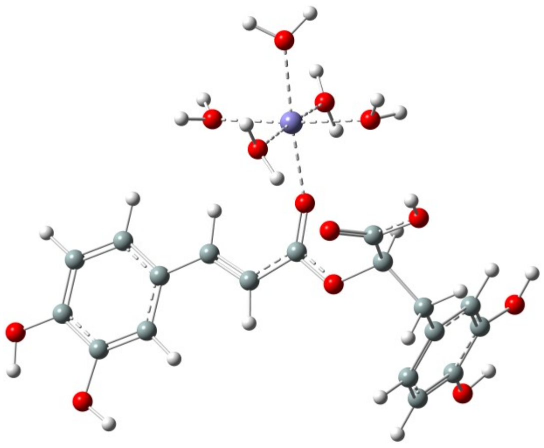

H	3.04312900	1.18840300	-2.72711200	
H	2.60454600	-0.47764400	-0.21369200	
H	4.71235400	1.17074700	0.59780800	
H	2.01708000	3.25385400	-1.98577600	
H	2.03134300	5.18121100	-0.42567200	
H	-1.17189400	1.39894100	-1.32577000	
H	-2.08520200	-0.69218900	0.71641000	
H	-3.15906300	2.32495300	-1.35528700	
H	-4.24922900	-0.66976500	1.51655800	
H	-6.54175600	0.27377700	1.60832100	
H	3.55423000	-2.71902400	-2.75073300	
H	5.34203700	2.36156500	2.51709000	
H	3.94516400	5.11020000	2.53834800	
H	-4.89297300	3.80621400	-1.92040200	
H	-7.41854400	3.06931200	-0.35554000	
Fe	0.12945400	-3.15258200	0.67753700	
O	2.20149000	-3.40274500	0.24530600	
O	0.11928600	-5.24410200	1.12934100	
H	0.72952900	-5.64272700	1.75626700	
H	-0.71561700	-5.71434000	1.20664800	
O	0.64374100	-2.60131600	2.66053500	
H	0.80710400	-1.67735900	2.86985600	
H	0.37236400	-3.04287200	3.46963400	
O	-0.41100500	-3.53524100	-1.32697700	
H	0.11030900	-3.04957800	-1.98530600	
H	-0.55665800	-4.42587000	-1.65638600	
O	-1.93606500	-3.04541900	1.17321000	
H	-2.27191100	-3.11248000	2.07169700	
H	-2.60079700	-3.41265200	0.58312800	
H	2.52471200	-4.19452400	-0.19573500	
H	2.83036400	-3.18967700	0.94196300	

Zero-point correction= 0.449173 (Hartree/Particle)
Thermal correction to Energy= 0.488372
Thermal correction to Enthalpy= 0.489316
Thermal correction to Gibbs Free Energy= 0.374189
Sum of electronic and zero-point Energies= -2942.999692
Sum of electronic and thermal Energies= -2942.960493
Sum of electronic and thermal Enthalpies= -2942.959549
Sum of electronic and thermal Free Energies= -2943.074676

RmAc-FeII-5H2O-O7

2 5

O	-3.20431000	-1.24619100	0.55265700	
O	-6.24195400	-3.07497600	0.67839700	
O	-5.99532300	3.20803500	-2.14353900	
O	-4.08065000	4.73206400	-1.08631400	
O	-4.33783000	-3.38976300	1.81272000	
O	-3.00444100	-2.85952800	-0.98690700	
O	3.99467600	-0.67109300	0.90704400	
O	5.40896000	-2.21529100	-0.75107300	
C	-5.24816000	-0.26753300	1.27861900	
C	-4.61288300	-1.40063300	0.47588200	

C	-4.94671000	1.07509900	0.67074900	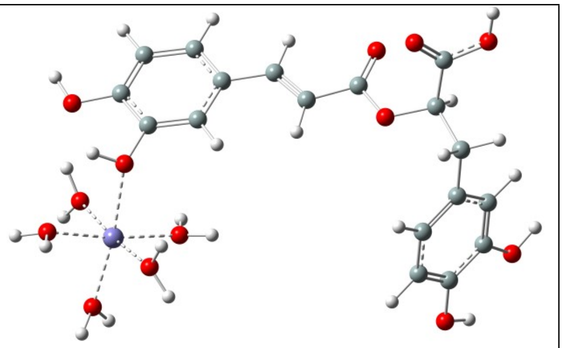
C	-5.64769000	1.49878000	-0.45634200	
C	-3.95848000	1.89307200	1.19471300	
C	-5.01166900	-2.73780900	1.06391800	
C	-5.35773100	2.71411200	-1.04195700	
C	-3.66682200	3.11864400	0.60864700	
C	-4.36190400	3.53202400	-0.50942600	
C	-2.48620200	-2.05889500	-0.24571500	
C	1.27040400	-2.44924200	-0.77281600	
C	-1.04777500	-1.82432000	-0.08467500	
C	-0.18396200	-2.54152100	-0.80369100	
C	1.95217400	-1.58450700	0.08696200	
C	2.00887000	-3.26720300	-1.62487200	
C	3.32241300	-1.54653700	0.06707700	
C	3.39270900	-3.22475600	-1.64293400	
C	4.05429700	-2.35424600	-0.79926000	
H	-6.32105400	-0.44189000	1.30440500	
H	-4.87214100	-0.31527800	2.29766000	
H	-4.93612600	-1.36663800	-0.56024300	
H	-6.42880000	0.88119200	-0.87848400	
H	-3.40912200	1.57531000	2.06707300	
H	-2.90140500	3.76200900	1.01262600	
H	-0.75653800	-1.06654000	0.62315600	
H	-0.59481700	-3.27299300	-1.48632300	
H	1.43237700	-0.94694300	0.78363400	
H	1.49261500	-3.94498500	-2.28589300	
H	3.95719400	-3.85662200	-2.31069500	
H	-6.49217700	-3.90291700	1.11060000	
H	-6.66482400	2.59494100	-2.45715200	
H	-4.66254200	4.85679400	-1.84358800	
H	4.78759600	-1.08954100	1.26965100	
H	5.84588500	-2.89468400	-1.27498700	
Fe	4.56585200	1.39906400	0.39275500	
O	2.87269400	1.66886000	-0.82418300	
O	5.36542700	3.30428300	-0.02553100	
H	5.03942100	3.82725300	-0.76343200	
H	5.66886300	3.91519000	0.65186200	
O	3.49099200	2.04594900	2.08039900	
H	3.05085500	1.42200900	2.66475900	
H	3.04964700	2.89536800	2.16848200	
O	5.52880400	0.55767700	-1.28081100	
H	5.79580400	-0.37285700	-1.28699700	
H	6.08096200	1.04497700	-1.89746400	
O	6.21875800	0.89764100	1.62818000	
H	6.21089700	1.15267400	2.55599800	
H	7.12638800	0.96847100	1.31712500	
H	2.87817400	1.38298300	-1.74220700	
H	1.95901400	1.70853900	-0.52876400	

Zero-point correction= 0.449279 (Hartree/Particle)
Thermal correction to Energy= 0.489513
Thermal correction to Enthalpy= 0.490457

Thermal correction to Gibbs Free Energy= 0.371245
Sum of electronic and zero-point Energies= -2942.989052
Sum of electronic and thermal Energies= -2942.948818
Sum of electronic and thermal Enthalpies= -2942.947874
Sum of electronic and thermal Free Energies= -2943.067086

RmAc-FeII-5H2O-O8

2 5

O	-3.87110700	-1.25138800	0.35428900
O	-6.45195200	-3.43190000	-0.72872000
O	-6.78032100	3.36645100	-1.87407700
O	-5.48780200	4.78025900	-0.01867800
O	-4.82125600	-3.78323600	0.76409000
O	-3.03426700	-2.36728700	-1.39710400
O	2.93457900	0.29933200	2.54061500
O	4.86427200	-0.61384100	1.00933600
C	-6.17326300	-0.78061900	0.73045800
C	-5.17297900	-1.56474500	-0.11591600
C	-6.00612700	0.70350700	0.55014600
C	-6.50044200	1.32344600	-0.59539000
C	-5.34029400	1.46861600	1.49434200
C	-5.42569200	-3.05040200	0.03109300
C	-6.32591800	2.67889600	-0.78555400
C	-5.16586500	2.83422000	1.30659200
C	-5.65499400	3.44272400	0.16878300
C	-2.85451800	-1.72143300	-0.39259100
C	0.93157500	-1.42164700	-0.02406800
C	-1.55790000	-1.33453600	0.17454100
C	-0.43680900	-1.71759800	-0.43582300
C	1.22380200	-0.67553800	1.12202500
C	1.97451100	-1.90351600	-0.80895600
C	2.53212800	-0.41633600	1.45941800
C	3.29606900	-1.65375400	-0.46669500
C	3.56158500	-0.90695100	0.65755600
H	-7.17185600	-1.09110800	0.43277400
H	-6.02996300	-1.05313300	1.77322600
H	-5.26408000	-1.29882700	-1.16481500
H	-7.03006600	0.74912500	-1.34314800
H	-4.95258000	0.99940400	2.38505200
H	-4.65137500	3.43645600	2.03843900
H	-1.58132600	-0.74339400	1.07445200
H	-0.53934000	-2.30944400	-1.33514500
H	0.43665200	-0.29061700	1.75081700
H	1.75361900	-2.48471600	-1.68956800
H	4.11308700	-2.04030500	-1.05357700
H	-6.62711100	-4.37003900	-0.57368100
H	-7.22510600	2.77929600	-2.49047600
H	-5.89367700	5.02995600	-0.85559800
H	2.19300600	0.54027500	3.10294100
H	4.87201100	-0.28253500	1.91977500
Fe	6.18079300	0.55186400	-0.29010700
O	5.92461000	2.19817800	1.00396000

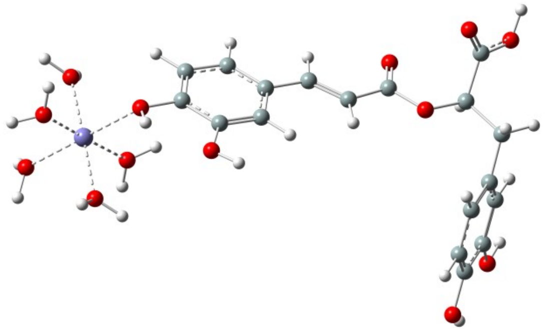

O	7.55408200	1.71516700	-1.42380100	
H	7.76424800	2.60274700	-1.11884000	
H	7.54867100	1.73641200	-2.38496700	
O	7.74559000	-0.13934100	0.93895700	
H	7.78556400	-1.00456900	1.35622200	
H	8.64564800	0.18676300	0.84988400	
O	4.53186600	1.22413500	-1.41441300	
H	3.68936000	0.77131800	-1.51930100	
H	4.48040400	2.06502600	-1.87768700	
O	6.46452400	-1.05971700	-1.62048100	
H	7.30661800	-1.52388500	-1.65794000	
H	6.11891800	-1.01870300	-2.51772900	
H	5.07817300	2.62665600	1.16192400	
H	6.46815500	2.31889000	1.78817900	

Zero-point correction=	0.448665 (Hartree/Particle)
Thermal correction to Energy=	0.489383
Thermal correction to Enthalpy=	0.490327
Thermal correction to Gibbs Free Energy=	0.369439
Sum of electronic and zero-point Energies=	-2942.986400
Sum of electronic and thermal Energies=	-2942.945682
Sum of electronic and thermal Enthalpies=	-2942.944737
Sum of electronic and thermal Free Energies=	-2943.065626

RmAc-FeII-4H2O-site1

2 5				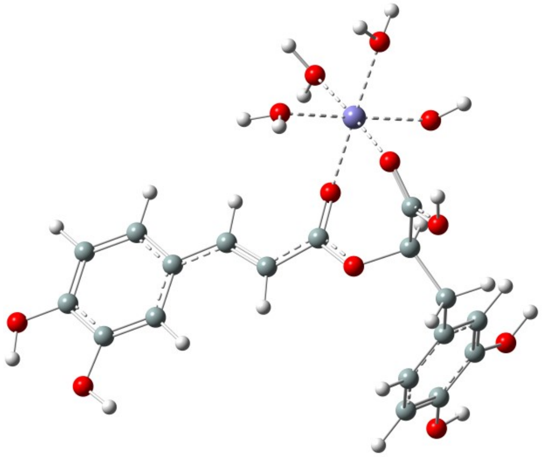
O	-0.87711900	0.24124900	1.26264400	
O	-3.49412300	-0.87668100	2.84856400	
O	-3.15119500	4.46917100	-2.27917300	
O	-1.04204400	5.85940800	-1.42888900	
O	-2.53999600	-2.18676900	1.34154800	
O	-0.53257200	-1.06951800	-0.52102000	
O	6.33867400	1.45832300	1.52815200	
O	7.75683000	0.12849500	-0.26689600	
C	-2.87418500	1.48219800	1.64050600	
C	-2.30208400	0.19327000	1.08187800	
C	-2.39670100	2.66533500	0.84174000	
C	-3.03496800	3.00165400	-0.34997400	
C	-1.30288100	3.41006600	1.25437000	
C	-2.79272300	-1.07209800	1.76735200	
C	-2.58363600	4.06466700	-1.10569800	
C	-0.84700500	4.47989000	0.49492400	
C	-1.48292900	4.80983000	-0.68475500	
C	-0.08493200	-0.34172100	0.36661900	
C	3.65661500	-0.36231700	-0.21853000	
C	1.30730400	-0.01745900	0.55809300	
C	2.22656000	-0.57115400	-0.24898300	
C	4.27543900	0.49432200	0.70352700	
C	4.44289100	-1.04240600	-1.14682200	
C	5.63778900	0.65407000	0.68327600	
C	5.81722000	-0.88198100	-1.16723700	
C	6.41899400	-0.03601600	-0.25561100	
H	-3.95805300	1.40820100	1.60425100	

H	-2.57982300	1.57744300	2.68156200	
H	-2.52626300	0.08893000	0.02363200	
H	-3.89345400	2.43751000	-0.68840300	
H	-0.80097300	3.15790800	2.17537700	
H	0.00033900	5.06714100	0.81109800	
H	1.53739000	0.66838300	1.35476500	
H	1.86902100	-1.24875100	-1.01284200	
H	3.69524100	1.03631800	1.43399500	
H	3.97078900	-1.70176100	-1.85775800	
H	6.43347000	-1.40320000	-1.88130200	
H	-3.77331300	-1.72467100	3.22804100	
H	-3.91783700	3.93213300	-2.49400800	
H	-1.60366000	5.94811000	-2.20631000	
H	5.75832100	1.90818500	2.14780500	
H	8.00729900	0.74924500	0.42729000	
Fe	-1.66910600	-2.82231300	-0.50524700	
O	-2.85194500	-4.56994400	-0.47059200	
H	-2.62323700	-5.28085600	0.13509400	
H	-3.20949100	-4.97330100	-1.26676400	
O	-3.22608400	-1.85484200	-1.58186300	
H	-3.09539700	-1.55795700	-2.48695400	
H	-4.15225200	-2.09581900	-1.48831600	
O	-0.19379300	-3.82975700	0.65277600	
H	0.74266600	-3.81497600	0.43639400	
H	-0.26645700	-3.86779200	1.61079800	
O	-0.71265000	-3.44369400	-2.29093900	
H	-0.19553000	-2.81000000	-2.79646700	
H	-0.29854700	-4.30471300	-2.39709400	

Zero-point correction= 0.424708 (Hartree/Particle)
Thermal correction to Energy= 0.461838
Thermal correction to Enthalpy= 0.462782
Thermal correction to Gibbs Free Energy= 0.351541
Sum of electronic and zero-point Energies= -2866.553810
Sum of electronic and thermal Energies= -2866.516679
Sum of electronic and thermal Enthalpies= -2866.515735
Sum of electronic and thermal Free Energies= -2866.626977

RmAc-FeII-4H2O-site2

2 5

O	-0.90369000	0.22869800	1.34270000	
O	-2.34955400	-2.19146500	1.47842400	
O	-3.19688400	4.35595700	-2.30210400	
O	-1.06696400	5.75284400	-1.51586600	
O	-3.61250600	-0.98125900	2.86722100	
O	-0.56919300	-1.00236600	-0.49548200	
O	6.32143800	1.41635600	1.60983300	
O	7.71532800	0.18722300	-0.27265300	
C	-2.90024700	1.48190900	1.69911500	
C	-2.33063500	0.17764300	1.17799800	
C	-2.42584900	2.64148000	0.86533000	
C	-3.07348400	2.94744200	-0.32964000	
C	-1.32224900	3.38976400	1.24443000	

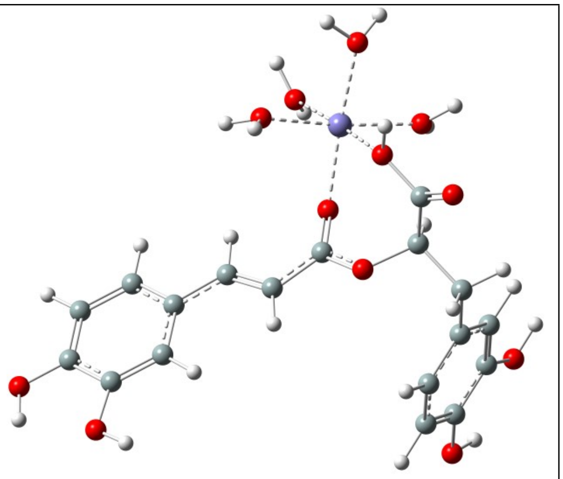

C	-2.85193600	-1.01790900	1.95409000
C	-2.62108600	3.98287300	-1.12215500
C	-0.86543300	4.43217900	0.44816500
C	-1.50994400	4.73095100	-0.73514500
C	-0.11550800	-0.30912500	0.42225900
C	3.61513800	-0.29263800	-0.21077100
C	1.27569100	0.00772800	0.61275800
C	2.18575800	-0.49852000	-0.23644700
C	4.24593300	0.50887100	0.75212100
C	4.38956400	-0.92154400	-1.18442200
C	5.60843400	0.66487500	0.72760600
C	5.76389600	-0.76437000	-1.20960800
C	6.37757000	0.02662200	-0.25723100
H	-3.98362400	1.40196600	1.66942900
H	-2.60351300	1.60221200	2.73720000
H	-2.56715300	0.03600800	0.12663100
H	-3.93983700	2.38034100	-0.64208800
H	-0.81287800	3.16130900	2.16755400
H	-0.01005600	5.02153000	0.73774200
H	1.51408400	0.64925800	1.44311600
H	1.81849200	-1.12942400	-1.03463200
H	3.67454900	1.01026800	1.51768400
H	3.90776300	-1.53823600	-1.92647900
H	6.37139400	-1.24595900	-1.95812900
H	-2.68960100	-2.92318200	2.01759200
H	-3.96676100	3.81559000	-2.49599200
H	-1.63346700	5.82081300	-2.29178600
H	5.74997500	1.83347300	2.25997900
H	7.97506500	0.76723000	0.45275100
Fe	-1.57547300	-2.78141200	-0.57388400
O	-2.70452500	-4.53517000	-0.28590000
O	-0.71747800	-3.34040500	-2.42006800
H	-0.62827800	-4.25469700	-2.70336200
H	-0.75680900	-2.79545400	-3.21081700
O	-3.32220300	-1.91423900	-1.40203200
H	-4.22280600	-2.08163300	-1.10922300
H	-3.36744000	-1.60794900	-2.31231900
O	0.03245800	-3.82851800	0.33153800
H	0.20958400	-3.85561700	1.27620100
H	0.87468700	-3.91814400	-0.12373400
H	-2.23958200	-5.32090700	0.01877100
H	-3.39497700	-4.82271500	-0.89070800

Zero-point correction= 0.424956 (Hartree/Particle)
Thermal correction to Energy= 0.462586
Thermal correction to Enthalpy= 0.463530
Thermal correction to Gibbs Free Energy= 0.350756
Sum of electronic and zero-point Energies= -2866.545045
Sum of electronic and thermal Energies= -2866.507414
Sum of electronic and thermal Enthalpies= -2866.506470
Sum of electronic and thermal Free Energies= -2866.619245

RmAc-FeII-4H2O-site3

2 5

O	-1.53660200	2.15969200	0.33873300
O	-0.68834100	5.41845600	-0.77634400
O	4.00329300	0.34536500	-0.69007100
O	3.51194600	-1.47267300	1.08650300
O	-2.43623300	4.74238400	0.44883000
O	-2.51877900	2.41408700	-1.65693000
O	-6.27844500	-3.18409200	1.91933700
O	-8.05229600	-3.72380700	0.02258700
C	0.43722100	3.27038300	1.05832800
C	-0.64244700	3.19944200	-0.01892800
C	1.24998600	2.00580700	1.10428800
C	2.25630900	1.79668800	0.16559400
C	0.99658600	1.02883600	2.05501800
C	-1.38590700	4.51774900	-0.08515900
C	2.98373200	0.62910300	0.19427400
C	1.73209600	-0.14818400	2.08257900
C	2.72512800	-0.34042400	1.14921400
C	-2.46462800	1.84660200	-0.59109300
C	-5.27429100	-0.72773100	-0.63489700
C	-3.33565800	0.76759800	-0.12732400
C	-4.31832900	0.33019000	-0.91949500
C	-5.27411100	-1.43994100	0.57107400
C	-6.22308200	-1.04335600	-1.60344100
C	-6.19744800	-2.43434300	0.78440300
C	-7.15581800	-2.04533400	-1.38827700
C	-7.14712000	-2.74359900	-0.19744500
H	1.07520600	4.12203600	0.83682900
H	-0.04250400	3.44447200	2.01796900
H	-0.20455400	2.99946100	-0.99246900
H	2.47761300	2.54492600	-0.58155200
H	0.21772400	1.18292600	2.78436500
H	1.53434400	-0.90787300	2.82276900
H	-3.13500900	0.37049600	0.85337600
H	-4.42237300	0.80860700	-1.88423900
H	-4.55513900	-1.21932400	1.34508700
H	-6.22983600	-0.49891600	-2.53453400
H	-7.89292000	-2.29492400	-2.13405900
H	-1.15739700	6.26375000	-0.75658300
H	4.13556100	1.04626200	-1.33699100
H	3.33905000	-2.08003400	1.81342500
H	-5.59712800	-2.93653600	2.54977900
H	-7.90228600	-4.10225900	0.89629600
Fe	5.13586300	-1.47928900	-0.35824300
O	6.39742600	-1.37502000	-2.04456400
H	6.64929300	-0.56339000	-2.49283300
H	6.83108300	-2.10666200	-2.49170100
O	6.31418200	-0.30941200	0.92207500
H	7.05494400	-0.69074200	1.40229600
H	6.50918800	0.61748500	0.75775300
O	3.86108100	-2.62340300	-1.56123700

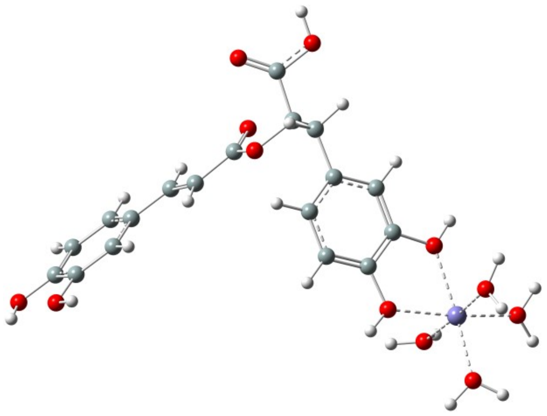

H	2.96971300	-2.84961700	-1.28029200
H	3.85330200	-2.51889900	-2.51727100
O	6.21241600	-3.10790400	0.44673300
H	7.07605500	-3.39116900	0.13444000
H	5.79313400	-3.86483800	0.86496100

Zero-point correction= 0.423624 (Hartree/Particle)
Thermal correction to Energy= 0.462340
Thermal correction to Enthalpy= 0.463284
Thermal correction to Gibbs Free Energy= 0.346454
Sum of electronic and zero-point Energies= -2866.547013
Sum of electronic and thermal Energies= -2866.508298
Sum of electronic and thermal Enthalpies= -2866.507354
Sum of electronic and thermal Free Energies= -2866.624183

RmAc-FeII-4H2O-site4

2 5

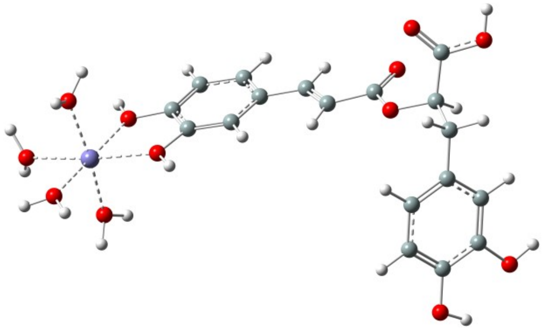

O	3.41928700	-1.17235700	-0.49619300
O	6.16753600	-3.41689600	-0.53822500
O	6.85195400	3.03514800	1.79930800
O	5.15572300	4.73172000	0.63611800
O	4.20262700	-3.54546300	-1.60293300
O	3.03754200	-2.62328200	1.16590800
O	-3.70302400	0.47048600	-0.62169500
O	-5.18918600	-0.74414700	1.08314700
C	5.55843800	-0.55106900	-1.33428900
C	4.79418200	-1.51943900	-0.43430100
C	5.46181600	0.86347700	-0.83145300
C	6.23491800	1.26797800	0.25479300
C	4.59189500	1.77191100	-1.41290400
C	4.98418200	-2.93996900	-0.92306300
C	6.13140000	2.55396500	0.74418400
C	4.48804200	3.06829800	-0.92385900
C	5.25348000	3.46276100	0.15431500
C	2.61858600	-1.81093600	0.37693500
C	-1.13582700	-1.59395100	1.02241600
C	1.22418300	-1.37687700	0.23109900
C	0.28971800	-1.90545300	1.02015800
C	-1.70293400	-0.66893000	0.14025300
C	-1.95311200	-2.24482400	1.94118400
C	-3.04994600	-0.41962800	0.19964500
C	-3.31497900	-1.99068000	1.99683500
C	-3.85474600	-1.07676500	1.12267000
H	6.59568300	-0.87585400	-1.36240900
H	5.15293300	-0.62312400	-2.34051800
H	5.14886300	-1.45427100	0.58998100
H	6.92711100	0.57926400	0.71981200
H	3.98844600	1.46980300	-2.25457700
H	3.81534900	3.78152200	-1.37305600
H	1.02664100	-0.63353900	-0.52281100
H	0.60935400	-2.64382500	1.74268200
H	-1.10182700	-0.14796000	-0.58775600
H	-1.52015900	-2.95942300	2.62228400

H	-3.94592600	-2.49642200	2.71039900	
H	6.28589100	-4.30240800	-0.90805400	
H	7.42607100	2.35608700	2.16226000	
H	5.76041200	4.82958800	1.37927900	
H	-3.11095300	0.91697200	-1.23595000	
H	-5.71625700	-1.23343700	1.72458300	
Fe	-5.85590200	0.70356800	-0.38293400	
O	-5.69465400	2.20041900	1.06810200	
O	-7.97341900	0.70410700	0.08143300	
H	-8.24876700	1.01027300	0.95030900	
H	-8.48465400	-0.08581800	-0.11669900	
O	-6.44687100	2.07160000	-1.86224400	
H	-5.90125900	2.63587000	-2.41573700	
H	-7.34367800	2.41785700	-1.86075900	
O	-6.28310300	-0.83863000	-1.72829700	
H	-6.38171600	-0.62843700	-2.66207200	
H	-5.95156700	-1.73904600	-1.66071000	
H	-5.21960900	2.08619200	1.89678300	
H	-5.62799000	3.12652300	0.81589500	

Zero-point correction= 0.424171 (Hartree/Particle)
Thermal correction to Energy= 0.462341
Thermal correction to Enthalpy= 0.463285
Thermal correction to Gibbs Free Energy= 0.348983
Sum of electronic and zero-point Energies= -2866.544133
Sum of electronic and thermal Energies= -2866.505963
Sum of electronic and thermal Enthalpies= -2866.505019
Sum of electronic and thermal Free Energies= -2866.619321

Table S10: Cartesian coordinates and thermochemistry properties of optimized structures of 7 monodentate complexes types and 4 bidentate ones between the neutral rosmarinic (RA) and $[Fe(III).6H_2O]^{3+}$ ion in water calculated at the M05-2X/6–311++G(2df,2p) level of theory.

RmAc-FeIII-5H2O-O2

3 6			
O	-0.92594500	-1.13324000	0.35534100
O	2.20999000	0.40245800	1.21380800
O	5.87022600	-2.40039600	1.28807000
O	6.56851700	-2.04488800	-1.25517900
O	0.60979600	-0.43119700	2.55539500
O	-1.30973100	0.89236100	-0.54006300
O	-8.16007200	-2.18604600	0.59932000
O	-9.61477800	-0.13488300	-0.22627500
C	1.08535100	-2.36534200	0.06074300
C	0.47214100	-0.96107800	0.20451400
C	2.54862200	-2.32099900	-0.28489600
C	3.51899800	-2.41479000	0.70769900
C	2.94837900	-2.16178500	-1.60797700
C	1.01533700	-0.31146100	1.45738900
C	4.85964900	-2.32153800	0.38631500
C	4.29886400	-2.06409500	-1.93336000
C	5.25639500	-2.13605300	-0.93984000
C	-1.73860100	-0.16618500	-0.08127900
C	-5.49971800	0.19947300	-0.28848800
C	-3.12993200	-0.51768600	0.03482500
C	-4.06614700	0.37253500	-0.33304800
C	-6.10459800	-0.98232600	0.16401700
C	-6.30506400	1.25454100	-0.71442800
C	-7.47193600	-1.08871600	0.18233900
C	-7.68441100	1.14799600	-0.69504100
C	-8.27231100	-0.01994000	-0.24841200
H	0.90980600	-2.91416200	0.98154400
H	0.52359800	-2.84521900	-0.73511100
H	0.69109000	-0.36954500	-0.67782100
H	3.23429000	-2.57181400	1.73869900
H	2.20912200	-2.13654700	-2.39521500
H	4.61645900	-1.94744000	-2.95705200
H	-3.34687400	-1.49716800	0.42342700
H	-3.72102100	1.32759300	-0.70475200
H	-5.51058900	-1.81751500	0.50093800
H	-5.84367500	2.16464200	-1.06351600
H	-8.31548700	1.95780400	-1.02249900
H	2.73533500	0.39111600	2.03432700
H	5.53997600	-2.55682900	2.17713300
H	7.09547900	-2.13567400	-0.45334000
H	-7.56581600	-2.88953700	0.87362900
H	-9.85485800	-1.00722100	0.10752200
Fe	2.54891400	2.11079100	0.02144500
O	0.62684100	2.34340900	-0.18764100

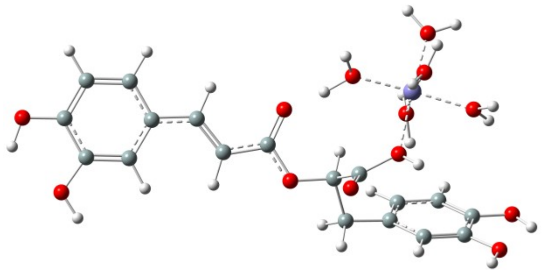

O	2.96509200	3.67978300	-1.13770500
H	2.30933000	4.19327200	-1.62691100
H	3.85242900	3.97935900	-1.37412900
O	2.52960200	3.31882400	1.64238600
H	2.06196700	3.18051500	2.47613200
H	2.81019500	4.24231300	1.59506300
O	2.75539400	0.84306400	-1.49654100
H	3.01659100	-0.10321700	-1.46862500
H	2.73641500	1.13472400	-2.41798300
O	4.49968700	1.86033100	0.44536100
H	4.96067300	2.27321900	1.18672200
H	5.13221100	1.36415400	-0.08966000
H	-0.15496900	1.65924400	-0.38074700
H	0.22350000	3.11142800	0.23800400

Zero-point correction= 0.453599 (Hartree/Particle)
Thermal correction to Energy= 0.490363
Thermal correction to Enthalpy= 0.491307
Thermal correction to Gibbs Free Energy= 0.383916
Sum of electronic and zero-point Energies= -2942.720325
Sum of electronic and thermal Energies= -2942.683561
Sum of electronic and thermal Enthalpies= -2942.682616
Sum of electronic and thermal Free Energies= -2942.790008

RmAc-FeIII-5H2O-O3

3 6

O	-1.43523100	-2.72208100	-0.27023700
O	-4.57326000	-4.03419200	-1.28383800
O	-2.22478500	2.49712100	0.32915800
O	-1.55614700	2.52772500	3.00611700
O	-2.54440800	-4.97423600	-1.17154300
O	-1.03642700	-1.03224700	-1.66800200
O	5.52488100	-2.41285300	1.94192500
O	7.09638300	-0.87404500	0.47146100
C	-3.58410700	-2.24499900	0.71269300
C	-2.83316200	-2.65877900	-0.55351200
C	-3.04689500	-0.96555000	1.29918800
C	-2.94773400	0.19091000	0.53827100
C	-2.61893000	-0.92225500	2.62298800
C	-3.26556800	-4.02819600	-1.03251600
C	-2.43493200	1.33369300	1.11264300
C	-2.13713500	0.24002900	3.19787700
C	-2.04969200	1.40173300	2.44449000
C	-0.62412400	-1.83475100	-0.82744900
C	3.09683300	-1.14695700	-0.51927000
C	0.73239200	-1.93127000	-0.33604900
C	1.70408100	-1.20293900	-0.90759200
C	3.60261000	-1.86106500	0.57711700
C	3.96069000	-0.34461700	-1.26350500
C	4.93182300	-1.76714900	0.90214700
C	5.30198100	-0.25071300	-0.93628000
C	5.79246200	-0.95908100	0.14352100
H	-4.63378700	-2.13814500	0.45267600

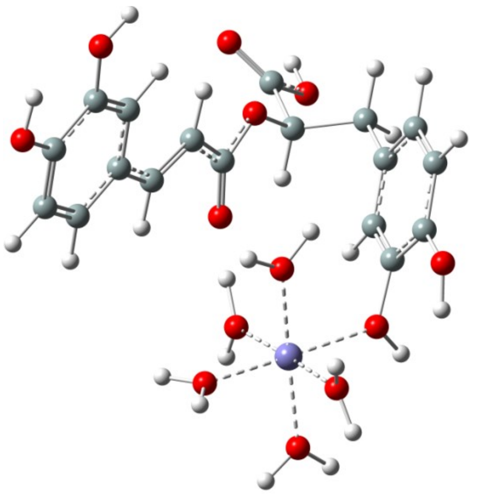

H	-3.50341000	-3.03833200	1.45071000	
H	-3.01154900	-1.95598900	-1.35978900	
H	-3.25142500	0.21826400	-0.49696000	
H	-2.67197300	-1.81692400	3.22300500	
H	-1.82658500	0.26755500	4.22929400	
H	0.89549900	-2.61078800	0.48242200	
H	1.43685900	-0.58333600	-1.75424100	
H	2.96039300	-2.48764200	1.17614500	
H	3.57988700	0.20491000	-2.10974000	
H	5.97765600	0.36449600	-1.50739200	
H	-4.83295300	-4.91440600	-1.58877800	
H	-3.06258900	2.89249600	0.04946000	
H	-1.73050500	3.29857500	2.45614000	
H	4.89474000	-2.95209500	2.42706000	
H	7.26891600	-1.42950700	1.24076700	
Fe	-0.46398600	2.80238300	-0.70697800	
O	0.01986600	4.28815900	0.59020100	
O	1.30337900	3.11113100	-1.60661600	
H	1.95840400	3.78693300	-1.39111200	
H	1.61025700	2.58134800	-2.35353400	
O	0.44721600	1.54103300	0.57267300	
H	0.04951900	1.01949000	1.28448800	
H	1.40582900	1.41359500	0.58134000	
O	-1.28320800	4.21581900	-1.87497900	
H	-2.19333000	4.53115400	-1.94192900	
H	-0.74548200	4.69491500	-2.51970000	
O	-0.79338600	1.38496700	-2.00353000	
H	-0.81123900	0.34581700	-1.84757100	
H	-1.23600900	1.58284800	-2.83785500	
H	-0.20258500	5.21711800	0.44923000	
H	0.69655400	4.22700900	1.27639700	
Zero-point correction= 0.453061 (Hartree/Particle)<br>Thermal correction to Energy= 0.490368<br>Thermal correction to Enthalpy= 0.491312<br>Thermal correction to Gibbs Free Energy= 0.382175<br>Sum of electronic and zero-point Energies= -2942.731627<br>Sum of electronic and thermal Energies= -2942.694320<br>Sum of electronic and thermal Enthalpies= -2942.693376<br>Sum of electronic and thermal Free Energies= -2942.802513				
**RmAc-FeIII-5H2O-O4**				
3 6				
O	1.89497700	2.31742300	-0.42461700	
O	1.95194000	5.83093100	0.06713100	
O	-3.48101300	2.05363800	1.92925600	
O	-3.85250500	-0.03579000	0.50426000	
O	3.26889400	4.54707900	-1.20990800	
O	3.26659100	2.66920100	1.30976600	
O	5.21862000	-4.09156300	-1.64736400	
O	7.15074700	-4.70849500	0.06188000	
C	0.08225000	3.72010100	-1.05241400	
C	1.31803500	3.58565200	-0.16615200	

C	-0.96884900	2.71183300	-0.67723100
C	-1.72852200	2.91224300	0.47198300
C	-1.17302700	1.57654500	-1.44744600
C	2.31189700	4.68007400	-0.49933000
C	-2.67650500	1.98098700	0.83891900
C	-2.12814600	0.63432400	-1.08880200
C	-2.86136700	0.85484100	0.04848900
C	2.90398600	1.96072000	0.40029200
C	5.12992300	-1.12884200	0.53943400
C	3.44935100	0.65383400	0.03652000
C	4.45555200	0.14166800	0.75076600
C	4.79356000	-1.99660200	-0.50714200
C	6.15168900	-1.49114000	1.41270700
C	5.46782600	-3.18320300	-0.66257500
C	6.83037500	-2.68934100	1.25835400
C	6.49308900	-3.53807700	0.22312900
H	-0.30618400	4.72848500	-0.93509800
H	0.38160500	3.58740100	-2.08851800
H	1.05209300	3.67034500	0.88341900
H	-1.58798100	3.79541200	1.07813800
H	-0.58531000	1.41938200	-2.33704500
H	-2.28848100	-0.24420100	-1.68741300
H	3.00382000	0.16139900	-0.81131100
H	4.81809700	0.73109200	1.58234700
H	4.00800500	-1.74684800	-1.20359000
H	6.41737300	-0.82724000	2.22018700
H	7.62240800	-2.97560500	1.93105800
H	2.57165500	6.52277000	-0.20164800
H	-3.31164900	2.83746700	2.45953700
H	-4.19753500	0.33350300	1.34092800
H	4.52481400	-3.78534500	-2.23709800
H	6.79418300	-5.16832400	-0.70655500
Fe	-4.82908200	-1.72586900	-0.00355300
O	-5.81073600	-1.45747400	1.74145900
O	-5.92369000	-3.38696300	-0.26630100
H	-6.58289100	-3.71895900	0.35607700
H	-5.88981300	-3.96741100	-1.03730300
O	-6.20273600	-0.64141900	-0.97358200
H	-6.17217200	0.31440400	-1.10849000
H	-7.01384900	-0.99524800	-1.36063800
O	-3.43245800	-2.79469800	0.94569300
H	-2.62199500	-2.44268700	1.33522400
H	-3.46065700	-3.75251400	1.06725000
O	-3.92194000	-2.10200800	-1.74455700
H	-4.27053500	-1.83836000	-2.60654600
H	-3.21291600	-2.74782600	-1.86555000
H	-5.57869900	-1.89143300	2.57300300
H	-6.67746600	-1.03965800	1.83006300

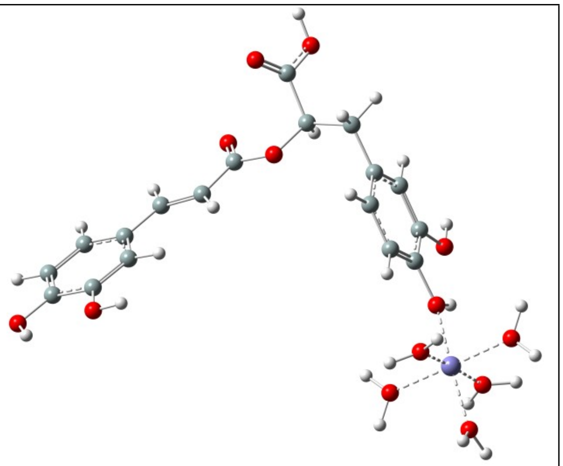

Zero-point correction= 0.453157 (Hartree/Particle)
Thermal correction to Energy= 0.491639
Thermal correction to Enthalpy= 0.492583

Thermal correction to Gibbs Free Energy= 0.379128
Sum of electronic and zero-point Energies= -2942.724790
Sum of electronic and thermal Energies= -2942.686308
Sum of electronic and thermal Enthalpies= -2942.685363
Sum of electronic and thermal Free Energies= -2942.798819

RmAc-FeIII-5H2O-O5

3 6

O	1.04667000	-0.43375900	-0.01463700
O	2.86405000	2.31604600	-1.23931500
O	5.42903600	-4.15008000	-1.43862200
O	4.34752700	-5.71128500	0.42918200
O	1.24836200	2.26928200	0.29875000
O	-0.00432300	0.64811300	-1.65153500
O	-5.24673500	-3.79863500	1.64550400
O	-7.26764200	-3.23824300	0.03164000
C	3.34219100	-0.15705800	0.57163900
C	2.24150800	0.20747000	-0.42942600
C	3.62035400	-1.63506300	0.54923500
C	4.41800500	-2.17544300	-0.45682200
C	3.07358400	-2.47470600	1.50583100
C	2.06040200	1.69893700	-0.44769800
C	4.65815900	-3.53333200	-0.49740400
C	3.31666000	-3.84153600	1.46761700
C	4.10590300	-4.37456700	0.46882200
C	-0.05128800	-0.16794100	-0.73091400
C	-3.62415600	-1.41847600	-0.64786200
C	-1.20633100	-0.90517900	-0.28507700
C	-2.37107500	-0.76016600	-0.93849000
C	-3.77650000	-2.31434300	0.42046900
C	-4.71661000	-1.15105500	-1.47123800
C	-4.98885200	-2.91655200	0.64209800
C	-5.93968800	-1.75816700	-1.24790700
C	-6.08100000	-2.64104200	-0.19454800
H	4.23328400	0.40550100	0.30419700
H	3.02151200	0.15679100	1.56143600
H	2.50967000	-0.10572400	-1.43434700
H	4.86066100	-1.53671400	-1.20877100
H	2.45416900	-2.06436500	2.28778500
H	2.89876600	-4.50307100	2.20947100
H	-1.06526300	-1.55825000	0.55858200
H	-2.38500100	-0.08730300	-1.78535600
H	-2.95068600	-2.54111100	1.07670400
H	-4.60333800	-0.46301000	-2.29395300
H	-6.78971500	-1.55939300	-1.87972200
H	2.76004300	3.28251800	-1.20014900
H	5.77267800	-3.51654800	-2.07352700
H	4.91385700	-5.90390500	-0.32581100
H	-4.46654100	-3.95679300	2.18352900
H	-7.19155100	-3.81966200	0.79727100
Fe	0.01390200	3.79804800	0.34131900
O	-1.20677500	2.92715900	1.68334900

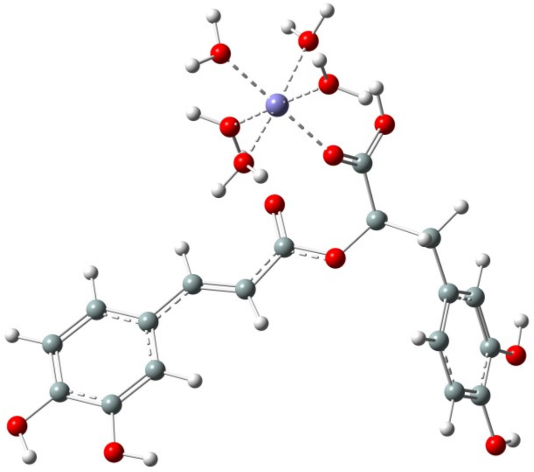

O	-1.23513200	5.37058100	0.41865300
H	-2.04903900	5.36382600	0.93845000
H	-1.20575600	6.17275000	-0.11703900
O	1.08120100	4.59011100	1.84703000
H	1.87749300	4.20362100	2.23247700
H	0.84426000	5.39041800	2.33209500
O	-0.95453400	2.96578400	-1.15870300
H	-0.70634600	2.04800000	-1.48509700
H	-1.72793100	3.31510300	-1.61608000
O	1.19824400	4.83805100	-0.98469300
H	1.69696500	5.61728600	-0.70028100
H	0.90341300	4.98396200	-1.89471200
H	-1.86361600	2.26367400	1.43695800
H	-0.99063400	2.82746500	2.61883600

Zero-point correction= 0.453624 (Hartree/Particle)
Thermal correction to Energy= 0.491252
Thermal correction to Enthalpy= 0.492196
Thermal correction to Gibbs Free Energy= 0.381447
Sum of electronic and zero-point Energies= -2942.745466
Sum of electronic and thermal Energies= -2942.707838
Sum of electronic and thermal Enthalpies= -2942.706894
Sum of electronic and thermal Free Energies= -2942.817643

RmAc-FeIII-5H2O-O6

3 6

O	1.03814500	0.37536300	-1.26428900
O	3.63534900	-1.80564600	-2.32422800
O	4.68644700	3.27613400	2.38650800
O	3.10927100	5.34770100	1.81652100
O	1.42130300	-1.91509300	-2.58198100
O	0.32965500	-1.21329000	0.12379700
O	-5.66725700	3.22243200	-1.43718400
O	-7.38532100	2.11126000	0.23095600
C	3.28922200	0.96340900	-1.74192600
C	2.36871900	-0.14913800	-1.24963000
C	3.25440900	2.14610200	-0.81217300
C	4.01875900	2.13408500	0.35238200
C	2.44675800	3.24012600	-1.07881900
C	2.40697800	-1.38166700	-2.12932300
C	3.96951800	3.19932100	1.22810700
C	2.39699800	4.31397100	-0.19928100
C	3.15413000	4.29733700	0.95451000
C	0.08210500	-0.18871900	-0.57501000
C	-3.54485200	0.62682100	0.08362100
C	-1.17479400	0.46544400	-0.67503400
C	-2.22589900	0.06337600	0.07298600
C	-3.91668700	1.70218100	-0.74128700
C	-4.49052300	0.06971300	0.94826700
C	-5.19425800	2.19182200	-0.68901800
C	-5.77851100	0.56428300	1.00214700
C	-6.13570800	1.62366800	0.18723800
H	4.29191700	0.55251200	-1.80457200

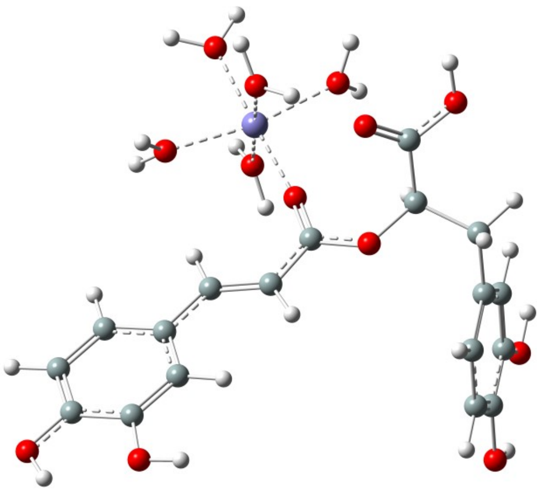

H	2.97844900	1.25359100	-2.74213900
H	2.63724100	-0.44375200	-0.23784600
H	4.66277700	1.29457300	0.57553300
H	1.85095200	3.25969300	-1.97794500
H	1.77442800	5.17105900	-0.40059100
H	-1.20931800	1.29932300	-1.35382000
H	-2.07135800	-0.76405400	0.74746500
H	-3.21022600	2.15161800	-1.42118600
H	-4.20894200	-0.75725700	1.58061500
H	-6.51682200	0.14388000	1.66463400
H	3.62992900	-2.59890300	-2.88060700
H	5.23403800	2.49620700	2.50660700
H	3.70144000	5.17085000	2.55528700
H	-4.99097900	3.57691300	-2.02086500
H	-7.46886000	2.84094900	-0.39495700
Fe	0.25675900	-3.03534700	0.68215000
O	2.26969800	-3.28809400	0.41379600
O	0.27524900	-4.95098200	1.35792300
H	1.05722300	-5.48996600	1.52440000
H	-0.51413900	-5.47020800	1.55130900
O	0.56444700	-2.41505100	2.56730200
H	0.60242200	-1.48782800	2.83164200
H	0.65254000	-2.97920100	3.34529000
O	-0.08058100	-3.63377900	-1.19230700
H	0.33275600	-3.10708300	-1.90883700
H	-0.17057000	-4.55469000	-1.46553600
O	-1.73658900	-3.15206900	1.04035700
H	-2.13404400	-3.16761700	1.92002600
H	-2.39406600	-3.41659400	0.38502700
H	2.66736900	-3.97145500	-0.14071500
H	2.90338400	-3.04745700	1.10195300

Zero-point correction= 0.454261 (Hartree/Particle)
Thermal correction to Energy= 0.492046
Thermal correction to Enthalpy= 0.492990
Thermal correction to Gibbs Free Energy= 0.382374
Sum of electronic and zero-point Energies= -2942.748588
Sum of electronic and thermal Energies= -2942.710803
Sum of electronic and thermal Enthalpies= -2942.709859
Sum of electronic and thermal Free Energies= -2942.820475

RmAc-FeIII-5H2O-O7

3 6

O	-3.22451800	-1.23426100	0.57835800
O	-6.26719200	-3.05194600	0.75605800
O	-5.98938500	3.18553500	-2.19045100
O	-4.06899400	4.71685500	-1.15462600
O	-4.35467100	-3.35291500	1.88026300
O	-3.02986800	-2.87498200	-0.93264200
O	4.00288800	-0.60984800	0.82616600
O	5.37990400	-2.10218000	-0.92598300
C	-5.26534600	-0.23734500	1.29014000
C	-4.63440300	-1.38791300	0.50941500

C	-4.95621600	1.09306100	0.65962800
C	-5.65311300	1.50177600	-0.47550400
C	-3.96458900	1.91440900	1.17181100
C	-5.03289500	-2.71250600	1.12558800
C	-5.35613700	2.70615200	-1.08006800
C	-3.66590500	3.12892300	0.56667700
C	-4.35704700	3.52760700	-0.55914700
C	-2.51185200	-2.05827600	-0.21036200
C	1.23938200	-2.40943500	-0.80399500
C	-1.07071200	-1.81174300	-0.07050100
C	-0.21528100	-2.52463300	-0.80086700
C	1.92928700	-1.54649800	0.04715200
C	1.97127000	-3.19195800	-1.69590800
C	3.29603400	-1.48886600	-0.02608400
C	3.35054600	-3.11942100	-1.76493000
C	4.02622000	-2.25465700	-0.92531000
H	-6.33900900	-0.40651100	1.31663400
H	-4.89181600	-0.26841600	2.31073800
H	-4.95961100	-1.37466000	-0.52657600
H	-6.43678900	0.88159100	-0.88900500
H	-3.41865100	1.60820500	2.05043200
H	-2.89826700	3.77511500	0.96183700
H	-0.77560400	-1.04358500	0.62453000
H	-0.62975400	-3.26419100	-1.47205100
H	1.42081600	-0.92749900	0.76786000
H	1.44839300	-3.86670000	-2.35471300
H	3.90208900	-3.72475700	-2.46674900
H	-6.51709300	-3.87132900	1.20450100
H	-6.66473300	2.57313700	-2.49267300
H	-4.65149900	4.83411300	-1.91263100
H	4.42358700	-1.08121600	1.56115200
H	5.80445800	-2.73603000	-1.51499200
Fe	4.61938600	1.29593500	0.43656800
O	3.17710100	1.48710200	-0.93207700
O	5.25400900	3.15629600	0.09156600
H	4.88464400	3.76180300	-0.56375100
H	6.02026600	3.56529500	0.51356000
O	3.35572000	1.96799900	1.83700700
H	2.75405100	1.42919300	2.36601700
H	3.26422500	2.89592400	2.08892900
O	5.82640600	0.51727800	-0.91760200
H	5.86650400	-0.45899500	-1.05151100
H	6.39946600	0.98673700	-1.53509000
O	6.02371100	1.07034800	1.84684900
H	5.94142600	1.34613800	2.76852100
H	6.91881500	0.74592900	1.68401300
H	3.27608700	1.30255800	-1.87496100
H	2.29760900	1.84954100	-0.76428700

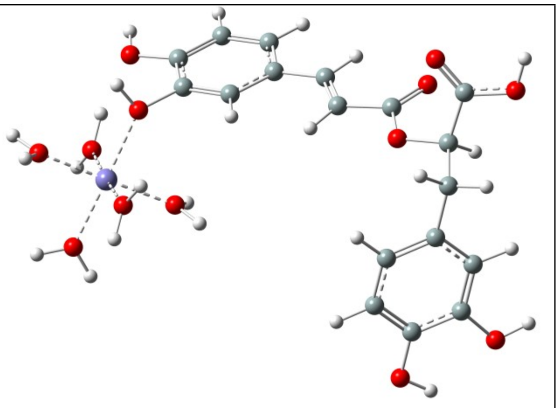

Zero-point correction= 0.452592 (Hartree/Particle)
Thermal correction to Energy= 0.491088
Thermal correction to Enthalpy= 0.492032

Thermal correction to Gibbs Free Energy= 0.379348
Sum of electronic and zero-point Energies= -2942.731099
Sum of electronic and thermal Energies= -2942.692604
Sum of electronic and thermal Enthalpies= -2942.691660
Sum of electronic and thermal Free Energies= -2942.804344

RmAc-FeIII-5H2O-O8

3 6

O	-3.89084600	-1.24977400	0.37042200
O	-6.51795200	-3.44303400	-0.56909100
O	-6.77766800	3.33971800	-1.90719100
O	-5.43631200	4.78174100	-0.10933900
O	-4.84378700	-3.76047500	0.88297600
O	-3.09673700	-2.43110900	-1.35848800
O	2.91566300	0.60755900	2.21079100
O	4.83102700	-0.50639900	0.87234600
C	-6.17868200	-0.74939900	0.79030500
C	-5.20698100	-1.56860400	-0.05629100
C	-5.99629700	0.72742600	0.56906800
C	-6.50187000	1.32513100	-0.58332700
C	-5.30526500	1.50694200	1.48292200
C	-5.46580200	-3.04671700	0.14602200
C	-6.31370000	2.67323100	-0.80972700
C	-5.11691500	2.86511300	1.25857500
C	-5.61720000	3.45158800	0.11408000
C	-2.89625600	-1.74906000	-0.38352700
C	0.89737700	-1.41928300	-0.10052000
C	-1.58428000	-1.33914700	0.13754000
C	-0.47698200	-1.74854500	-0.47700700
C	1.18771500	-0.52226200	0.92906000
C	1.93639000	-2.02583600	-0.80221700
C	2.49897500	-0.23503700	1.23685000
C	3.25951200	-1.75220400	-0.49198200
C	3.51398800	-0.84963800	0.51051200
H	-7.18726500	-1.05454600	0.52238300
H	-6.01622100	-0.99685200	1.83649300
H	-5.32211100	-1.33545000	-1.11068000
H	-7.05098000	0.73962700	-1.30801000
H	-4.90886200	1.05492700	2.37869800
H	-4.58302000	3.47857900	1.96681000
H	-1.58999300	-0.71151500	1.01270100
H	-0.59078900	-2.38802000	-1.34131300
H	0.40307400	-0.03794700	1.48749400
H	1.71066700	-2.72533900	-1.59012600
H	4.06846500	-2.23792400	-1.00990300
H	-6.69535100	-4.37441500	-0.37949700
H	-7.24342000	2.74424300	-2.49965600
H	-5.85361600	5.01621500	-0.94506300
H	2.18513100	0.92864300	2.74804300
H	4.81074300	-0.19198200	1.79500600
Fe	6.21824900	0.51415300	-0.23275100
O	6.32570200	1.83359900	1.28149200

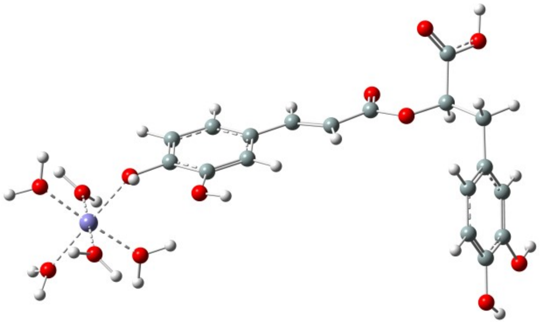

O	7.59777700	1.60000900	-1.17828000
H	8.00389800	2.39944600	-0.81892200
H	7.99639500	1.39191500	-2.03332800
O	7.59068200	-0.63306200	0.65222200
H	7.40135200	-1.42027600	1.17954700
H	8.54659900	-0.53803100	0.54698400
O	4.74070200	1.58062000	-1.05902300
H	3.78961800	1.42746200	-0.97481100
H	4.88982300	2.27079800	-1.71996700
O	6.13489800	-0.76852300	-1.76236400
H	6.71974800	-1.53228800	-1.85794700
H	5.67983600	-0.60856900	-2.60006600
H	5.77720200	2.62539500	1.35692900
H	6.99706500	1.83876600	1.97594500

Zero-point correction= 0.452742 (Hartree/Particle)
Thermal correction to Energy= 0.490625
Thermal correction to Enthalpy= 0.491570
Thermal correction to Gibbs Free Energy= 0.379791
Sum of electronic and zero-point Energies= -2942.721205
Sum of electronic and thermal Energies= -2942.683322
Sum of electronic and thermal Enthalpies= -2942.682377
Sum of electronic and thermal Free Energies= -2942.794156

RmAc-FeIII-4H2O-site1

3 6

O	-0.90865500	0.26813300	1.21443100
O	-3.62188800	-0.76858800	2.66151600
O	-2.85418900	4.74715700	-2.25416000
O	-0.66526200	5.94453700	-1.31712000
O	-2.75261700	-2.08122500	1.12179900
O	-0.54310700	-1.26028000	-0.38549900
O	6.31119700	1.43929000	1.37194500
O	7.70532900	-0.09337300	-0.26092600
C	-2.85576400	1.59363800	1.54212200
C	-2.32718100	0.29249100	0.96953600
C	-2.28238200	2.76996700	0.79792700
C	-2.87666900	3.19897900	-0.38647100
C	-1.14544800	3.41581500	1.25782500
C	-2.92429700	-0.95536600	1.59914300
C	-2.33719600	4.25555400	-1.09154500
C	-0.60223000	4.47966900	0.54991800
C	-1.19292300	4.90141800	-0.62424500
C	-0.09124100	-0.40454100	0.43106900
C	3.61600300	-0.55317100	-0.15143700
C	1.27780800	-0.09351100	0.58134400
C	2.20110300	-0.75645000	-0.16022500
C	4.23983200	0.40358400	0.66986000
C	4.39865600	-1.34238900	-1.00017900
C	5.59908400	0.55269400	0.62978800
C	5.76954700	-1.19262300	-1.04022700
C	6.37464400	-0.24800700	-0.22904200
H	-3.93916100	1.57574600	1.45815600

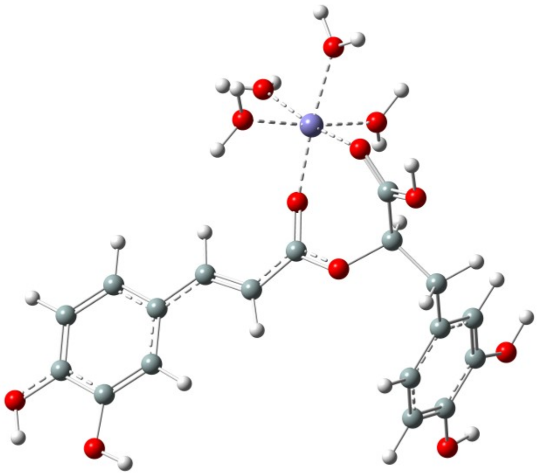

H	-2.60086600	1.64100400	2.59667500	
H	-2.51013900	0.23596400	-0.09976400	
H	-3.76932500	2.71533400	-0.75856200	
H	-0.67929900	3.09201300	2.17512700	
H	0.27882500	4.99194200	0.90201300	
H	1.51540500	0.68355500	1.28592000	
H	1.83965300	-1.51935800	-0.83614000	
H	3.66300100	1.02712000	1.33428200	
H	3.91998200	-2.07492800	-1.63008000	
H	6.38477700	-1.79321800	-1.68933100	
H	-3.97510000	-1.60308100	3.01473900	
H	-3.64661500	4.26836200	-2.50994900	
H	-1.20583100	6.10693000	-2.09760800	
H	5.74103900	1.96906300	1.93597000	
H	7.96566200	0.60068700	0.35725700	
Fe	-1.72979600	-2.75654000	-0.47208500	
O	-3.11983200	-4.22448800	-0.41101600	
H	-3.83370400	-4.27800200	0.23587700	
H	-3.15706400	-4.98596600	-1.00241400	
O	-2.89965000	-1.78856900	-1.81277500	
H	-2.58831300	-1.13194200	-2.44818100	
H	-3.74639500	-2.14038100	-2.11462900	
O	-0.54851100	-3.91250100	0.69073100	
H	-0.85910900	-4.73750100	1.08338100	
H	0.26052000	-3.63715200	1.13797300	
O	-0.86030300	-3.70519900	-2.02441100	
H	-0.99988200	-3.51006000	-2.95836000	
H	-0.07155700	-4.25174700	-1.92522800	

Zero-point correction= 0.427867 (Hartree/Particle)
Thermal correction to Energy= 0.463684
Thermal correction to Enthalpy= 0.464628
Thermal correction to Gibbs Free Energy= 0.358159
Sum of electronic and zero-point Energies= -2866.303670
Sum of electronic and thermal Energies= -2866.267853
Sum of electronic and thermal Enthalpies= -2866.266909
Sum of electronic and thermal Free Energies= -2866.373378

RmAc-FeIII-4H2O-site2

3 6

O	-0.94166400	0.22410000	1.25996700	
O	-2.37766800	-2.31089900	1.29560400	
O	-3.30820300	4.36762100	-2.28207100	
O	-1.24070200	5.80446200	-1.41152600	
O	-3.51972700	-1.13127000	2.81081400	
O	-0.55703700	-1.13326900	-0.47197100	
O	6.20456300	1.71538400	1.50166100	
O	7.66454400	0.37329600	-0.23345000	
C	-2.98923400	1.38090200	1.63465000	
C	-2.36571900	0.11296100	1.08457200	
C	-2.53864300	2.57853500	0.84227300	
C	-3.17594500	2.90050800	-0.35393500	
C	-1.46619000	3.34756800	1.26605400	

C	-2.83608700	-1.10765100	1.85223400
C	-2.74298500	3.97476300	-1.10436500
C	-1.02977800	4.42891900	0.51203100
C	-1.66322700	4.74487800	-0.67282700
C	-0.11171100	-0.32676400	0.40387700
C	3.59586200	-0.24122100	-0.20796200
C	1.23945000	0.04536300	0.55222200
C	2.19270100	-0.50383200	-0.24375800
C	4.17692500	0.66657200	0.69712500
C	4.41421200	-0.92798900	-1.11140600
C	5.52955900	0.86840200	0.68310200
C	5.77836700	-0.72609900	-1.12406800
C	6.34110800	0.16936700	-0.23041600
H	-4.06835400	1.26259300	1.58365000
H	-2.71233200	1.47786100	2.68032000
H	-2.59428200	-0.01080000	0.02983300
H	-4.01840000	2.31745400	-0.70016900
H	-0.96637800	3.10644100	2.19110900
H	-0.19971000	5.03590200	0.83639600
H	1.44576900	0.76661500	1.32306400
H	1.87215700	-1.21745100	-0.98840000
H	3.57209300	1.21013400	1.40535200
H	3.96794400	-1.62376500	-1.80375800
H	6.42093200	-1.24823600	-1.81322300
H	-2.72669600	-3.04246400	1.83650900
H	-4.06367500	3.81751100	-2.50336300
H	-1.79879900	5.88355200	-2.19261600
H	5.61332600	2.17771300	2.10202900
H	7.89411600	1.02249800	0.44313900
Fe	-1.43918400	-2.81292800	-0.52623000
O	-2.36385500	-4.60225200	-0.38288100
O	-0.57339800	-3.34890700	-2.23950100
H	-0.80260800	-4.14894300	-2.72959500
H	-0.02246800	-2.78168400	-2.79374200
O	-3.06175400	-2.10704800	-1.51108100
H	-3.99556600	-2.21265800	-1.28889400
H	-2.99934600	-1.79558300	-2.42375600
O	0.02897900	-3.75106400	0.51625500
H	0.19831000	-3.68484100	1.46452700
H	0.83554000	-4.05281200	0.07845100
H	-1.94772100	-5.37378400	0.02244100
H	-3.20969300	-4.86146400	-0.76829700

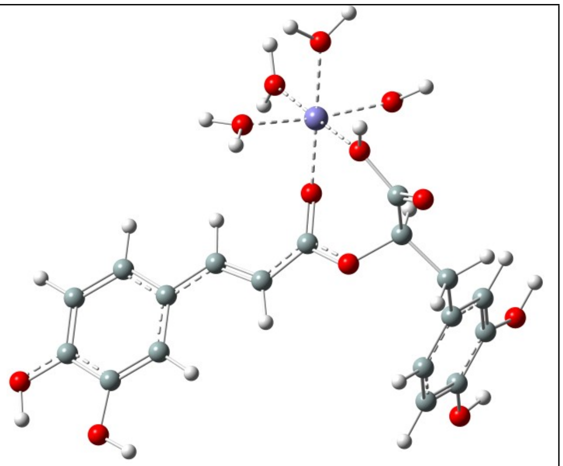

Zero-point correction= 0.428538 (Hartree/Particle)
Thermal correction to Energy= 0.463934
Thermal correction to Enthalpy= 0.464878
Thermal correction to Gibbs Free Energy= 0.359238
Sum of electronic and zero-point Energies= -2866.282217
Sum of electronic and thermal Energies= -2866.246822
Sum of electronic and thermal Enthalpies= -2866.245877
Sum of electronic and thermal Free Energies= -2866.351517

RmAc-FeIII-4H2O-site3

3 6			
O	-1.47148400	2.22301200	0.33906900
O	-0.60969000	5.48127200	-0.77342600
O	4.10362500	0.30177400	-0.53801000
O	3.46536200	-1.47950500	1.09617900
O	-2.37025200	4.80362500	0.43353600
O	-2.45304900	2.45664300	-1.65943500
O	-6.14983600	-3.17273500	1.93602400
O	-7.89340400	-3.76274900	0.02694700
C	0.51019900	3.32753000	1.04131900
C	-0.57783200	3.25827800	-0.02867100
C	1.29462800	2.04638200	1.09833700
C	2.35102100	1.84239100	0.21422000
C	0.95286100	1.05042700	2.00493300
C	-1.31614900	4.58035200	-0.09251400
C	3.02278100	0.64824500	0.27491300
C	1.63887700	-0.15471500	2.05474500
C	2.67594800	-0.33371500	1.17428200
C	-2.39686100	1.89598100	-0.59056100
C	-5.16762800	-0.71926400	-0.62957800
C	-3.25663500	0.81107400	-0.12114200
C	-4.22778200	0.35229200	-0.91571800
C	-5.16529400	-1.42119900	0.58250100
C	-6.10142100	-1.06056300	-1.60404200
C	-6.07172900	-2.43103700	0.79569500
C	-7.01675800	-2.07847100	-1.38922100
C	-7.00572900	-2.76680400	-0.19261500
H	1.16414200	4.16217000	0.80513800
H	0.04057500	3.52060700	2.00204900
H	-0.14810200	3.05469500	-1.00510400
H	2.64052700	2.59987300	-0.49817200
H	0.13440500	1.21513300	2.68638600
H	1.37150200	-0.92507700	2.75946300
H	-3.05505700	0.42437100	0.86352500
H	-4.33316500	0.82126400	-1.88494800
H	-4.45779200	-1.18052700	1.36111500
H	-6.10978200	-0.52397600	-2.53966400
H	-7.74195700	-2.34818800	-2.13963300
H	-1.07598000	6.32821600	-0.75550500
H	4.37994000	0.99792800	-1.15172100
H	3.25133400	-2.14255000	1.76777600
H	-5.47998200	-2.90707700	2.57133100
H	-7.74405300	-4.13154400	0.90491600
Fe	4.95171500	-1.53865200	-0.31420000
O	6.24550100	-1.17268600	-1.78247900
H	7.09885700	-0.73584000	-1.66379200
H	6.06897000	-1.29897900	-2.72386700
O	6.32660800	-0.80318000	0.94694300
H	6.94139900	-1.35506300	1.44720200
H	6.35344100	0.09638800	1.29733100
O	3.71383500	-2.25467000	-1.71772100

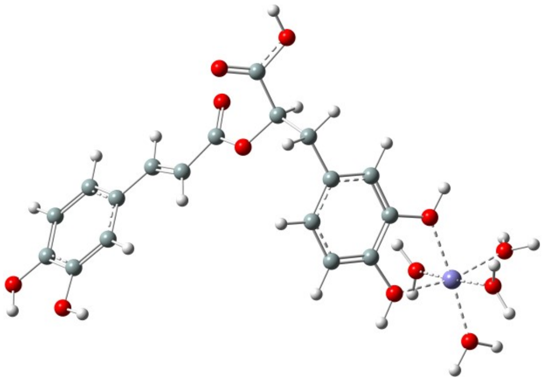

H	3.63256500	-3.19085200	-1.94034800
H	3.02824500	-1.75570900	-2.17976900
O	5.67119400	-3.37598200	-0.04665200
H	6.42158200	-3.71457100	-0.55386700
H	5.39153200	-4.04739300	0.59026500

Zero-point correction= 0.428128 (Hartree/Particle)
Thermal correction to Energy= 0.463963
Thermal correction to Enthalpy= 0.464908
Thermal correction to Gibbs Free Energy= 0.358085
Sum of electronic and zero-point Energies= -2866.277981
Sum of electronic and thermal Energies= -2866.242145
Sum of electronic and thermal Enthalpies= -2866.241201
Sum of electronic and thermal Free Energies= -2866.348023

RmAc-FeIII-4H2O-site4

3 6

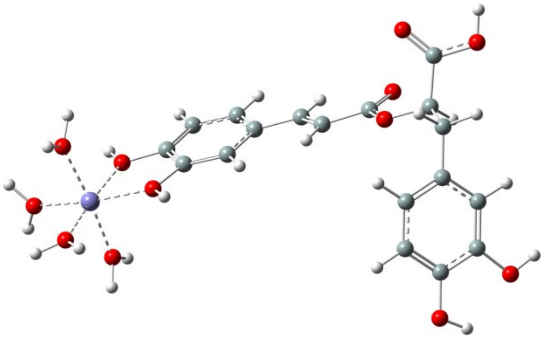

O	-3.37696100	-1.18975300	0.47470900
O	-6.15276500	-3.40191200	0.49073100
O	-6.73484000	3.07865000	-1.81924400
O	-5.02313100	4.74129500	-0.63058300
O	-4.18935900	-3.56166500	1.55454300
O	-2.98507200	-2.63469900	-1.19099700
O	3.72600200	0.54933100	0.58102400
O	5.25243000	-0.83542300	-0.83886200
C	-5.51685700	-0.54954600	1.29622100
C	-4.75570200	-1.52198100	0.39806400
C	-5.39606200	0.86709100	0.80467400
C	-6.15518800	1.29088000	-0.28405200
C	-4.51732800	1.75821800	1.39952800
C	-4.96378400	-2.94287600	0.87864200
C	-6.02932900	2.57893600	-0.76269400
C	-4.39108800	3.05663900	0.92134600
C	-5.14271500	3.47032100	-0.15933900
C	-2.57415000	-1.83121800	-0.39067700
C	1.19790600	-1.65392600	-0.94866400
C	-1.17459500	-1.41113700	-0.22153600
C	-0.23207800	-1.95566400	-0.98644300
C	1.71968300	-0.63650300	-0.14402700
C	2.05588500	-2.40963000	-1.74444200
C	3.07049000	-0.42750000	-0.16327800
C	3.42536400	-2.18848000	-1.75384200
C	3.91015300	-1.19053800	-0.94911000
H	-6.55835600	-0.86105800	1.31069300
H	-5.12343000	-0.63409400	2.30627900
H	-5.09917400	-1.44764700	-0.62942100
H	-6.85401900	0.61579100	-0.75907100
H	-3.92476400	1.44095900	2.24334600
H	-3.71173000	3.75670800	1.38109000
H	-0.98363500	-0.67097800	0.53725900
H	-0.53806000	-2.70151800	-1.70646900
H	1.08727800	-0.02174800	0.47485200
H	1.64774200	-3.18835800	-2.36752900

H	4.08603900	-2.77710700	-2.36894800	
H	-6.28321700	-4.28816700	0.85476800	
H	-7.31609500	2.41079500	-2.19150100	
H	-5.62193700	4.85398800	-1.37643500	
H	3.14158000	1.03686500	1.17832100	
H	5.85814800	-1.39046700	-1.35204100	
Fe	5.75679800	0.74123300	0.35167600	
O	5.53813000	1.96281300	-1.22116200	
O	7.67883500	0.59767400	-0.11920300	
H	8.10022500	1.02210600	-0.87799200	
H	8.32195800	0.05219600	0.35242000	
O	6.04111500	2.31795700	1.52810300	
H	5.39921600	2.80355800	2.06329400	
H	6.91195800	2.72446700	1.63743500	
O	6.14937800	-0.47942200	1.89168000	
H	6.28074100	-0.17141300	2.79772800	
H	6.03309100	-1.43808200	1.89646400	
H	5.40505400	1.69854700	-2.14014500	
H	5.49087000	2.92528700	-1.15611900	

Zero-point correction= 0.427440 (Hartree/Particle)
Thermal correction to Energy= 0.463385
Thermal correction to Enthalpy= 0.464330
Thermal correction to Gibbs Free Energy= 0.357388
Sum of electronic and zero-point Energies= -2866.274502
Sum of electronic and thermal Energies= -2866.238557
Sum of electronic and thermal Enthalpies= -2866.237613
Sum of electronic and thermal Free Energies= -2866.344554

Table S11: Cartesian coordinates and thermochemistry properties of ascobate mono-anion, ascobate radical, superoxide anion radical, oxygen molecule, neutral rosmarinic, mono-anion rosmarinate and aqueous iron complexes in water calculated at the M05-2X/6–311++G(2df,2p) level of theory.

Ascobate mono-anion

-1 1

C	1.86865700	-0.79372500	-0.03980100
C	1.66841900	0.60981100	-0.02349500
C	0.38662000	0.91073400	0.34235100
C	-0.31106700	-0.41431600	0.58858600
H	-0.61769100	-0.50262600	1.63050800
O	0.69194300	-1.41243600	0.33235400
O	2.65992300	1.50320100	-0.33964100
H	2.26758600	2.38116400	-0.29011500
O	-0.15831800	2.04671300	0.47536600
O	2.84917100	-1.46165900	-0.30974900
C	-1.50332500	-0.71305800	-0.30795700
H	-1.20226400	-0.58621200	-1.35009300
O	-1.93390800	-2.04656900	-0.07154000
H	-1.16483900	-2.62008000	-0.13031900
C	-2.70341000	0.17678900	-0.01965800
H	-2.88945900	0.18392900	1.05719300
H	-3.56590900	-0.27033900	-0.50520700
O	-2.55053000	1.48369200	-0.52653300
H	-1.72903700	1.85321800	-0.13418300

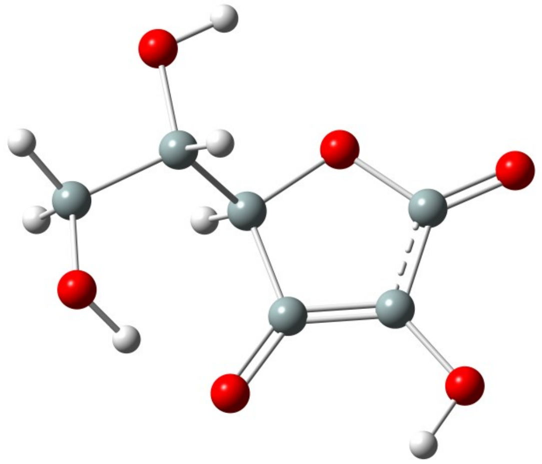

Zero-point correction= 0.139546 (Hartree/Particle)
Thermal correction to Energy= 0.150600
Thermal correction to Enthalpy= 0.151545
Thermal correction to Gibbs Free Energy= 0.102516
Sum of electronic and zero-point Energies= -684.389169
Sum of electronic and thermal Energies= -684.378115
Sum of electronic and thermal Enthalpies= -684.377170
Sum of electronic and thermal Free Energies= -684.426199

Ascobate radical

0 2

C	1.89097900	-0.81588200	-0.05583600
C	1.70423000	0.63351200	-0.02945100
C	0.36656100	0.93208600	0.35810800
C	-0.31038600	-0.40376600	0.59069700
H	-0.58910200	-0.50720000	1.63691500
O	0.71967000	-1.38323100	0.31077500
O	2.64888200	1.47908600	-0.32332100
H	2.32154000	2.38940100	-0.24362500
O	-0.11134000	2.04946300	0.48732400
O	2.87102300	-1.44224200	-0.33738900
C	-1.51425400	-0.72073500	-0.29047200
H	-1.23752400	-0.60558900	-1.33912300
O	-1.92503400	-2.04574500	-0.01582000
H	-1.20064900	-2.64684900	-0.20882200
C	-2.71702700	0.16651100	0.00764700

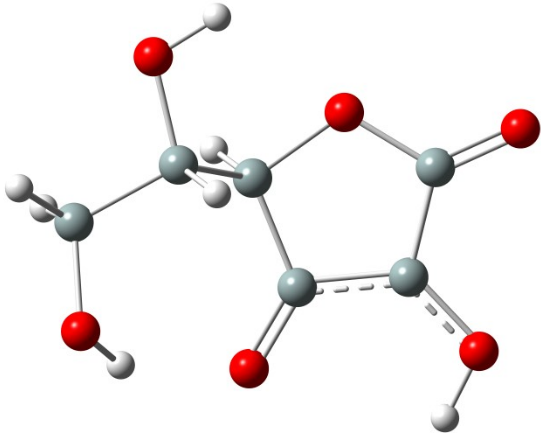

H	-2.85922600	0.24896500	1.08570800
H	-3.58452100	-0.33275000	-0.41049900
O	-2.63104600	1.43707300	-0.60276300
H	-1.94837400	1.94843900	-0.15514800

Zero-point correction= 0.140700 (Hartree/Particle)
Thermal correction to Energy= 0.151828
Thermal correction to Enthalpy= 0.152772
Thermal correction to Gibbs Free Energy= 0.102750
Sum of electronic and zero-point Energies= -684.199294
Sum of electronic and thermal Energies= -684.188165
Sum of electronic and thermal Enthalpies= -684.187221
Sum of electronic and thermal Free Energies= -684.237244

Superoxide anion radical

-1 2

O	0.00000000	0.00000000	0.65852500
O	0.00000000	0.00000000	-0.65852500

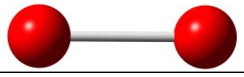

Zero-point correction= 0.002957 (Hartree/Particle)
Thermal correction to Energy= 0.005328
Thermal correction to Enthalpy= 0.006273
Thermal correction to Gibbs Free Energy= -0.016794
Sum of electronic and zero-point Energies= -150.476505
Sum of electronic and thermal Energies= -150.474133
Sum of electronic and thermal Enthalpies= -150.473189
Sum of electronic and thermal Free Energies= -150.496256

Oxygen

0 1

O	0.00000000	0.00000000	0.59240600
O	0.00000000	0.00000000	-0.59240600

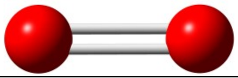

Zero-point correction= 0.003992 (Hartree/Particle)
Thermal correction to Energy= 0.006354
Thermal correction to Enthalpy= 0.007299
Thermal correction to Gibbs Free Energy= -0.014902
Sum of electronic and zero-point Energies= -150.284921
Sum of electronic and thermal Energies= -150.282558
Sum of electronic and thermal Enthalpies= -150.281614
Sum of electronic and thermal Free Energies= -150.303815

Neutral rosmarinic

0 1

O	-0.99828000	1.16713700	-0.40877700
O	-3.06487200	4.00592700	0.10596100
O	-5.22353800	-2.28091900	1.84111000
O	-4.13433600	-4.22306300	0.31708200
O	-1.23686400	3.77089400	-1.16537500
O	-0.11257400	2.27229000	1.32583800
O	5.47671000	-1.89462700	-1.75373400
O	7.38278500	-1.40626100	0.07315300
C	-3.28725500	1.20217700	-1.05879200
C	-2.23231600	1.82546400	-0.14939900
C	-3.52265600	-0.24221400	-0.71292300
C	-4.27876700	-0.56865000	0.40993400

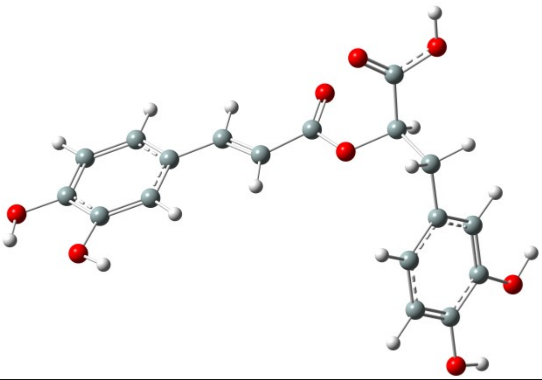

C	-2.97600400	-1.26213000	-1.47667000	
C	-2.09298300	3.29797100	-0.46005300	
C	-4.48425300	-1.88889600	0.75536800	
C	-3.18083600	-2.59234400	-1.13062300	
C	-3.93218700	-2.90859200	-0.01720500	
C	0.02275700	1.48232100	0.41380800	
C	3.64097700	0.30945800	0.55653600	
C	1.24032100	0.76620500	0.05191400	
C	2.34830600	0.93546600	0.78013600	
C	3.89884500	-0.51610100	-0.54428600	
C	4.65838800	0.54610000	1.47767700	
C	5.14051000	-1.08281300	-0.70530100	
C	5.90798600	-0.02989100	1.31827500	
C	6.15302700	-0.84413500	0.23022400	
H	-4.20345400	1.77461000	-0.93802600	
H	-2.95426500	1.29766900	-2.08942400	
H	-2.50740600	1.70957300	0.89440700	
H	-4.71780200	0.20770400	1.02173400	
H	-2.38877200	-1.02072500	-2.34926900	
H	-2.76215400	-3.39263600	-1.72068400	
H	1.18551900	0.11608700	-0.80550900	
H	2.29711800	1.60043300	1.63207800	
H	3.13952900	-0.71630200	-1.28527400	
H	4.46527300	1.18439100	2.32565700	
H	6.70093400	0.14540800	2.02771900	
H	-2.98300300	4.93590200	-0.15518500	
H	-5.57214400	-1.50854800	2.29931900	
H	-4.67808100	-4.26809300	1.11207200	
H	4.72760700	-1.99549600	-2.35114100	
H	7.39568400	-1.93737500	-0.73225800	

Zero-point correction= 0.322037 (Hartree/Particle)
Thermal correction to Energy= 0.345988
Thermal correction to Enthalpy= 0.346932
Thermal correction to Gibbs Free Energy= 0.265539
Sum of electronic and zero-point Energies= -1297.413030
Sum of electronic and thermal Energies= -1297.389079
Sum of electronic and thermal Enthalpies= -1297.388135
Sum of electronic and thermal Free Energies= -1297.469528

Rosmariniate mono-anion

-1 1

O	-1.00749900	1.17925700	-0.37465700	
O	-2.97442900	4.11751900	0.02508600	
O	-5.26889400	-2.17825000	1.84989900	
O	-4.24759500	-4.14461100	0.30972100	
O	-1.13722400	3.74153000	-1.19601800	
O	-0.11498300	2.23386300	1.38861100	
O	5.45716400	-1.89150600	-1.77326000	
O	7.37125700	-1.42283300	0.05184800	
C	-3.27767500	1.26529200	-1.06297300	
C	-2.23338800	1.88869400	-0.14664200	
C	-3.54627700	-0.17430300	-0.71974800	

C	-4.29729500	-0.48656000	0.41108000
C	-3.03589000	-1.20770000	-1.49129300
C	-2.08317500	3.38137600	-0.46254700
C	-4.53201300	-1.80218000	0.75601700
C	-3.26980600	-2.53371500	-1.14606500
C	-4.01567300	-2.83395000	-0.02481500
C	0.00582700	1.46893800	0.45043200
C	3.62827500	0.28447800	0.56934000
C	1.22640300	0.75518100	0.07624600
C	2.33481700	0.90739600	0.80575300
C	3.88344200	-0.52713500	-0.54206100
C	4.64887700	0.51019000	1.48945600
C	5.12510900	-1.09143200	-0.71408100
C	5.89883600	-0.06226500	1.31831400
C	6.14088100	-0.86306900	0.21990600
H	-4.18696400	1.85233000	-0.96005400
H	-2.93188100	1.34821500	-2.09117300
H	-2.52122800	1.77560000	0.89354200
H	-4.70948400	0.29856600	1.03048100
H	-2.45262000	-0.97965600	-2.37026200
H	-2.87802300	-3.34267700	-1.74289600
H	1.17192900	0.12354500	-0.79518000
H	2.28581500	1.55610500	1.67022400
H	3.12153500	-0.71912900	-1.28261100
H	4.45835500	1.13759200	2.34614300
H	6.69397200	0.10536300	2.02722600
H	-5.58251300	-1.39807600	2.31992700
H	-4.78309200	-4.17668500	1.11085800
H	4.70399600	-1.98904600	-2.36604000
H	7.38018100	-1.94720100	-0.75792800

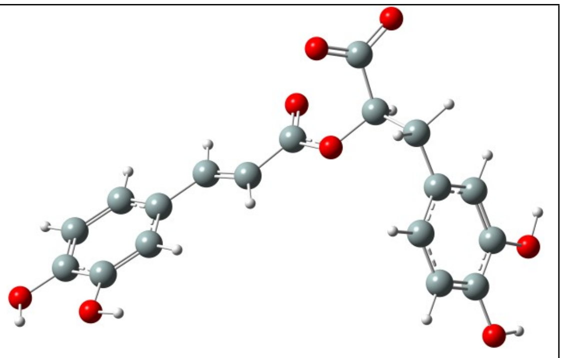

Zero-point correction= 0.309395 (Hartree/Particle)
Thermal correction to Energy= 0.333044
Thermal correction to Enthalpy= 0.333988
Thermal correction to Gibbs Free Energy= 0.252861
Sum of electronic and zero-point Energies= -1296.974876
Sum of electronic and thermal Energies= -1296.951226
Sum of electronic and thermal Enthalpies= -1296.950282
Sum of electronic and thermal Free Energies= -1297.031409

FeII-6H2O

2 5

Fe	0.00000000	0.00000000	0.00000000
O	-1.73050600	-0.65513200	-1.03728700
O	1.25491500	-0.87445900	-1.46880300
H	2.20997300	-0.81579000	-1.37371700
H	1.05736700	-1.70986300	-1.90159700
O	-1.25491500	0.87445900	1.46880300
H	-2.20997300	0.81579000	1.37371700
H	-1.05736700	1.70986300	1.90159700
O	-0.02330100	-1.80555200	1.11405100
H	0.74233000	-2.37901000	1.20932400
H	-0.53161400	-1.86271600	1.92818300

O	0.02330100	1.80555200	-1.11405100
H	0.53161400	1.86271600	-1.92818300
H	-0.74233000	2.37901000	-1.20932400
O	1.73050600	0.65513200	1.03728700
H	1.89221700	0.46635000	1.96592900
H	2.11885200	1.51320100	0.84351100
H	-1.89221700	-0.46635000	-1.96592900
H	-2.11885200	-1.51320100	-0.84351100

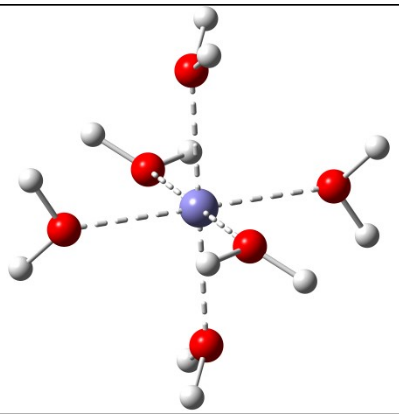

Zero-point correction= 0.148820 (Hartree/Particle)
Thermal correction to Energy= 0.167822
Thermal correction to Enthalpy= 0.168766
Thermal correction to Gibbs Free Energy= 0.102952
Sum of electronic and zero-point Energies= -1722.029978
Sum of electronic and thermal Energies= -1722.010976
Sum of electronic and thermal Enthalpies= -1722.010032
Sum of electronic and thermal Free Energies= -1722.075846

FeIII-6H2O

3 6

Fe	0.00031300	-0.00019300	-0.00022100
H	-0.11015600	-1.87980000	-1.90367300
H	0.90784900	-2.38279000	-0.80632300
H	-0.91015300	2.38074900	0.80629600
H	0.10945300	1.87940000	1.90304300
H	1.78091300	1.81218600	-0.84070400
H	2.02324100	0.49745400	-1.68004900
H	-2.24591100	0.45179700	-1.38117300
H	-1.07226200	1.16889900	-2.15544800
H	-2.02019400	-0.50191900	1.68113900
H	-1.77726800	-1.81546400	0.83986200
H	2.24634200	-0.45095300	1.38127100
H	1.07286700	-1.16712600	2.15645600
O	-1.29909800	0.64140700	-1.37896900
O	-0.31145600	1.66695200	1.06037800
O	1.49208300	0.90153300	-0.98206600
O	1.29871100	-0.63646600	1.38186300
O	-1.49174200	-0.90355700	0.97969700
O	0.30989600	-1.66829600	-1.06027300

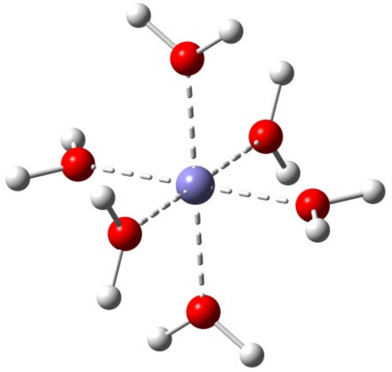

Zero-point correction= 0.150377 (Hartree/Particle)
Thermal correction to Energy= 0.166976
Thermal correction to Enthalpy= 0.167921
Thermal correction to Gibbs Free Energy= 0.107892
Sum of electronic and zero-point Energies= -1721.775048
Sum of electronic and thermal Energies= -1721.758449
Sum of electronic and thermal Enthalpies= -1721.757504
Sum of electronic and thermal Free Energies= -1721.817532

Table S12: Reaction enthalpies ($\Delta_r H^0$) and standard Gibbs free energies ($\Delta_r G^0$) and formation constants (K_f) of complexation reaction between the neutral rosmarinic (RA) and $[Fe(II).6H_2O]^{2+}$ and $[Fe(III).6H_2O]^{3+}$ ions in water phase at 298.15 K at the M05-2X/6–311++G(2df,2p) level of theory.

Chelating position	Fe^{2+} complexes			Fe^{3+} complexes		
	$\Delta_r H^0$	$\Delta_r G^0$	K_f	$\Delta_r H^0$	$\Delta_r G^0$	K_f
O2	3.1	8.1	1.20×10^{-6}	11.0	19.9	2.66×10^{-15}
O3	-1.6	8.0	1.46×10^{-6}	3.9	11.7	2.65×10^{-9}
O4	1.5	8.2	9.09×10^{-7}	9.0	14.1	4.79×10^{-11}
O5	-6.3	2.5	1.48×10^{-2}	-4.3	2.6	1.33×10^{-2}
O6	-5.2	1.5	7.35×10^{-2}	-5.7	1.2	1.36×10^{-1}
O7	1.2	6.4	2.12×10^{-5}	4.6	10.3	3.03×10^{-8}
O8	2.8	6.9	8.65×10^{-5}	10.6	16.7	5.33×10^{-13}
Site 1	1.9	-1.4	1.03×10^{1}	0.6	-3.3	2.8×10^{2}
Site 2	7.9	3.6	2.21×10^{-3}	14.2	10.8	1.21×10^{-8}
Site 3	6.5	-0.3	1.69×10^{0}	16.9	12.7	4.61×10^{-10}
Site 4	8.3	3.1	5.50×10^{-3}	18.7	14.5	2.43×10^{-11}

Table S13: The standard enthalpy ($\Delta_r\boldsymbol{H}^0$) and Gibbs free energy ($\Delta_r\boldsymbol{G}^0$) for the redox reaction between superoxide anion ($O_2^{\bullet-}$) and the iron complexes of neutral-rosmarinic form in water phase at the M05-2X/6–311++G(2df,2p) level of theory.

	(R9)		(R8)	
	$\Delta_r H^0$	$\Delta_r G^0$	$\Delta_r H^0$	$\Delta_r G^0$
Monodentate complexes				
O2	-45.8	-54.1	-44.8	-51.9
O3	-44.4	-46.8	-43.3	-44.7
O4	-44.9	-47.5	-43.9	-45.3
O5	-40.3	-42.6	-39.3	-40.4
O6	-37.5	-40.9	-36.5	-38.8
O7	-41.6	-46.3	-40.6	-41.1
O8	-45.4	-51.8	-44.4	-49.6
Bidentate complexes				
Site-1	-36.9	-40.6	-35.9	-38.4
Site-2	-44.3	-49.4	-43.3	-47.2
Site-3	-47.8	-54.7	-46.8	-52.5
Site-4	-48.6	-53.8	-47.6	-51.7
